# Hospital healthcare experiences of children and young people with life-threatening or life-shortening conditions, and their parents: scoping reviews and resultant conceptual frameworks

**DOI:** 10.1186/s12887-023-04151-6

**Published:** 2023-07-17

**Authors:** Suzanne Mukherjee, Natalie Richardson, Bryony Beresford

**Affiliations:** Social Policy Research Unit, School of Business and Society, University of York, York, YO10 5ZF UK

**Keywords:** Healthcare experiences, Patient experiences, Patient-reported experience measures, Children, Young people, Parents, Paediatrics, Life-threatening condition, Life-shortening condition, Scoping review

## Abstract

**Background:**

Patient experience is a core component of healthcare quality. Patient-reported experience measures (PREMs) are increasingly used to assess this, but there are few paediatric PREMs. This paper reports the first stage of developing two such measures, one for children and young people (0–18 years) (CYP) with a life-threatening or life-shortening condition (LT/LSC), and one for their parents. It comprised parallel scoping reviews of qualitative evidence on the elements of health service delivery and care that matter to, or impact on, CYP (Review 1) and parents (Review 2).

**Methods:**

Medline and PsychINFO (1/1/2010 – 11/8/2020) and CINAHL Complete (1/1/2010 – 4/7/2020) were searched and records identified screened against inclusion criteria. A thematic approach was used to manage and analyse relevant data, informed by existing understandings of patient/family experiences as comprising aspects of staff’s attributes, their actions and behaviours, and organisational features. The objective was to identity the data discrete elements of health service delivery and care which matter to, or impact on, CYP or parents which, when organised under higher order conceptual domains, created separate conceptual frameworks.

**Results:**

18,531 records were identified. Sparsity of data on community-based services meant the reviews focused only on hospital-based (inpatient and outpatient) experiences. 53 studies were included in Review 1 and 64 in Review 2. For Review 1 (CYP), 36 discrete elements of healthcare experience were identified and organized under 8 higher order domains (e.g. staff’s empathetic qualities; information-sharing/decision making; resources for socializing/play). In Review 2 (parents), 55 elements were identified and organized under 9 higher order domains. Some domains were similar to those identified in Review 1 (e.g. professionalism; information-sharing/decision-making), others were unique (e.g. supporting parenting; access to additional support).

**Conclusions:**

Multiple and wide-ranging aspects of the way hospital healthcare is organized and delivered matters to and impacts on CYP with LT/LSCs, and their parents. The aspects that matter differ between CYP and parents, highlighting the importance of measuring and understanding CYP and parent experience seperately. These findings are key to the development of patient/parent experience measures for this patient population and the resultant conceptual frameworks have potential application in service development.

**Supplementary Information:**

The online version contains supplementary material available at 10.1186/s12887-023-04151-6.

## Background

There is now wide recognition that, in addition to outcomes and safety, patient experience is a core component of healthcare quality and should be subject to the same degree of monitoring and bench-marking [1–3]. Dominant theoretical understandings, based almost exclusively on research with adult patients and their family members, have specified the dimensions of healthcare delivery which matter to patients and affect how they experience using healthcare. Broadly similar across conceptual models, these include: respect for patient values and preferences; coordination and integration of care; information and communication; physical comfort; emotional support and involvement of family and friends [4, 5]. These dimensions of experience are found in many of the patient-reported experience measures (PREMs) which have been developed in recent years (e.g. [6–9]).

However, some have questioned whether the dominant frameworks properly capture what really matters to patients and families [10, 11]. Others have critiqued the frameworks (and the associated PREMs) as focusing on experiences of the functional aspects of care (e.g. timely administration of medication, availability of information, ward facilities) and neglecting the relational dimension of patient experience. That is, how interactions and relationships with healthcare staff are experienced. In response, a number of smaller bodies of literature (and resultant PREMs) have emerged focusing on relational experience [12–14].

The conceptual and measurement limitations described above are as true for PREMs used in paediatric settings as they are for those used with adult and older patients. However, within the paediatric context, there are additional concerns. In terms of measuring the child’s experience, the majority of paediatric PREMs are parent proxy, rather than child—report [15]. In addition, among the child-report measures which do exist, many have not been developed from research with children, with some simply a re-wording of an adult PREM into ‘child-friendly’ text and response formats, with no investigation into whether these aspects of the care experience matter to children and young people (CYP), or indeed whether what matters differs according age and cognitive ability (e.g. [16]. Furthermore, attempts to develop measures of parents’ own experiences have, to date, been extremely limited [17]. This is somewhat surprising given the core and essential roles parents play as medical, physical and emotional care providers, decision-makers and care navigators. The origins of PREMs in adult medicine are likely to have contributed to the neglect, or lack of recognition, of this aspect of healthcare experience. To date, attempts to develop robust and meaningful parent experience measures (PaREMs) have been hampered by developers failing to adhere to measure development guidelines, and are typically specific to particular healthcare settings (e.g. critical care) [17].

Within the paediatric population, the heaviest users of healthcare are CYP with life-threatening illnesses (e.g. cancers; severe congenital heart disease) and conditions which shorten life expectancy due to the degenerative nature of the condition, or because the condition significantly increases the risk of health complications (e.g. seizures, severe respiratory infections). Life expectancy can be very short—within minutes or hours of birth (e.g. Edwards’ syndrome (trisomy 18)) – through to the likelihood of death occurring sometime during the childhood years or adulthood (e.g. Duchenne muscular dystrophy; severe cerebral palsy) [18–20]. Given the frequency and intensity of health service use among this population and the fact that, given the prognosis, healthcare experiences are likely to be even more salient, there is a strong case for better understanding their experiences, and for developing a CYP’s PREM and PaREM for this population.

This paper describes the first stage in the development of a PREM for CYP with a life-threatening or life-shortening condition (LT/LSC), and a PaREM for parents of such children. It comprised parallel scoping reviews of qualitative studies investigating: (1) the healthcare experiences of CYP (aged 0–18 years) with a LT/LSC and (2) the healthcare experiences of their parents in their role as parent (referred to as Review 1 and Review 2 respectively).

## Methods

The overall objective of the reviews was to identify and define aspects (or elements) of health service delivery and care that matter to, or impact on, CYP aged 0–18 years with a LT/LSC or their parents, and to present these as conceptual frameworks. A second objective (added early in the data extraction stage) was to identify and synthesise evidence on the ways health service delivery and care impact on CYP and parents. The reviews, carried out in accordance with current guidance, are reported according to the PRISMA extension for scoping reviews [21, 22].

### Review questions

Review 1:

- What aspects (or elements) of health service delivery and care matter to, or impact, CYP aged 0–18 years with a LT/LSC?

Review 2:

- What aspects (or elements) of health service delivery and care matter to, or impact, parents of CYP aged 0–18 years with a LT/LSC in their caring role?

In addition, there were two cross-cutting questions:

- Do the aspects of health service delivery and care that matter differ between CYP and parents?
- Do the aspects of health service delivery and care that matter differ according to age and/or developmental stage of the CYP?

At the time the reviews were conducted, PROSPERO was not registering scoping reviews. The protocol was therefore published in an alternative repository [23].

### Parent and professional involvement

Two advisory groups supported the reviews. The first was the Martin House Research Centre’s Family Advisory Board (www.york.ac.uk/healthsciences/research/public-health/projects/martinhouse/mh-ppi/fab/) which is comprised of parents of CYP with LT/LSC, including bereaved parents. Consulted at study outset, the group confirmed the importance of including studies where parents were acting as proxies for their children (due to significant cognitive impairment and/or health status/medical fragility). They were also consulted during data analysis when emergent findings were presented, particularly for the purpose of checking data interpretation. A second advisory group comprised clinicians and academics/researchers working in the field of paediatric palliative care. This group was consulted on three occasions during data analysis and the development of the conceptual frameworks.

### Eligibility criteria

Inclusion criteria were:

- concerns CYP up to 18 years of age diagnosed with a LT/LSC as specified in the search strategy;
- study focus is experiences of using a health service(s);
- reports primary research using qualitative methods or mixed methods in which qualitative data is reported separately;
- data gathered directly from CYP and/or parents;
- sample size of at least 5;
- published in English in a peer-reviewed journal;
- published in 2010 or more recently.

Exclusion criteria were:

- no data collected on experiences of using a healthcare service (e.g. studies of lived experience, evaluations of specific interventions etc.);
- only concerned with one or more of the following types of healthcare: cancer long-term follow-up, ante-natal, maternity, bereavement, ambulance services;
- only concerned with the diagnostic process, adult healthcare and/or the transfer to adult healthcare, or does not report paediatric and adult healthcare experiences separately;
- Masters / Doctoral Dissertations, books, book chapters, conference posters, and unpublished studies;
- studies conducted in non-OECD countries;
- published before 2010.

### Information sources and search strategy

The following databases were searched: Medline and PsychINFO (search date ranges: 1/1/2010 – 11/8/2020, and CINAHL Complete (1/1/2010 – 4/7/2020). Search strategies were constructed around the following concepts: [LT/LSCs (specific diagnoses, diagnostic categories)]; [healthcare setting or healthcare staff]; [study design (qualitative or mixed methods] and [population (children and/or parent)]. LT/LSCs (*n* = 282) included in searches was based on previous work. Full search strategies are provided in Supplementary File 1.

### Quality appraisal

As this was a scoping review a quality appraisal of included studies was not undertaken.

### Study selection

Once de-duplicated, search outputs were uploaded into Covidence, an online software designed for managing systematic reviews [24]. Two reviewers independently screened all records by title and abstract. A third reviewer was consulted when differences of opinion regarding inclusion/exclusion could not be resolved. Full texts of retained articles were retrieved and the same screening process implemented and reasons for exclusion recorded. At this point it was noted that, for both reviews, almost all retained articles concerned hospital (inpatient and outpatient) healthcare experiences, as opposed to community-based health services. Furthermore, an initial review of the themes reported in studies revealed the healthcare experiences reported in the community-based health service studies were quite different to those reported elsewhere, focusing primarily on access to services and the skills of those providing them. The decision was therefore taken to limit the reviews to hospital (inpatient and outpatient) experiences only.

### Data extraction and analysis

Data analysis used the core devices of a thematic approach to qualitative data analysis: data immersion, data display, data reduction and analytical writing [25].

#### Data extraction

All members of the review team (SM, NR, BB) were involved in developing the data extraction templates. In terms of study characteristics, the following data was extracted for both reviews: study aims, country, research design, method(s), study population (e.g. parent vs CYP (Review 1 only), gender, CYP’s age, type ofLT/LSC, ethnicity, healthcare setting, stages(s) in condition trajectory, and sample size.

Data extraction templates (or thematic frameworks) for research findings were informed by existing conceptual models of healthcare experience [4, 5, 26], domains measured by existing adult and CYP PREMs, and scrutiny of sub-sets of included studies (at least 10 per review) purposively selected to represent different study objectives, a range of diagnoses, and stages of the illness trajectory. For Review 1, the sub-set also included CYP versus parent study participant. Once drafted, initial templates were subject to an iterative process of testing and revising, involving all three reviewers using the same sub-set of studies. Final versions of the data extraction frameworks are provided in Supplementary File 2.

Relevant data from the included articles was extracted *ad verbatim* (i.e. original author’s text, study participant quotes). Data was deemed relevant if study authors reported an experience of health service delivery or care as mattering to one or more study participants: that is, it was of importance or significance to the CYP or parent, and/or had positive or negative impacts.

For both reviews, two researchers (SM, NR) independently extracted from 10% of included studies to confirm shared understanding of themes. Data from remaining included studies were extracted by one reviewer. Once completed, one reviewer (SM) scrutinised data extraction for both reviews to check for accuracy and consistency of allocation of data to themes. All reviewers (SM, NR, BB) then met to resolve queries and inconsistencies.

#### Main analysis

For both reviews, our approach to analysis was informed by the work of Entwistle et al. [10] who argue that conceptual frameworks underpinning the measurement of patient experience need to be more fine-grained if they are to be useful in identifying what needs to be done to improve service quality. They also make the case that, to better understand the relational dimension of patient experience, measures should focus on staff attributes (i.e. characteristics, inherent qualities) and actions, and not the feelings which interactions, or relationships, with staff generate.

To start, the completed data extraction templates were independently examined by all members of the review team. Following this the team met to discuss the next analytical iteration. For both reviews it was agreed that, within each theme, extracted data could usefully be categorised as describing one or more of the following: staff attributes, staff actions and organisational features (i.e. features of service organisation or delivery, hospital facilities).

Individual thematic tables were therefore constructed which, along a row, presented the *ad verbatim* data and summaries of that data organised into descriptions of staff attributes, descriptions of staff actions and descriptions of organisational features, see Table 1. One reviewer (SM) coded and summarised all the extracted data into these categories. Other members of the review team (NR, BB) then independently checked the summaries in terms of coding and the closeness to the original data. The team then met to discuss the summaries, after which corrections and changes were implemented by SM.

**Table 1:** Structure of thematic tables

Theme title:				
Study ID	Verbatim data extract	*Review team summaries of data extract*		
		Staff attributes	Staff actions	Organisational features

Following this, an overarching summary of the data contained within a theme was drafted by one reviewer (SM) which described the discrete elements of health service delivery and care identified by the analysis. Summaries were reviewed and commented on at least once by at least one other member of the team.

Next the summaries were refined into one or more short statements, each describing a discrete element of health service care and delivery, and categorised as either a staff attribute, staff action or organisational feature. Mindful that these statements would form the basis of a PREM, a specific rubric was used to guide how they were worded, see Table 2.

**Table 2:** Statements describing the elements of healthcare experience: writing style rubric

• One sentence describing a single construct
• Simple, unambiguous wording
• Positively phrased
• Where possible using words/phrases commonly found in verbatim quotes
• No colloquialisms
• Applicable to any hospital healthcare setting or staff group
• Worded so as not to imply a particular age or developmental stage
• Non-gendered

Finally, for each review, all the ‘elements of healthcare experience’ statements which had been generated were subject to an iterative process of review and discussion by the team with the objective of grouping them under higher order concepts, or domains of health service care and delivery. This process was continued until the team was satisfied with the conceptual validity of the frameworks.

#### Secondary analysis: mapping the impacts of healthcare experiencess

Extracted data was re-examined for descriptions of the impact(s) of the elements of health service delivery and care on CYP (Review 1) and parents’ (Review 2). An additional column was added to the thematic tables into which one reviewer (SM) entered a summary of any impact(s) reported by a study. For an example of a thematic table, with data extracted into it (including illustrative quotations) and impact summaries, please see Supplementary File 3.

Once completed the entire team reviewed the summaries and, through an iterative process, higher order concepts (or themes)—each capturing different types of impact—were agreed (see Supplementary Files 4 and 5). Impact summaries were then coded and coded data organized under these higher order concepts.

## Results

**Fig. 1:**
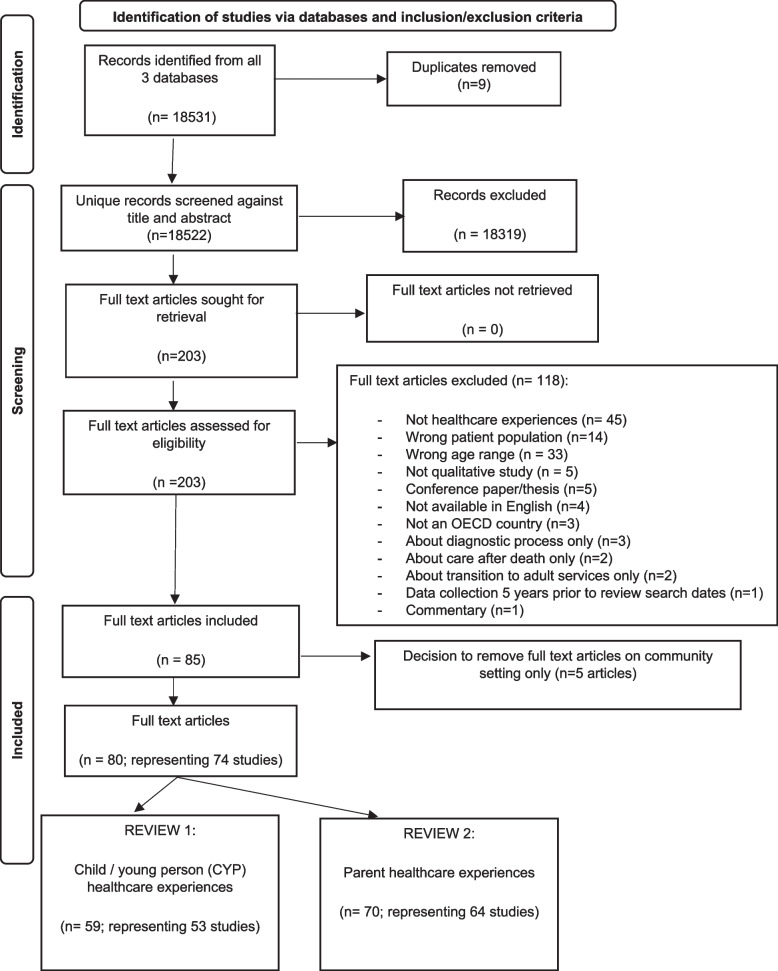
PRISMA 2020 flow diagram for scoping review

A total of 18,531 records were initially screened for Review 1 and/or Review 2, with 18,328 excluded. Full text articles for all remaining records (*n* = 203) were retrieved, of which 85 met inclusion criteria. As previously discussed (see methods section), at this stage the decision was taken to exclude 5 articles (representing 4 studies) only reporting on experiences of community-based health services, thereby making hospital healthcare experiences the focus of the review. Of the 80 articles (74 studies) taken forward into the review, 59 articles (representing 53 studies) were included in Review 1, and 70 articles (representing 64 studies) included in Review 2, see Fig. 1. Characteristics of included studies are set out in Table 3. Findings from the two reviews are reported sequentially.

**Table 3:** Characteristics of included studies

	**Review**	**Diagnosis**	**Setting reported on**	**Point(s) in trajectory**	**Focus on particular experience?**	**Study design, no. sites**	**Data collection method(s)**	**Child sample: Sample size, age range**	**Parent sample: Sample size, age range of children**
Anderson et al. (2018)[27] Australia	1,2	Neutropenia	Inpatient	Treat/ manage	No	Cross-sectional, Single centre	Interview	n/a	*N* = 9 (*n* = 8 mothers, *n* = 1 father) 0 – 14 yrs
Angstrom -Brannstrom et al. (2014)[28] Sweden	1	Cancers	Inpatient	Treat/ manage	No	Cross-sectional, Single centre	Interview	*N* = 9 (*n* = 5 boys, *n* = 4 girls) 3 – 9 yrs	n/a
Baenzinger et al. (2020)[29] Australia	1,2	Cancers	Inpatient, Outpatient	Treat/ manage	No	Cross-sectional, Multi-centre (*n* = 8)	Interview	n/a	*N* = 58 (*n* = 52 mothers, *n* = 6 fathers) 0 – 15 yrs
Bergviken & Nilsson (2019)[30] Sweden	2	Cancers	Outpatient	Treat/ manage	No	Cross-sectional, Single centre	Interview	n/a	*N* = 17 (*n* = 11 mothers, *n* = 6 fathers) 1–15 yrs
Bailey-Pearce et al. (2018) [31] UK	2	Mixed LT/LSCs	Inpatient	Not reported	No	Cross-sectional, Single centre	Interview	n/a	*N* = 7 (*n* = 7 fathers) Child age: not reported
Baird et al. (2015, 2016)[32, 33] USA	1,2	Mixed LT/LSCs	Inpatient (PICU)	Critical episode	R’ships with staff	Cross-sectional, Single centre	Interview	n/a	*N* = 7 (*n* = 5 mothers, *n* = 2 fathers) 0 – 15 yrs
Ballantyne et al. (2019)[34] Canada	2	Cerebral palsy	Inpatient	Diagnosis; treat/ manage	No	Cross-sectional, Single centre	Interview	n/a	*N* = 18 (*n* = 13 mothers, *n* = 5 fathers) Child age: not reported
Baugham et al. (2017)[35] USA	2	Mixed LT/LSCs	Inpatient(NICU)	End of life; end stage	No	Cross-sectional, Single centre	Interview	n/a	*N* = 45 (*n* = 29 mothers, *n* = 16 fathers) Mean age at death: 41 days
Brooten et al. (2013) [36] USA	1,2	Mixed LT/LSCs	Inpatient (NICU, PICU)	End stage	No	Cross-sectional, Multi-centre (*n* = 5)	Interview	n/a	*N* = 63 (*n* = 44 mothers, *n* = 19 fathers) Mean age at death: 43 mos
Brouwer et al. (2020)[37] Netherlands	1,2	Mixed LT/LSCs	Inpatient, Outpatient	Treat/ manage; end of life; end stage	No	Cross-sectional, Multi-centre (no. sites not reported)	Interview	n/a	*N* = 64 ( parent unspecified) 1–12 yrs
Butler et al. (2018a, 2018b, 2018c, 2019)[38–41] Australia	1,2	Mixed LT/LSCs	Inpatient (PICU)	End stage	R’ships with staff	Cross-sectional, Multi-centre (*n* = 4)	Interview	n/a	*N* = 26 (*n* = 18 mothers, *n* = 8 fathers) Child age: not reported
Callans et al. (2016) [42] USA	1,2	Not specified (healthcare technology-dependent)	Inpatient	Not reported	Care transitions	Cross-sectional, Single centre	Focus group	n/a	*N* = 18 (*n* = 16 mothers, *n* = 2 fathers) Child age: not reported
Carnevale (2013)[43] Canada	1	Mixed LT/LSCs	Inpatient (PICU)	Critical episode	Care transitions	Cross-sectional, Single centre	Interview	*N* = 12 (*n* = 8 boys, *n* = 4 girls) 3 – 17 yrs	n/a
Carnevale et al. (2011)[44] Italy	1,2	Mixed LT/LSCs	Inpatient (PICU)	Critical episode	Decision-making	Cross-sectional, Multi-centre (*n* = 2)	Interview, focus group	n/a	*N* = 7 (*n* = 7 mothers) 1 mos – 14 yrs
Cicero-Cinecto et al. (2017) [45] Mexico	1,2	Cancers	Inpatient	End of life; end stage	Decision making	Cross-sectional, Multi-centre (*n* = 3)	Interview	*N* = 6 (*n* = 4 boys, *n* = 2 girls) 13 – 18 yrs	*N* = 13 (*n* = 10 mothers, *n* = 3 fathers) Child age: not reported
Coats et al. (2016)[46] USA	2	Not specified (Bone marrow transplant recipients)	Inpatient, Outpatient	Treat/ manage; end of life	Decision-making	Cross-sectional, Single centre	Interview	n/a	*N* = 7) (*n* = 5 mothers, *n* = 2 fathers) 2 – 8 yrs
Conway et al. (2017)[47] USA	1,2	Cancers	Inpatient	Treat/ manage	No	Cross-sectional, Single centre	Interview	n/a	*N* = 50 (*n* = 48 mothers, *n* = 2 fathers) Child age: not reported
Coyne et al. (2014)[48] Ireland	1,2	Cancers	Inpatient, Outpatient	Treat/ manage	Decision-making	Cross-sectional, Single centre	Interview	*N* = 20 (*n* = 11 boys, *n* = 9 girls) 7 – 16 yrs	*N* = 22 (*n* = 17 mothers, *n* = 5 fathers) Child are: not reported
Dahav et al. (2018)[49] Sweden	1,2	Mixed LT/LSCs	Inpatient (PICU)	Critical episode	No	Cross-sectional, Single centre	Interview	n/a	*N* = 15 (*n* = 10 mothers, *n* = 5 fathers) 0 – 16 mos
Darbyshire et al. (2015)[50] Italy	2	Mixed LT/LSCs	Inpatient, Outpatient	Diagnosis; end stage	No	Cross-sectional, Multi-centre (no. sites not reported)	Interview, qualitative survey	n/a	*N* = 33 (*n* = 17 mothers, *n* = 16 fathers) Child age: not reported
Davies et al. (2017)[51] UK	1,2	Cancer: leukaemia	Outpatient	Treat/ manage	R’ships with staff	Longitudinal Multi-centre (*n* = 6)	Interview	n/a	*N* = 20 (*n* = 8 mothers, *n* = 12 fathers) 1–12 yrs
Engler et al. (2020)[52] Germany	1,2	Not specified (Received paediatric palliative care)	Inpatient	End of life; end stage	No	Cross-sectional, Single centre	Interview	n/a	*N* = 13 (*n* = 9 mothers, *n* = 4 fathers) 4 mos – 17 yrs
Engvall et al. (2016)[53] Sweden	1	Cancers	Outpatient	Treat/ manage	No	Cross-sectional, Multi-centre (*n* = 3)	Interview	*N* = 13 (*n* = 6 boys, *n* = 7 girls) 5 – 15 yrs	n/a
Enskar et al. (2020)[54] Sweden	1,2	Cancers	Inpatient, Outpatient	Diagnosis; treat/ manage	Nurses’ caring practices	Longitudinal Single centre	Interview	*N* = 25 (*n* = 10 boys, *n* = 15 girls) 3—6 yrs	*N* = 38 (*n* = 24 mothers, *n* = 14 fathers) 1 – 6 yrs
Falck et al. (2016)[55] USA	1,2	Mixed LT/LSCs	Inpatient (NICU)	Critical episode	No	Cross-sectional, Single centre	Interview	n/a	*N* = 6 (*n* = 6 mothers) 25–34 wks
Falkenburg et al (2016, 2018)[56, 57] Netherlands	1,2	Mixed LT/LSCs	Inpatient (PICU)	End of life; end stage	Environment/ R’ships with staff	Cross-sectional, Single centre	Interview	n/a	*N* = 36 (*n* = 19 mothers, *n* = 17 fathers) 2 wks – 14 yrs
Fixter et al. (2017)[58] UK	2	Cystic fibrosis	Inpatient	Treat/ manage	No	Cross-sectional, Single centre	Interview	n/a	*N* = 12 (*n* = 10 mothers, *n* = 2 fathers) 2–14 yrs
Gabriel et al. (2019)[59] Australia	1,2	Cancers	Inpatient, Outpatient	Treat/ manage	Receiving surgery	Cross-sectional, Multi-centre (*n* = 11)	Interview	*N* = 17 (*n* = 7 boys, *n* = 10 girls) Age not reported	*N* = 15 (*n* = 7 mothers, *n* = 8 fathers) 0–15 yrs
Gilmer et al. (2013)[60] USA	2	Mixed LT/LSCs	Inpatient (inc. PICU, NICU)	End of life	No	Cross-sectional, Multi-centre (*n* = 3)	Interview	n/a	*N* = 15 (*n* = 1 mother, *n* = 14 fathers) “Infants to 14 yrs”
Greenway et al. (2019)[61] USA	1,2	Not specified (Admitted to PICU)	Inpatient (PICU)	Critical episode	Communication	Cross-sectional,	Interview	n/a	*N* = 52 (*n* = 33 mothers, *n* = 15 fathers, *n* = 4 legal guardians) 2 days—12 yrs
Guttman et al. (2020)[62] USA	2	Cerebral palsy	Inpatient	Diagnosis; critical episode	Communication	Cross-sectional, Multi-centre (*n* = 2)	Qualitative survey	n/a	*N* = 266(parent unspecified) Child age: not reported
Hemsley et al. (2013)[63] Australia	1	Cerebral palsy	Inpatient, Outpatient	Not reported	Communication	Cross-sectional, Multi-centre: no. sites not reported	Focus group	*N* = 6 (*n* = 4 boys, *n* = 2 girls) 13 – 18 yrs	*N* = 10 (*n* = 9 mothers, *n* = 1 father) Child age: not reported
Hooghe et al. (2018)[64] Belgium	2	Cancers	Inpatient Outpatient	Treat/ manage	No	Cross-sectional, Single centre	Interview, focus group	n/a	*N* = 16 (*n* = 9 mothers, *n* = 7 fathers) Age range: 9 mos – 15 yrs
Inglin et al. (2011)[65] Switzerland	1,2	Mixed LT/LSCs	Inpatient, Outpatient	Diagnosis; treat/ manage; end stage	No	Cross-sectional, Multi-centre (*n* = 4)	Interview	n/a	*N* = 17 (*n* = 15 mothers, *n* = 2 fathers) 1—18 yrs
Iversen et al. (2013)[66] Norway	1,2	Cerebral palsy	Inpatient	Treat/ manage	Surgery	Cross-sectional, Single centre	Interview	n/a	*N* = 12 (*n* = 6 mothers, *n* = 6 fathers) 8—16 yrs
Kelly et al. (2017)[67] USA	1	Cance	Inpatient, Outpatient	Not reported	Decision-making	Cross-sectional, Single centre	Interview	*N* = 29 (*n* = 15 boys, *n* = 14 girls) 9 – 17 yrs	n/a
Kilicarslan-Toruner and Akgun- Citak (2013) [68] Turkey	2	Cancers	Inpatient, Outpatient	Treat/ manage	Decision-making	Cross-sectional, Single centre	Interview	n/a	*N* = 15 (*n* = 13 mothers, 2 fathers). 2–18 years
Lamiani et al. (2013)[69] Italy	2	Mixed LT/LSCs	Inpatient (PICU)	End of life; end stage	No	Cross-sectional,	Interview	n/a	*N* = 8 (*n* = 5 mothers, *n* = 3 fathers) 2mos – 13 yrs
Linder et al. (2017)[70] USA	1	Cancers	Inpatient	Treat/ manage	“Sources of bother”	Cross-sectional, Single centre	Qualitative survey	*N* = 50 (*n* = 23 boys, *n* = 27 girls) 7–18 yrs	n/a
Livesley and Long (2013)[71] UK	1	Mixed LT/LSCs	Inpatient	Treat/ manage	No	Cross-sectional, Single centre	Interview	*N* = 16 (*n* = 7 boys, *n* = 9 girls) 5–16 yrs	n/a
Mack et al. (2017)[72] USA	1,2	Cancers	Inpatient	Not reported	R’ships with staff	Cross-sectional, Multi-centre (*n* = 2)	Interview	n/a	*N* = 29 (*n* = 18 mothers, *n* = 10 fathers, *n* = 1 missing data) Child age: not reported
Markwalter et al. (2019)[73] USA	2	Not specified (Admitted to PICU)	Inpatient (PICU)	Critical episode	Care transitions	Cross-sectional, Single centre	Interview	n/a	*N* = 25 (*n* = 25 mothers) 7 mos – 9yrs
McNamara et al. (2020)[74] USA	2	Mixed LT/LSCs	Inpatient	Treat/ manage	Religious & spiritual care	Cross-sectional, Single centre	Interview	n/a	*N* = 19 (*n* = 19 mothers) Child age: not reported
Mitchell et al. (2019)[75] UK	1,2	Mixed LT/LSCs	Inpatient (PICU)	End of life; end stage	Decision-making	Cross-sectional, Single centre	Interview	n/a	*N* = 17 (*n* = 11 mothers, *n* = 6 fathers) 5 mos—18 yrs
Murrell et al. (2018)[76] USA	1,2	SMA: Type 1	Inpatient, Outpatient	Not reported	No	Cross-sectional, Multi-centre (no. sites not reported)	Interview	n/a	*N* = 29 (*n* = 18 mothers, *n* = 11 fathers) 6 mos – 14 yrs
Nicholas et al. (2016)[77] Canada	2	Mixed LT/LSCs	Inpatient	Diagnosis; treat/ manage; end of life; end stage	No	Cross-sectional, Single centre	Interview, focus group	n/a	*N* = 18 (*n* = 18 fathers) Child-age: not reporte d
Nyborn et al. (2016)[78] USA	1,2	Cancers	Outpatient	Critical episode	Communication	Cross-sectional, Single centre	Interview	n/a	*N* = 28 (*n* = 26 mothers, *n* = 2 fathers) 2.5 – 17.5 yrs
Obas et al. (2016)[79] Canada	1,2	Cardiac disease	Inpatient (PICU)	Treat/ manage	Care transitions	Cross-sectional, Single centre	Interview	n/a	*N* = 9 (parent unspecified) 2 mos – 14 yrs
October et al. (2014)[80] UK	2	Mixed LT/LSCs	Inpatient (PICU)	Critical episode	Decision-making	Cross-sectional, Single centre	Qualitative survey	n/a	*N* = 43 (*n* = 25 mothers, n = 18 fathers) 1.4 – 10 yrs
Orioles et al. (2013)[81] USA	1,2	Mixed LT/LSCs	Inpatient(inc. PICU)	Diagnosis	Communication	Cross-sectional, Single centre	Interview	n/a	*N* = 13 (*n* = 12 mothers, *n* = 1 father) “Infants”—18 yrs
Oxley (2015)[82] UK	2	Not specified (Admitted to PICU)	Inpatient (PICU)	Critical episode	No	Cross-sectional, Single centre	Interview	n/a	*N* = 7 (n = 6 mothers, *n* = 1 father) Child age: not reported
Pinto-Taylor et al. (2020)[83] USA	1,2	Mixed LT/LSCs	Inpatient (inc PICU), Outpatient	Diagnosis; end of life; end stage	Decision-making	Cross-sectional, Single centre	Interview	n/a	*N* = 9 (parent unspecified) Child age: not reported
Robertson et al. (2019)[84] Australia	1,2	Cancers	Inpatient, Outpatient	Not reported	Decision-making	Cross-sectional, Single centre	Interview	*N* = 5 (*n* = 4 boys, *n* = 1 girl) 11—15 yrs	*N* = 25 (= 23 mothers, *n* = 2 fathers) 8mos – 11 yrs
Roscigno et al. (2016)[85] USA	1,2	Traumatic brain injury	Inpatient	Critical episode	Nurses’ caring practices	Longitudinal Single centre	Interview	n/a	*N* = 29 (*n* = 25 mothers, *n* = 4 fathers) 6- 18 yrs
Ruhe et al. (2016)[86] Switzerland	1	Cancers	Outpatient	Treat/ manage	Decision-making	Cross sectional Multi-centre (*n* = 9)	Interview	*N* = 17 (*n* = 11 boys, *n* = 6 girls) 9—17yrs	n/a
Saetrang et al. (2019)[87] Norway	1,2	Duchenne muscular dystrophy	Outpatient	Treat/ manage	No	Cross-sectional, Single centre	Interview	n/a	*N* = 14 (*n* = 7 mothers, *n* = 7 fathers) 7 – 17 yrs
Salmon et al. (2012)[88] UK	1,2	Cancer: leukaemia	Outpatient	Not reported	No	Longitudinal Multi-centre (*n* = 6)	Interview	n/a	*N* = 53 (*n* = 31 mothers, *n* = 22 fathers) 1 – 12 yrs
Skirko et al. (2020)[89] USA	2	Pierre Robin Sequence	Inpatient, Outpatient	Not reported	No	Cross-sectional, Single centre	Interview, focus group	n/a	*N* = 16 (*n* = 11 mothers, *n* = 5 fathers) “ < 5 yrs”
Smith et al. (2015)[90] UK	1,2	Hydro-cephalus	Inpatient, Outpatient	Diagnosis; treat/ manage	Decision-making	Cross-sectional, Multi-centre (*n* = 2)	Interview	n/a	*N* = 25 (*n* = 15 mothers, *n* = 10 fathers) 2 – 13 yrs
Smith et al. (2018)[91] UK	2	Renal disease	Inpatient	Treat/ manage	Decision-making	Cross-sectional, Single centre	Interview	n/a	*N* = 10 (*n* = 6 mothers, *n* = 4 fathers) 18 – 28 mos
Snaman et al. (2016)[92] USA	1,2	Cancers	Inpatient	End of life	Communication	Cross-sectional, Single centre	Focus group	n/a	*N* = 12 (parent unspecified) Child age: not reported
Spalding et al. (2016)[93] UK	1,2	Not specified (transferred to hospice services)	Inpatient	Not reported	No	Cross-sectional, Single centre	Interview, focus group	*N* = 7 (*n* = 5 boys, *n* = 2 girls) 8 – 14 yrs	*N* = 5( mothers) Child age: not reported
Spratling et al. (2012)[94] USA	1	Not specified (required respiratory assistance)	Outpatient	Treat/ manage	No	Cross-sectional, Single centre	Interview	*N* = 11 (n = 5 boys, *n* = 6 girls) 13 – 18 yrs	n/a
Steele et al. (2013)[95] USA & Canada	1,2	Cancers	Inpatient	End of life; end stage	No	Cross-sectional, Multi-centre (*n* = 3)	Interview	n/a	*N* = 60 (*n* = 36 mothers, *n* = 24 fathers) 8 – 17 yrs
Sullivan et al. (2014)[96] Australia	2	Mixed LT/LSCs	Inpatient	End of life; end stage	Decision-making	Cross-sectional, Single centre	Interview	n/a	*N* = 25 (parent unspecified) Age at death: 3 mos – 12 yrs
Tenniglo et al. (2017)[97] Netherlands	2	Cancers	Inpatient, Outpatient	Treat/ manage	Decision-making	Cross-sectional, Multi-centre (*n* = 2)	Focus group	*N* = 11 (*n* = 6 boys, *n* = 5 girls) 12 – 18 yrs	*N* = 18 (*n* = 9 mothers, *n* = 9 fathers) 0 – 18 yrs
Thienprayoon et al. (2016)[98] USA	1,2	Cancers	Inpatient	End of life; end stage	No	Cross-sectional, Single centre	Interview	n/a	*N* = 34 (*n* = 18 mothers, *n* = 13 fathers, *n* = 3 ‘other family caregiverr’) 2 – 18 yrs
Tong et al. (2010)[99] Australia	1,2	Renal disease	Inpatient, Outpatient	Diagnosis; treat/ manage	No	Cross-sectional, Multi-centre (*n* = 2)	Interview	n/a	*N* = 20 (*n* = 15 mothers, *n* = 5 fathers) 0 – 18 yrs
Wangmo et al. (2016)[100] Switzerland	1,2	Cancers	Inpatient, Outpatient	Diagnosis; treat/ manage	No	Cross-sectional, Single centre	Interview	*N* = 17 (*n* = 11 boys, *n* = 6 girls) 9 – 17 yrs	*N* = 19 (*n* = 15 mothers, *n* = 4 fathers) Child age: not reported
Watt et al. (2011)[101] USA	1,2	Cancers	Inpatient, Outpatient	Diagnosis, treat/ manage	No	Cross-sectional, Multi-centre (no. of sites not reported)	Interview	n/a	*N* = 50 (*n* = 37 mothers, *n* = 13 fathers) Child age: not reported
Weidner et al. (2011)[102] USA	1,2	Mixed LT/LSCs	Inpatient	End of life	No	Cross-sectional, Single centre	Interview, focus group	n/a	*N* = 29 (*n* = 20 mothers, *n* = 9 fathers Child age: not reported
Young et al. (2011, 2013)[103, 104] UK	1,2	Cancers	Inpatient, Outpatient	Treat/ manage	Decision-making	Longitudinal Multi-centre (*n* = 6)	Interview	n/a	(2011) *N* = 53 (*n* = 33 mothers, *n* = 20 fathers) (2013) *N* = 67 (*n* = 40 mothers, *n* = 27 fathers) 1–12 yrs
Yuen et al. (2012)[105] Netherlands	1,2	Lethal Epi. Bullosa (LAEB)	Inpatient, Outpatient	Diagnosis; end stage	No	Cross-sectional, Single centre	Interview	n/a	*N* = 16 (parent unspecified) 0.1 – 32.6 mos
Zitzelsberger et al. (2014)[106] Canada	1	Renal disease	Inpatient	Treat /manage	Environment	Cross-sectional, Single centre	Interviews	*N* = 11 (*n* = 6 boys, *n* = 5 girls) 7 – 17 yrs	n/a

### Review 1: The hospital healthcare experiences of children and young people with life-threatening or life-shortening conditions

#### Characteristics of included studies

A total of 53 studies reported data on CYP’s experiences of hospital healthcare, either self-reported or via proxy reporting from parents, see Table 3 (additional details in Supplementary File 6). Nine studies recruited only CYP, 8 recruited CYP and parents, and 36 recruited only parents. This represents a total of 271 CYP (*n* = 142 boys, *n* = 129 girls) and 1,202 parents (*n* = 743 mothers, *n* = 302 fathers, 7 ‘family caregivers/legal guardians’, *n* = 150 not specified).

CYP study participants were aged 3–18 years, with one study failing to report the age range of CYP involved. For the studies which only recruited parents as proxy informants, the age range of their children was reported in 31/44 studies, with CYP aged 0–18 years represented. Participant ethnicity was reported by 24/53 studies but there was considerable variability in how it was categorised. Overall, the great majority of study participants were described as white or Caucasian. Only two studies investigated the impact of ethnicity on CYP’s healthcare experiences.

With respect to study design, almost all studies were cross-sectional (*n* = 48) and used interviews to collect data (*n* = 49). Other methods included focus groups (*n* = 6), and qualitative survey (*n* = 1). Over half (*n* = 36) were single site studies. Where studies recruited CYP, the CYP sample size ranged from 5 to 50 (median 13), and for studies recruiting parents, the parent sample size ranged from 5 to 67 (median = 21).

Over half (*n* = 33) the studies focused on a single diagnosis/diagnostic group, most frequently cancer (*n* = 22), followed by cerebral palsy (*n* = 2) and renal disease (*n* = 2), and with seven concerned with the following other diagnoses: cardiac disease; Duchenne muscular dystrophy; hydrocephalus; lethal acantholytic epidermolysis bullosa (LAEB); neutropenia; spinal muscular atrophy (type 1); and traumatic brain injury. The remaining studies (*n*- = 20) recruited samples with a number of different LT/LSCs: none compared differences in experience according to condition.

Studies covered all the stages of condition trajectory, from the period following diagnosis, through to treatment and management, critical episodes, end of life and end stage/death, with thirty focussed on a specific stage. The majority (*n* = 46) reported on inpatient experiences, of which 13 concerned experiences in intensive care. Just under half (*n* = 24) reported on outpatient experiences, with 7 studies only investigating this setting. Where the studies focused on a particular aspect of the healthcare experience (*n* = 29/53), this was most commonly decision making (*n* = 10), followed by relationships with staff (*n* = 5) and communication (*n* = 4). Just 2 studies investigated the impact of age on healthcare experience.

Twenty-three studies were North American, 7 were conducted in Australia and the same number in the UK. The remaining studies (*n* = 17) were conducted in other European countries (*n* = 15) and Mexico (*n* = 1).

#### Elements of health service delivery and care that matter to children and young people

The review identified 36 discrete aspects (or elements) of health service delivery and care that matter to CYP with a LT/LSC. These can be organised into a conceptual framework comprising 8 broad domains: two concerned with staff attributes; three concerned with staff actions; and three concerned with organisational features, see Table 4. One of these domains (‘Physical and sensory environment during inpatient stays’) is specific to inpatient hospital stays. In an additional two domains (‘Meeting emotional and social needs’ and ‘Resources for socialising and play’), there are some elements which were only reported in studies of inpatient hospital stays (e.g. ‘Staff ensure that the CYP has time alone when they want it’, ‘There is access to technology so that CYP can stay in contact with friends outside’ etc.).

**Table 4:** Elements of hospital health service delivery and care that matter to children and young people with a life-threatening or life-shortening condition: a conceptual framework

**STAFF ATTRIBUTES: Empathetic qualities**
• Staff are kind (*n* = 10)
• Staff are encouraging (*n* = 2)
**STAFF ATTRIBUTES: Professionalism**
• Staff are knowledgeable and skilled in managing the CYP's condition (*n* = 15)
• Staff are thorough and careful (*n* = 7)
• Staff are calm (*n* = 3) (PO)
**STAFF ACTIONS: Sharing medical information and decision making**
• Staff offer the CYP a choice as to how much information they are given about their health, treatment & care (*n* = 8)
• Staff explain medical information using words that the CYP understands (*n* = 8)
• Staff offer the CYP a choice about involvement in discussions about decisions which may affect their health (*n* = 5)
• Staff provide medical information in a caring way (*n* = 3)
• Staff pace the provision of medical information to meet the CYP’s needs (*n* = 2)
**STAFF ACTIONS: Delivering clinical and personal care**
• Staff minimise the CYP’s pain and discomfort (*n* = 12)
• Staff notice and respond to the CYP's requests for help (*n* = 9)
• Staff explain what is going to happen to the CYP (*n* = 9)
• Staff consult the CYP about how they want clinical procedures and care tasks to be carried out (*n* = 7)
• Staff ensure treatments and medications are provided to the CYP on time (*n* = 3)
• Staff notice and respond to the CYP’s non-verbal signals that they need attention (*n* = 3) (PO)
• Staff make sure the CYP is clean (*n* = 3) (PO)
• Staff are prepared for the CYP's admission to or attendance on the unit/ward (*n* = 2) (CYPO)
• Staff look after or help with the CYP’s appearance (*n* = 2) (PO)
**STAFF ACTIONS: Meeting emotional and social needs**
• Staff take time to get to know the CYP (e.g. their interests, life outside hospital etc.) (*n* = 25)
• Staff take the CYP’s whole life into account when arranging medical treatment and care (*n* = 3)
• Staff help the CYP to access toys, games, and other sources of entertainment (*n* = 2)
• Staff do all they can to ensure the CYP is calm and free from anxiety (*n* = 1) (PO)
• Staff ensure that the CYPs has time alone when they want it (*n* = 1) (CYPO) (Inpatient only)
**ORGANISATIONAL FEATURES: Resources for socialising and play**
• There is a supply of games, toys and other sources of entertainment suitable for the CYP (*n* = 3) (CYPO)
• There is access to technology so that the CYP can stay in contact with friends outside hospital (*n* = 1) (Inpatient only)
**ORGANISATIONAL FEATURES: Physical and sensory environment during inpatient stays**
• The layout of the ward allows the CYP to spend time with other CYP (*n* = 3) (CYPO)
• There are facilities so that parents can stay overnight (*n* = 3)
• The room/ward is not too noisy (*n* = 2)
• The room/ward is a comfortable temperature (*n* = 1)(CYPO)
• The beds are comfortable (*n* = 1) (CYPO)
• Ward facilities are accessible if using medical equipment or a wheelchair (*n* = 1)
• There are single rooms available for the CYP (*n* = 1) (PO)
• There is sufficient storage for the CYP’s property (*n* = 1) (CYPO)
• The food is appetising (*n* = 1)
**ORGANISATIONAL FEATURES: Continuity of care**
• Staff are familiar to the CYP (*n* = 12)

Figures in brackets indicate the number of papers reporting that the experience matters to children and young people

PO = Element reported as mattering by parents only

CYPO = Element reported as mattering by CYP only

There was considerable variation in the number of studies reporting each of the elements as mattering to CYP, see also Table 4. The element most frequently reported as mattering to CYP was ‘Staff take time to get to know the CYP’ (*n* = 25/53), followed by ‘Staff are knowledgeable and skilled in managing the CYP’s condition’ (*n* = 15/53). All other elements were reported by ten studies or fewer. Whilst most elements (*n* = 24) were identified as mattering to CYP by both CYP and parents, some were only described by CYP (*n* = 7), and others only by parents (*n* = 6), see also Table 4.

#### The impact of health service delivery and care on children and young people

Most of the included studies in which CYP were study participants (*n* = 15/17) reported CYP’s descriptions of how health service delivery and care affected them. Impacts were wide-ranging with seven areas of impact identified: emotional well-being (e.g. frustration, panic, shock, sadness), physical well-being (e.g. pain, sleeplessness), trust in staff, how comfortable CYP felt with staff, understanding of the situation being faced, sense of empowerment and control, and being at ease with (versus regretting) treatment decisions. For further details on the nature of these impacts see Supplementary File 4. 

Table 5 presents the results of mapping out connections between elements of health service delivery and care (grouped into the 8 domains described above) and impact(s). All domains of health service delivery and care were identified as impacting emotional wellbeing. Domains having the widest range of impacts on CYP’s lives were staff actions with respect to sharing of medical information and decision-making, and the delivery of clinical and personal care.

**Table 5:** Impacts of health service delivery and care on children and young people

	**Impact on children and young people**						
**Domain of health service delivery & care**	Emotional wellbeing	Physical wellbeing	Trust in staff	Feeling (un) comfortable with staff	Understanding of situation being faced	Empowerment & control	At ease with (versus regretting) treatment decisions
STAFF ACTIONS							
Sharing medical information & decision making	✓		✓	✓	✓	✓	✓
Delivering clinical & personal care	✓	✓	✓		✓	✓	
Meeting emotional & social needs	✓			✓			
ORGANISATIONAL FEATURES							
Resources for socialising & play	✓						
Physical & sensory environment during inpatient stays	✓	✓					
Continuity of care	✓		✓				

The findings of this review, and resultant conceptual framework, were shared with the Family Advisory Board. Parents endorsed the conceptual framework, reporting either that they were aware of these elements mattering to their CYP, or would not be surprised if they did.

### Review 2: The hospital healthcare experiences of parents of a child/young person with a life-threatening or life-shortening condition

#### Characteristics of included studies

Sixty-four studies reported on parents’ experiences of hospital healthcare in their parenting role, representing a total of 1,892 parents (*n* = 948 mothers, *n* = 436 fathers, 7 family caregivers/legal guardians, *n* = 501 not specified), see Table 3 (additional details in Supplementary File 6). In 45/64 studies the age of their child with a LT/LSC was reported, with children aged 0–18 years represented. Parents’ ethnicity was reported by 30/64 studies, and in these a large majority were described as white or Caucasian. Only two studies investigated the impact of ethnicity on parents’ healthcare experiences.

The majority of studies were cross-sectional (*n* = 59), with most using interviews to collect data (*n* = 58). Other methods used included focus groups (*n* = 9), qualitative surveys (*n* = 2). Most (*n* = 42) were single site studies. The sample size ranged from 5 to 266 (median = 19).

Over half (*n* = 35) the studies concerned a single diagnosis/diagnostic group, most frequently cancer (*n* = 21), followed by cerebral palsy (*n *= 3). Other conditions represented in single diagnosis studies (*n* = 11) were cardiac disease, cystic fibrosis, Duchenne muscular dystrophy, hydrocephalus, neutropenia, lethal epidermolysis bullosa, Pierre robin sequence, renal disease, SMA type 1 and traumatic brain injury. The remainder (*n* = 29) either recruited parents of CYP with a number of different LT/LSCs (*n* = 22), or the sample was defined in terms of the paediatric service or medical treatment required (e.g. paediatric palliative care, bone marrow transplant etc.) (*n* = 7). Overall, studies covered all stages in the condition trajectory, with 30 focussing on a specific stage. The majority of studies (*n* = 59) reported on inpatient experiences, of which 18 reported on experiences of intensive care. Twenty-five studies reported on outpatient experiences, with 5 investigating this healthcare context only. Where the studies focused on a particular aspect of the healthcare experience (*n* = 32/64), this was most commonly decision making (*n* = 14), followed by communication (*n* = 5). Only one study investigated the impact of the child’s age on parents’ experiences.

Twenty-seven studies were North American, 11 were conducted in the UK, 7 in Australia and 1 in Mexico. The remainder (*n* = 18) were conducted in other European countries.

#### Elements of health service delivery and care that matter to parents

The review identified 55 elements of health service delivery and care that matter to parents who have a CYP with a LT/LSC. These can be organised into 9 broad domains: two concerned with staff attributes; four with staff actions; and three with organisational features, see Table 6. One of these domains is specific to situations in which the CYP is a hospital inpatient (‘Physical environment during hospital inpatient stays’). In an additional two domains (‘Supporting coping’, ‘Supporting parenting’), there are some elements only reported in situations in which the CYP was a hospital inpatient.

**Table 6:** Elements of hospital health service delivery and care that matter to parents of children with a life-threatening or life-shortening condition: a conceptual framework

**STAFF ATTRIBUTES: Empathetic qualities**
• Staff are kind (*n* = 7)
• Staff are patient (*n* = 4)
**STAFF ATTRIBUTES: Professionalism**
• Staff are honest (*n *= 24)
• Staff are knowledgeable and skilled in managing the CYP’s condition (n = 22)
• Staff are committed to caring for the child (*n* = 10)
• Staff are polite (*n* = 3)
• Staff are calm (*n* = 2)
• Staff are respectful of cultural and religious beliefs (*n* = 2)
**STAFF ACTIONS: Sharing medical information and decision making with parents**
• Staff involve the parent to the extent that they want in decisions about treatment and care (*n* = 26)
• Staff explain things in ways the parent understands (*n* = 23)
• Staff give difficult or bad news sensitively (*n *= 22)
• Staff give the parent all the information they want about the child’s condition, treatment and care (*n* = 18)
• Staff are willing to answer questions (*n* = 13)
• Staff pace the provision of medical information according to the parent’s readiness and capacity for information at the time (*n* = 12)
• Staff make themselves available to talk to the parent (*n* = 12)
• Staff keep the parent updated on changes in their child’s condition, treatment and care (*n* = 10)
• Staff give difficult or bad news in private (*n* = 4)
• Staff give the parent time to think about significant decisions (*n* = 4)
• Staff check with the parent about discussing medical information in front of their child (*n* = 3)
• Staff check with the parent about how much they tell their child (*n* = 1)
**STAFF ACTIONS: Management of the child’s condition**
• Staff listen to and respect the parent’s views on their child’s condition, treatment and care (*n* = 24)
• Staff give the child the same level of care and attention as other children (n = 5)
• Staff on the ward/unit communicate with each other about the CYP’s treatment and care (*n* = 4)
• Staff agree about the CYP’s treatment and care (*n* = 3)
• Staff take information and advice from other specialisms into account when deciding on treatment and care (*n* = 3)
• Staff take family circumstances into account when arranging treatment and care (*n* = 2)
• Staff are willing to be questioned about the child’s treatment and care (*n* = 1)
**STAFF ACTIONS: Supporting coping**
• Staff acknowledge the impact of the situation on the parent (*n* = 13)
• Staff allow the parent to be hopeful (*n* = 11)
• Staff take time to talk to and get to know the parent as an individual (*n* = 10)
• Staff ask the parent how they are feeling (*n* = 6)
• Staff prepare the parent for any changes they might see in their child (*n* = 6)
• Staff give the parent information on hospital facilities (e.g. where to get food, washed) (*n* = 5) (Inpatient only)
• Staff encourage the parent to take care of themselves (e.g. rest, eat etc.) (*n *= 4) (Inpatient only)
• Staff comfort the parent (*n* = 3)
• Staff allow the parent to be on the ward as much as is possible (*n* = 3) (Inpatient only)
• Staff offer to introduce the parents to other parents on the unit/ward (*n* = 3) (Inpatient only)
• Staff talk to the parent about life outside the hospital (*n *= 2) (Inpatient only)
**STAFF ACTIONS: Supporting parenting**
•Staff support the parent to care for their child as much as they would like to (e.g. changing clothes, washing, feeding etc.) (*n *= 13) (Inpatient only)
• Staff make sure the parent can be physically close to and/or hold their child (*n* = 7)
• Staff support the parent to take on any medical responsibilities they want to be involved in (*n* = 6) (Inpatient only)
• Staff do their best to ensure the parent has as much time with their child as they want (*n* = 5) (Inpatient only)
• Staff support the parent with talking to siblings about the child’s health problems (*n* = 3)
• Staff ensure the parent has opportunities for privacy with their child (*n* = 1) (Inpatient only)
• Staff support the parent with explaining difficult or bad news to their child (*n* = 1)
• Staff give the parent information on how the ward/unit operates (e.g. staff roles, shift patterns, visiting hours etc.)(*n* = 1)
**ORGANISATIONAL FEATURES: Physical environment during inpatient stays**
• There are toilets on the ward for the parent (*n *= 2)
• There is a room for the parent to use when they need a break (*n *= 2)
• The layout of the room allows the parent to have time alone with their child (*n* = 2)
**ORGANISATIONAL FEATURES: Continuity and coordination of care**
• Staff are familiar to the parent (*n* = 18)
• There is a staff member responsible for coordinating treatment and care (*n* = 10)
**ORGANISATIONAL FEATURES: Access to additional support**
• Psychological support services are available to the parent (*n* = 9)
• Spiritual care services are available to the parent (e.g. chaplains, faith leaders etc.) (*n* = 4)
• Information on welfare/benefits advice is available to the parent (*n* = 1)
• Interpreters are available to the parent (*n* = 1)

Figures in brackets indicate the number of papers reporting that the experience matters to parents

The elements of health service delivery and care most frequently reported as mattering to parents (*n* > 20 studies reporting) were: ‘Staff are knowledgeable and skilled in managing the CYP’s condition’; ‘Staff are honest with parents’; ‘Staff involve parents to the extent that they want when significant decisions are being made’; ‘Staff give difficult or bad news sensitively’; ‘Staff explain things in ways the parent understands’; and ‘Staff listen to and respect parent’s views about their child’s condition and care’.

Two further elements of health service delivery and care which some parents had reported as valuing were identified in 8 studies. Both staff actions, these elements had been labelled: ‘staff behaving in ways that indicated high levels of emotional distress about child’s/family’s situation’ (e.g. openly weeping), and ‘staff making themselves available to a family beyond usual or expected practice’ (e.g. going into work on a day off to see a family; flying home from holiday early to be with a family at end of life). The research team discussed the inclusion of these elements in the conceptual framework with the project’s clinical/academic advisory group. With respect to the first element (‘Staff behaving in ways that indicated high levels ….’), the advisory group agreed that the data coded under this element was different to that coded under other staff attributes or actions which captured empathetic care. They also agreed that, whilst conveying distress at the suffering/situations patients/families face is natural and appropriate, there are limits to this and uncontrolled displays of emotion are not professional.

In terms of the second element (‘Staff making themselves available…..’), the advisory group agreed that such actions/behaviours could not be expected to implemented into routine service provision. In addition, it potentially indicated a favouring of particular patients/families: something directly in conflict to principles of equity of care (and something which the project’s parent advisory group had identified as very important). It was therefore agreed that the conceptual framework should not include these two elements.

#### The impacts of health service delivery and care on parents

Most included studies (*n* = 61/64) described how these elements of health service delivery and care affected parents. Together, they indicate that multiple areas of parents’ lives can be affected by health service delivery and care, including (and most commonly) emotional well-being (e.g. anxiety, anger, fear etc.), being able to parent in the way they want and parent’s trust in staff, see Table 7. For further details on the nature of these impacts see Supplementary File 5.

**Table 7:** Impacts of health service delivery and care on parents

	**Impact on parents**											
**Domain of health service delivery & care**	Emotional wellbeing	Ability to parent child in way they want	Trust in staff	Care burden	Empowerment & control	Understanding of situation being faced	Partnership (versus conflict) with staff	Maintenance of usual family routine	At ease with (versus regretting) treatment & care decisions	Satisfaction with treatment & care	Sense of hope	Physical wellbeing
STAFF ACTIONS												
Sharing medical information & decision making	✓	✓	✓	✓	✓	✓	✓		✓	✓	✓	
Management of child’s condition	✓	✓	✓	✓	✓	✓	✓	✓				
Supporting coping	✓	✓	✓				✓			✓	✓	
Supporting parenting during inpatient stays	✓	✓	✓	✓	✓			✓	✓			
ORGANISATIONAL FEATURES												
Physical environment during inpatient stays	✓	✓						✓				✓
Continuity & coordination of care	✓	✓	✓	✓								
Access to additional support	✓	✓		✓								

Table 7 also presents findings from the analysis which mapped connections between the specific elements of health service delivery and care (grouped into the 9 domains set out in Table 6) and the various potential impacts on parents’ lives. All domains of service delivery and care were found to be reported as impacting on emotional wellbeing, and almost all domains (7/8) on being able to parent in the way they want. The domains having the widest range of impacts on parents’ lives are staff actions, both in terms of sharing medical information and decision making, and management of the child’s condition. This is closely followed by supporting parenting during impatient stays and supporting coping.

## Discussion

This paper reports parallel scoping reviews of qualitative evidence on the health service experiences of CYP with LT/LSC and their parents, and the resultant conceptual frameworks, setting out the elements of hospital (inpatient and outpatient) health service delivery and care that matter to, and impact on, CYP with a LT/LSC and their parents. Both frameworks are comprised of elements of hospital health service delivery and care (36 for CYP, 55 for parents) which are conceptually distinct. The generation of these conceptual frameworks is the first stage in the development of a CYP PREM and PaREM which, it is intended, can be used to assess both experiences of specific episodes of care (e.g. inpatient admission) and longer-term periods of care under a particular service or specialism. While some elements are specific to inpatient experiences only, none are specific to a particular staff group, stage in the illness trajectory or child’s age/developmental stage. This fits with recent calls for paediatric measures to be developed which are applicable to the multiple service providers in a child’s network of care and across the child’s illness experience [17].

Compared to many existing patient experience frameworks, the frameworks we have developed are deliberately organised around a number of domains of care (e.g. clinical and personal care, the physical and sensory environment etc.), rather than cross-cutting concepts (e.g. respect for patient values and preferences, physical wellbeing), something critiqued by other researcher as limiting the way PREMs can inform service evaluation and service improvement activities, and allow meaningful comparisons by research studies [10, 11].

Similar to previous work [10, 11], the scoping reviews have identified specific staff attributes, and actions or behaviours, that matter to and impact CYP’s and parents’ health service experience, alongside physical and sensory features, and the availability of various facilities and sources of support. To our knowledge this is the first conceptual framework of patient experience developed for CYP with a LT/LSC, and the first conceptual framework of parent experience (across any paediatric population) which is grounded in parents’ views and accounts.

It is recognized globally that, relative to adult patients, attempts to collect CYP’s experiences and views on healthcare provision is very limited [107–110]. This is a critical gap given parent-proxy reporting does not accurately reflect the experiences of CYP, with parents tending to rate experiences more positively than their child [111–113]. Furthermore, our own review highlights differences between CYP and parents in the aspects of the CYP’s healthcare which matter to them. Specifically, findings from direct research with CYP reveals the importance of multiple aspects of the physical and sensory environment, resources for socializing and play, and meeting social and emotional needs to CYP (e.g. ‘the layout of the ward allows the CYP to spend time with other CYP’, ‘the bed are comfortable’, ‘staff ensure the CYP has time alone when they want it’ etc.) which do not emerge from research with parents on their child’s healthcare experiences. These findings reinforce the importance of researchers exercising caution in relying on proxy data.

In addition, we have argued for the need to assess both CYP *and* parent experience given that a parent’s wellbeing and associated ability to parent is highly likely to impact their child [114–117]. It is notable that key domains identified as important by parents themselves include supporting parenting whilst the child is an inpatient, and supporting coping with the emotional impact and practical implications of their child’s condition and hospital admission.

Finally, a novel and important aspect of both scoping reviews is the identification of the way experiences of using health services impact on multiple areas of CYP’s and parents’ lives. These impacts included: emotional and physical wellbeing; trust in and relationships with staff; understanding of the situation being faced; and satisfaction with treatment, care and decision-making. Importantly, for each type of impact, multiple and diverse elements of health service delivery and care were implicated. This raises questions about the utility of patient experience frameworks (and associated PREMs) which solely focus on measuring impacts, given that they do not yield information about what has caused these experiences.

### Limitations

Despite an inclusive search strategy, just 4 studies were identified which reported on experiences of using community health services. Given this very small number, and that a reading of the articles revealed that healthcare experiences reported were quite different to those reported in hospital based studies, a decision was taken to limit the scoping reviews, and therefore the resultant conceptual frameworks, to hospital healthcare experiences. The limited body of research on experiences of community-based health care for CYP with LT/LSCs, and their parents, is concerning and highlights the need for further research on this topic.

Across the two reviews that were undertaken, a range of different types of LT/LSCs were represented. However, a high proportion of studies were concerned with CYP with cancer (21/53 in Review 1; 21/64 in Review 2). The relative lack of evidence on CYP with diagnoses requiring the involvement of multiple specialisms, or which carry implications in terms of cognitive and physical abilities, may mean not all elements of healthcare mattering to CYP with LT/LSC and their parents have been identified and described by existing research. Additional research is needed to investigate this further. In both conceptual frameworks the number of studies identifying each element of health delivery and care are reported (see Tables 4 and 6). This information gives an indication of the depth of data on a particular element. However, these figures should not be taken as an indication of the relative importance of elements since over half the studies in Review 1 and 2 focused on a specific aspect of health service delivery and care (e.g. decision making, communication etc.). Finally, requiring a sample size of 5 or more could have led to us excluding studies taking an Interpretative Phenomenological Analysis (IPA) approach. However, no studies were excluded solely on the basis of sample size. We also note that, whilst there is no firm consensus around minimum sample sizes for IPA, sample sizes of *n* > 6–8 are generally encouraged for studies not being undertaken for educational (undergraduate/postgraduate studies) purposes.

As noted in the results, across both reviews the impact of ethnicity on what matters to CYP was poorly investigated, with just over half the papers reporting participants’ ethnicity, and only 2 studies investigating the issue. Where ethnicity was reported, it was often inadequately described, with the majority of participants said to be White or Caucasian, with no further information on their ethnic or cultural background provided. We therefore currently have no evidence as to whether there are ethnic differences in the elements of health delivery and care that matter to CYP or parents. We must also be mindful of the fact that the research reviewed was undertaken in OECD countries; it cannot be assumed that the frameworks apply to other settings.

In relation to Review 1, just 17/53 of the included studies recruited CYP. Whilst recruiting parents to research on the experiences of CYP with LT/LSCs is necessary given that some CYP may be limited in their ability to participate due to cognitive or communication difficulties or feeling too unwell, this does mean that we need to treat the emergent conceptual framework as, potentially, incomplete. We also note that the CYP’s framework, reflecting the themes that emerged from Scoping Review 1, is relatively thin with regards to the provision of emotional support, with no direct reference to healthcare professionals offering CYP opportunities to talk about worries and fears. This is somewhat surprising given that previous research with CYP with LT/LSCs has found they have psychological and psychosocial concerns, and research prioritization exercises involving CYP suggest emotional and spiritual support is a priority [118, 119]. However, undertaking qualitative research with CYP is challenging [120, 121] and it is entirely possible that the lack of data on this issue reflects difficulties experienced by researchers in interviewing research participants about the more distressing aspects of living with a LT/LSC. Future research will need to address this issue, albeit sensitively. Finally, most studies recruited CYP across a very broad range of ages and just two investigated age differences in experience. We were therefore unable to draw unable conclusions as to whether the elements of health service delivery and care that matter differ according to age and/or developmental stage of the CYP.

One of the strengths of these reviews is that the research team consulted both a family and a professional advisory group about the review design, preliminary findings, and the resultant conceptual fameworks. It also provided an initial validation of the CYP’s conceptual framework. Further validation work for both frameworks, including very importantly children and young people, will be an essential next step in the development of the PREM, as well as the PaREM. In undertaking this work, special attention will need to be paid to ensuring a range of diagnoses (and particularly those under-represented in existing studies), CYP age groups, disease stages and ethnic/cultural groups are represented.

## Conclusions

Following a comprehensive and rigorous review of the qualitative research on the health care experiences of CYP with a LT/LSC and their parents, two conceptual frameworks have been developed which delineate the elements of health service delivery and care that matter to CYP with a LT/LSC as inpatients and outpatients, and their parents. In addition, the impact of attending (or not) to these elements of care have been mapped out. The findings make it clear that while there are some overlaps between what matters to CYP and parents, they also have their own distinct and specific needs. Going forward, these frameworks will be used to develop measures of healthcare experience for CYP and parents for use in service evaluation, service improvement projects and research.

## Supplementary Information

**Additional file 1: Supplementary File 1.** Literature review search strategies.

**Additional file 2: SupplementaryFile 2.** Final versions of data extraction frameworks.

**Additional file 3: Supplementary File 3.** Extract from a thematic table used for main analysis.

**Additional file 4: Supplementary File 4.** The impacts of health service delivery and care on children and young people - List of codes and their definitions.

**Additional file 5: Supplementary File 5.** The impacts of health service delivery and care on parents- List of codes and their definitions.

**Additional file 6: Supplementary File 6.** Additional study characteristics.

## Data Availability

The datasets used and/or analysed during the current study are available from the corresponding author on reasonable request.

## Abbreviations

CYP: Children and young people
LT/LSC: Life-threatening or life-shortening condition
PREM: Patient-reported experience measure
PaREM: Parent-reported experience measure
