# Supplementary material for: Hospital healthcare experiences of children and young people with life-threatening or life-shortening conditions, and their parents: scoping reviews and resultant conceptual frameworks
Source: BMC Pediatr. 2023 Jul 17;23:366. doi: 10.1186/s12887-023-04151-6 (PMC10351142; doi:10.1186/s12887-023-04151-6)
Supplement: Supplementary file 1 — Additional file 1: Supplementary File 1. Literature review search strategies. [file 12887_2023_4151_MOESM1_ESM.docx]

**Supplementary File 1:**

**Literature review search strategies**

Three databases were searched: MEDLINE, PsycINFO, and CINAHL Complete,

The search strategies are presented below

**MEDLINE via OVID**

Search date 11^th^ August 2020

Database: Ovid MEDLINE(R) ALL <1946 to August 10, 2020>

**Search Strategy**

1 Creutzfeldt-Jakob Syndrome/ (6397)

2 (creutzfeldt-jakob$ or jakob-creutzfeldt$ or cjd or spongiform encephalopath$).ti,ab,kf. (10520)

3 Subacute Sclerosing Panencephalitis/ (2299)

4 (subacute sclerosing panencephalit$ or sub-acute sclerosing panencephalit$ or sspe or subacute sclerosing leukoencephalit$ or sub-acute sclerosing leukoencephalit$ or van bogaert$ leukoencephalit$ or measles inclusion body encephalit$ or mibe).ti,ab,kf. (2389)

5 beta-Thalassemia/ (8764)

6 (beta adj (thalass?emi$ or thalas?emi$)).ti,ab,kf. (11102)

7 ((thalass?emi$ or thalas?emi$) adj major).ti,ab,kf. (4724)

8 exp Anemia, Aplastic/ (16634)

9 ((hypoplastic or aplastic) adj an?emi$).ti,ab,kf. (10566)

10 (medullary adj3 hypoplas$).ti,ab,kf. (51)

11 exp Neutropenia/ (18684)

12 ((severe or chronic$) adj3 neutropeni$).ti,ab,kf. (3635)

13 immunologic deficiency syndromes/ or acquired immunodeficiency syndrome/ (90862)

14 (immun$ deficiency adj (syndrome$ or disease$ or disorder$)).ti,ab,kf. (6927)

15 (immunodeficiency adj (syndrome$ or disease$ or disorder$)).ti,ab,kf. (24632)

16 DiGeorge Syndrome/ (2148)

17 (digeorge$ or di george$ or sedlackova$ or opitz g-bbb or velocardiofacial or velo-cardiofacial or velo-cardio-facial or shprintzen$ or ctaf).ti,ab,kf. (2665)

18 ((deletion or vcf or pharyngeal pouch or thymic aplasia or anomaly face) adj (syndrome$ or disease$ or disorder$)).ti,ab,kf. (3044)

19 Common Variable Immunodeficiency/ (2162)

20 ((common variable or late onset) adj3 (immunodeficienc$ or immune deficienc$ or immunoglobulin deficienc$ or hypogammaglobulin$)).ti,ab,kf. (3003)

21 acquired hypogammaglobulin$.ti,ab,kf. (136)

22 Cryoglobulinemia/ (3152)

23 cryoglobulin?em$.ti,ab,kf. (4382)

24 Polyendocrinopathies, Autoimmune/ (1246)

25 ((autoimmune or failure$) adj3 (polyglandular$ or polyendocrin$)).ti,ab,kf. (1528)

26 Progeria/ (1516)

27 (progeria or hutchinson-gilford$).ti,ab,kf. (1632)

28 Tyrosinemias/ (434)

29 Maple Syrup Urine Disease/ (1119)

30 tyrosin?em$.ti,ab,kf. (1228)

31 (maple syrup urine or msud).ti,ab,kf. (1184)

32 branched chain.ti,ab,kf. (10319)

33 (bckd adj5 (deficienc$ or ketoacid$ or keto-acid$)).ti,ab,kf. (88)

34 hyperleucine-isoleucin$.ti,ab,kf. (1)

35 Methylmalonic Acid/ (1589)

36 (methylmalonic acid?emi$ or methylmalonic aciduri$ or methyl malonic acid?emi$ or methyl malonic aciduri$).ti,ab,kf. (1342)

37 Propionic Acidemia/ (192)

38 (propionic acid?em$ or propionic acidur$ or propionyl-CoA carboxylase deficienc$ or ketotic glycin?em$).ti,ab,kf. (757)

39 Adrenoleukodystrophy/ (1728)

40 (adrenoleukodystroph$ or x-ald or schilder-addison$ or addison-schilder$ or adrenomyeloneuropath$).ti,ab,kf. (2194)

41 Carnitine O-Palmitoyltransferase/ (2531)

42 ((carnitine palmityltransferase or carnitine palmitoyltransferase or carnitine o-palmityltransferase or carnitine o-palmitoyltransferase) adj3 deficienc$).ti,ab,kf. (294)

43 Fanconi Syndrome/ (1624)

44 (fanconi$ adj (syndrome$ or disease$ or disorder$)).ti,ab,kf. (1788)

45 (ocular adj3 (renal or kidney)).ti,ab,kf. (447)

46 Cystinosis/ (1229)

47 (cystinos$ or cystine storage or cystine diathes$ or cystine disease$).ti,ab,kf. (1347)

48 Oculocerebrorenal Syndrome/ (404)

49 ((lowe or lowes or oculocerebrorenal or cerebrooculorenal or cerebro-oculo-renal) adj3 (syndrome$ or disease$ or disorder$)).ti,ab,kf. (578)

50 Metalloproteins/df [Deficiency] (54)

51 Molybdenum/df [Deficiency] (65)

52 (molybdenum cofactor deficien$ or molybdenum co-factor deficien$).ti,ab,kf. (171)

53 Oxidoreductases Acting on Sulfur Group Donors/df [Deficiency] (98)

54 Sulfite Oxidase/df [Deficiency] (44)

55 ((sulphite$ or sulfite$) adj3 oxidase deficien$).ti,ab,kf. (156)

56 Argininosuccinic Acid/ (170)

57 (argininosuccinic acidur$ or argininosuccinic acid?emi$).ti,ab,kf. (178)

58 Citrullinemia/ (295)

59 (citrullin?emi$ or citrullinuri$).ti,ab,kf. (572)

60 Amino Acid Metabolism, Inborn Errors/ (5919)

61 (glutaric acid?emi$ or glutaric aciduri$).ti,ab,kf. (815)

62 Hyperglycinemia, Nonketotic/ (222)

63 (glycine encephalopath$ or non-ketotic hyperglycin?emi$ or nonketotic hyperglycin?emi$).ti,ab,kf. (523)

64 Hyperargininemia/ (190)

65 (arginin?emi$ or arginase deficien$ or hyperarginin?emi$).ti,ab,kf. (299)

66 Renal Aminoacidurias/ (744)

67 (aminoaciduri$ or aminoacid?emi$).ti,ab,kf. (1153)

68 exp glycogen storage disease/ (6135)

69 (glycogen storage adj (disease$ or syndrome$ or disorder$)).ti,ab,kf. (2958)

70 (pompe$ adj (disease$ or syndrome$ or disorder$)).ti,ab,kf. (1710)

71 Galactosemias/ (2269)

72 galactos?emi$.ti,ab,kf. (2191)

73 Pyruvate Dehydrogenase Complex Deficiency Disease/ (394)

74 (pyruvate dehydrogenase adj3 deficien$).ti,ab,kf. (443)

75 (oxalosis and (renal or kidney$)).ti,ab,kf. (460)

76 exp Gangliosidoses/ (2550)

77 gangliosidos$.ti,ab,kf. (1509)

78 (sandhoff$ adj (disease$ or syndrome$ or disorder$)).ti,ab,kf. (531)

79 tay sach$.ti,ab,kf. (1465)

80 Mucolipidoses/ (1053)

81 mucolipidos$.ti,ab,kf. (950)

82 Canavan Disease/ (292)

83 (canavan$ leucodystroph$ or aspartoacylase deficien$ or aminoacylase 2 deficien$).ti,ab,kf. (43)

84 ((canavan$ or canavan-van bogaert-bertrand$) adj (disease$ or syndrome$ or disorder$)).ti,ab,kf. (387)

85 Gaucher Disease/ (4608)

86 (gaucher$ adj (disease$ or syndrome$ or disorder$)).ti,ab,kf. (5001)

87 (glucocerebrosidase deficien$ or glucosylceramidase deficien$).ti,ab,kf. (115)

88 Leukodystrophy, Metachromatic/ (1227)

89 (metachromatic leukodystroph$ or arylsulfatase A deficien$ or metachromic leukodystroph$).ti,ab,kf. (1172)

90 exp Niemann-Pick Diseases/ (2499)

91 (niemann-pick$ or sphingomyelinase deficien$).ti,ab,kf. (3764)

92 Sphingolipidoses/ (364)

93 sphingolipidos$.ti,ab,kf. (346)

94 Fabry Disease/ (3527)

95 (fabry$ adj (disease$ or syndrome$ or disorder$)).ti,ab,kf. (4060)

96 (angiokeratoma corporis diffusum or alpha-galactosidase A deficien$).ti,ab,kf. (394)

97 Leukodystrophy, Globoid Cell/ (990)

98 (krabbe$ adj (disease$ or syndrome$ or disorder$)).ti,ab,kf. (927)

99 (globoid cell leukodystroph$ or galactosylceramide lipidos$ or galactosylcerebrosidase deficien$ or galactosylceramidase deficien$).ti,ab,kf. (494)

100 Farber Lipogranulomatosis/ (50)

101 (farber$ adj (disease$ or syndrome$ or disorder$)).ti,ab,kf. (191)

102 (farber$ lipogranulomatos$ or ceramidase deficien$ or fibrocytic dysmucopolysaccharidos$).ti,ab,kf. (75)

103 Pelizaeus-Merzbacher Disease/ (318)

104 pelizaeus-merzbacher$.ti,ab,kf. (618)

105 Sulfatases/df [Deficiency] (372)

106 Multiple Sulfatase Deficiency Disease/ (35)

107 (sulfatase deficien$ or sulphatase deficien$ or mucosulfatidos$).ti,ab,kf. (461)

108 (austin$ adj (disease$ or syndrome$ or disorder$)).ti,ab,kf. (5)

109 sulfatidos$.ti,ab,kf. (16)

110 Sea-Blue Histiocyte Syndrome/ (73)

111 sea-blue histiocyt$.ti,ab,kf. (226)

112 Neuronal Ceroid-Lipofuscinoses/ (1993)

113 (batten$ adj (disease$ or syndrome$ or disorder$)).ti,ab,kf. (736)

114 (neuronal ceroid lipofuscinos$ or santavuori-haltia$ or jansky-bielschowsky$ or bielschowsky-jansky$).ti,ab,kf. (1964)

115 (kuf$ adj (disease$ or syndrome$ or disorder$)).ti,ab,kf. (111)

116 spielmeyer vogt$.ti,ab,kf. (102)

117 Xanthomatosis, Cerebrotendinous/ (344)

118 ((cerebrotendineous or cerebrotendinous or cerebrotendious or cerebral) adj3 (xanthomatos$ or cholesteros$)).ti,ab,kf. (675)

119 bogaert-scherer-epstein$.ti,ab,kf. (6)

120 Wolman Disease/ (241)

121 (wolman$ adj (disease$ or syndrome$ or disorder$)).ti,ab,kf. (316)

122 lysosomal acid lipase deficien$.ti,ab,kf. (160)

123 exp Mucopolysaccharidoses/ (6438)

124 mucopolysaccharidos$.ti,ab,kf. (4723)

125 (hurler$ adj2 (syndrome$ or disease$ or disorder$)).ti,ab,kf. (980)

126 (hunter$ adj2 (syndrome$ or disease$ or disorder$)).ti,ab,kf. (981)

127 (MPS1 or MPS2 or MPS3 or MPS4 or MPS5 or MPS6 or MPS7 or MPS-1 or MPS-2 or MPS-3 or MPS-4 or MPS-5 or MPS-6 or MPS-7 or MPSI or MPSII or MPSIII or MPSIV or MPSV or MPSVI or MPSVII or MPS-I or MPS-II or MPS-III or MPS-IV or MPS-V or MPS-VI or MPS-VII).ti,ab,kf. (2700)

128 (beta glucuronidase deficien$ or sly syndrome$ or sly disorder$ or sly disease$).ti,ab,kf. (192)

129 (maroteaux-lamy$ or marotaeux-lamy$ or polydystrophic dwarfism).ti,ab,kf. (350)

130 (morquio$ or moriquio$ or beta galactosidase deficien$).ti,ab,kf. (1002)

131 (sanfilippo$ or sanfillipo$).ti,ab,kf. (652)

132 Mucolipidoses/ (1053)

133 (mucolipidos$ or pseudo-hurler$ or pseudohurler$).ti,ab,kf. (974)

134 ((inclusion-cell or i-cell) adj (disease$ or syndrome$ or disorder$)).ti,ab,kf. (392)

135 Fucosidosis/ (152)

136 (fucosidos$ or fucidos$).ti,ab,kf. (321)

137 "Congenital Disorders of Glycosylation"/ (680)

138 ((cdg or ctg) adj (disease$ or disorder$ or syndrome$)).ti,ab,kf. (92)

139 (carbohydrate-deficient glycoprotein adj (disease$ or disorder$ or syndrome$)).ti,ab,kf. (226)

140 (congenital disorder$ adj3 glycosylation).ti,ab,kf. (1018)

141 Lesch-Nyhan Syndrome/ (1208)

142 ((nyhan$ or kelley-seegmiller$) adj (syndrome$ or disorder$ or disease$)).ti,ab,kf. (988)

143 juvenile gout.ti,ab,kf. (27)

144 Menkes Kinky Hair Syndrome/ (997)

145 menkes$.ti,ab,kf. (1285)

146 ((copper transport or steely hair or kinky hair) adj (disease$ or syndrome$ or disorder$)).ti,ab,kf. (262)

147 alpha 1-Antitrypsin Deficiency/ (3414)

148 (antitrypsin deficien$ or A1AD).ti,ab,kf. (3320)

149 (AAT deficien$ or alpha-1 protease deficien$).ti,ab,kf. (536)

150 bisalbumin?emi$.ti,ab,kf. (190)

151 Lipodystrophy, Congenital Generalized/ (200)

152 (congenital generali?ed lipodystroph$ or berardinelli$ or bernardnelli$).ti,ab,kf. (419)

153 Landau-Kleffner Syndrome/ (280)

154 (landau-kleffner$ or infantile acquired aphasia$ or acquired epileptic aphasia$).ti,ab,kf. (470)

155 (aphasia$ adj5 convulsive).ti,ab,kf. (37)

156 Rett Syndrome/ (2585)

157 (rett$ adj (syndrome$ or disease$ or disorder$)).ti,ab,kf. (3561)

158 cerebroatrophic hyperammon?emi$.ti,ab,kf. (0)

159 Huntington Disease/ (12121)

160 huntington$.ti,ab,kf. (18057)

161 exp Spinocerebellar Ataxias/ (6354)

162 (spinocerebellar ataxia$ or ataxia$ telangiectasia$ or louis-bar$ syndrome$ or louis-bar$ disease$ or louis-bar$ disorder$ or machado-joseph$ or joseph$ disease$ or joseph$ disorder$ or joseph$ syndrome$).ti,ab,kf. (11478)

163 Friedreich Ataxia/ (2545)

164 ((friedreich$ or friedrich$) adj3 ataxia$).ti,ab,kf. (2958)

165 spinocerebellar degenerat$.ti,ab,kf. (708)

166 "Spinal Muscular Atrophies of Childhood"/ (1313)

167 (spinal muscular atroph$ or werdnig hoffman$).ti,ab,kf. (5348)

168 (dubowitz$ or kugelberg-welander$).ti,ab,kf. (480)

169 Bulbar Palsy, Progressive/ (812)

170 (fazio-londe$ or faziolonde$ or progressive bulbar pals$).ti,ab,kf. (128)

171 parkinson disease/ or parkinson disease, secondary/ (70448)

172 (parkinson$ or hypokinetic rigid syndrome$ or hypokinetic rigid disease$ or hypokinetic rigid disorder$ or paralysis agitan$ or shaking pals$).ti,ab,kf. (119027)

173 Pantothenate Kinase-Associated Neurodegeneration/ (496)

174 (pantothenate kinase-associated neurodegenerat$ or PKAN or hallervorden-spatz$).ti,ab,kf. (667)

175 ((neurodegeneration adj3 brain iron accumulation) or NBIA$1).ti,ab,kf. (504)

176 Olivopontocerebellar Atrophies/ (658)

177 (olivopontocerebellar atroph$ or OPCA or olivopontocerebellar degenerat$).ti,ab,kf. (783)

178 (multiple system atrophy adj5 cerebellar).ti,ab,kf. (218)

179 "Diffuse Cerebral Sclerosis of Schilder"/ (2356)

180 (alper$ adj (disease$ or syndrome$ or disorder$)).ti,ab,kf. (174)

181 (progressive sclerosing poliodystroph$ or progressive infantile poliodystroph$).ti,ab,kf. (4)

182 (diffuse cerebral sclerosis adj5 schilder$).ti,ab,kf. (5)

183 Leigh Disease/ (1065)

184 (leigh$ adj (syndrome$ or disease$ or disorder$)).ti,ab,kf. (1434)

185 (subacute necrotizing encephalomyelopath$ or subacute necrotising encephalomyelopath$ or sub-acute necrotizing encephalomyelopath$ or sub-acute necrotising encephalomyelopath$ or SNEM).ti,ab,kf. (192)

186 (aicardi-gouti?res or aicardia-gouti?res).ti,ab,kf. (453)

187 (worster-drought$ or congenital suprabulbar pares$).ti,ab,kf. (38)

188 multiple sclerosis/ or multiple sclerosis, chronic progressive/ or multiple sclerosis, relapsing-remitting/ (58838)

189 (multiple sclerosis or disseminated sclerosis or encephalomyelitis disseminata$).ti,ab,kf. (76528)

190 (demyelinating adj (disease$ or syndrome$ or disorder$)).ti,ab,kf. (8225)

191 exp Epilepsies, Myoclonic/ (4707)

192 myoclonic epileps$.ti,ab,kf. (2643)

193 ((lafora$ or merrf$ or unverricht-lundborg$ or janz$) adj (disease$ or syndrome$ or disorder$)).ti,ab,kf. (805)

194 lennox-gastaut$.ti,ab,kf. (1391)

195 (lennox$ adj (syndrome$ or disease$ or disorder$)).ti,ab,kf. (91)

196 Spasms, Infantile/ (3594)

197 (west$ adj (syndrome$ or disease$ or disorder$)).ti,ab,kf. (1443)

198 Epilepsia Partialis Continua/ (243)

199 (epilepsia partialis continua or kojevnikov$ or epilepsia partialis continuoa or kozhevnikof$).ti,ab,kf. (523)

200 Charcot-Marie-Tooth Disease/ (3873)

201 (charcot-marie-tooth$ or peroneal muscular atroph$).ti,ab,kf. (4616)

202 (progressive neuropathic muscular atroph$ or hereditary peroneal nerve dysfunction$ or peroneal neuropath$).ti,ab,kf. (201)

203 "Hereditary Sensory and Motor Neuropathy"/ (1173)

204 (hereditary sensory adj3 motor neuropath$).ti,ab,kf. (51)

205 (hereditary motor adj3 sensory neuropath$).ti,ab,kf. (862)

206 Refsum Disease, Infantile/ (13)

207 Peroxisomal Disorders/ (567)

208 (infantile refsum or infantile phytanic acid storage).ti,ab,kf. (134)

209 Myasthenic Syndromes, Congenital/ (546)

210 congenital myasth?eni$.ti,ab,kf. (883)

211 Muscular Dystrophy, Duchenne/ (5471)

212 (duchenne muscular dystroph$ or dmd).ti,ab,kf. (11842)

213 exp Muscular Dystrophies, Limb-Girdle/ (1068)

214 (limb-girdle or erb$ muscular dystroph$).ti,ab,kf. (2698)

215 (sarcoglycanopath$ or sarcoglycaopath$).ti,ab,kf. (234)

216 Osteochondrodysplasias/ (5196)

217 (osteochondrodysplas$ or schwartz-jampel or chondrodystrophi$ myotoni$ or myotoni$ chondrodystrophi$).ti,ab,kf. (754)

218 Myotonia Congenita/ (953)

219 (congenita$ myotoni$ or myotoni$ congenita$).ti,ab,kf. (897)

220 (thomsen$ adj (disease$ or disorder$ or syndrome$)).ti,ab,kf. (111)

221 ((recessive adj3 myotoni$) or becker$ myotoni$).ti,ab,kf. (142)

222 Isaacs Syndrome/ (267)

223 (isaac$ adj (syndrome$ or disease$ or disorder$)).ti,ab,kf. (166)

224 neuromyotoni$.ti,ab,kf. (503)

225 Myotonic Disorders/ (275)

226 (paramyotoni$ congenita$ or congenita$ paramyotoni$).ti,ab,kf. (313)

227 (eulenburg$ adj (disease$ or syndrome$ or disorder$)).ti,ab,kf. (1)

228 (myotoni$ adj (disease$ or disorder$ or syndrome$)).ti,ab,kf. (203)

229 pseudomyotoni$.ti,ab,kf. (87)

230 exp Myopathies, Structural, Congenital/ (1300)

231 (congenital adj3 myopath$).ti,ab,kf. (1412)

232 myopathycongenital.ti,ab,kf. (0)

233 ((nemaline or rod) adj3 myopath$).ti,ab,kf. (833)

234 ((central core or mini-core or minicore or multicore or multi-core) adj (disease$ or disorder$ or syndrome$ or myopath$)).ti,ab,kf. (530)

235 fiber type disproportion.ti,ab,kf. (169)

236 fibre type disproportion.ti,ab,kf. (66)

237 Muscular Dystrophies/cn [Congenital] (760)

238 (congenital$ adj5 muscular dystroph$).ti,ab,kf. (1980)

239 ((centronuclear or myotubular) adj myopath$).ti,ab,kf. (726)

240 exp Mitochondrial Myopathies/ (4952)

241 (mitochondrial myopath$ or mitochondrial encephalomyopath$ or chronic progressive external ophthalmopleg$).ti,ab,kf. (3498)

242 ((melas or kearns-sayre$) adj (syndrome$ or disease$ or disorder$)).ti,ab,kf. (1158)

243 Quadriplegia/ and spastic$.ti,ab,kf. (652)

244 (spastic quadriplegi$ or spastic tetraplegi$).ti,ab,kf. (735)

245 Reye Syndrome/ (1625)

246 (reye$ adj (syndrome$ or disease$ or disorder$)).ti,ab,kf. (1713)

247 multiple pterygium.ti,ab,kf. (165)

248 Hypertension, Pulmonary/ and primary$.ti,ab,kf. (3591)

249 ((primary pulmonary or precapillary pulmonary or idiopathic pulmonary) adj (hypertension or ht or arterial hypertension)).ti,ab,kf. (2984)

250 ((primary bronchopulmonary or precapillary bronchopulmonary or idiopathic bronchopulmonary) adj (hypertension or ht or arterial hypertension)).ti,ab,kf. (0)

251 ((primary lung or precapillary lung or idiopathic lung) adj (hypertension or ht or arterial hypertension)).ti,ab,kf. (2)

252 ipah.ti,ab,kf. (1078)

253 Cardiomyopathy, Dilated/ (15666)

254 ((congestive or dilated) adj cardiomyopath$).ti,ab,kf. (18291)

255 exp Cardiomyopathy, Hypertrophic/ (14912)

256 (hypertrophic adj cardiomyopath$).ti,ab,kf. (13802)

257 Cardiomyopathies/cn [Congenital] (167)

258 (congenital adj3 cardiomyopath$).ti,ab,kf. (622)

259 Cardiomyopathy, Restrictive/ (855)

260 (restrictive cardiomyopath$ or obliterative cardiomyopath$ or constrictive cardiomyopath$).ti,ab,kf. (1360)

261 exp Pulmonary Fibrosis/ (23526)

262 (pulmonary fibros$ or lung fibros$ or bronchopulmonary fibros$ or fibrosing alveolit$ or interstitial pneumonit$).ti,ab,kf. (24537)

263 Respiratory Insufficiency/ (32110)

264 (respiratory adj (failure$ or insufficienc$)).ti,ab,kf. (39605)

265 "Cystic Adenomatoid Malformation of Lung, Congenital"/ (943)

266 ((cystic lung or cystic pulmonary or cystic bronchopulmonary) adj (disease$ or disorder or syndrome$)).ti,ab,kf. (403)

267 (bronchogenic cyst$ or bronchopulmonary foregut malformation$).ti,ab,kf. (1758)

268 cystic adenomatoid malformation$.ti,ab,kf. (1009)

269 lobar emphysem$.ti,ab,kf. (637)

270 (pulmonary sequestration$ or bronchopulmonary sequestration$ or lung sequestration$ or extralobar sequestration$ or extra-lobar sequestration$ or intralobar sequestration$ or intra-lobar sequestration$).ti,ab,kf. (2228)

271 pulmolithias$.ti,ab,kf. (5)

272 exp Liver Failure/ (25322)

273 ((liver$1 or hepatic) adj3 fail$).ti,ab,kf. (31092)

274 exp Liver Cirrhosis/ (89334)

275 (cirrhosis adj3 liver$1).ti,ab,kf. (42795)

276 Hepatic Veno-Occlusive Disease/ (1359)

277 ((veno-occlusive or venous occlusive) adj (disease$ or syndrome$ or disorder$)).ti,ab,kf. (2496)

278 Exocrine Pancreatic Insufficiency/ (2065)

279 (swachman-diamond or shwachman-bodian or schwachmann-diamond or shwachmann-bodian).ti,ab,kf. (79)

280 Wegener Granulomatosis/ (6950)

281 wegener$ granulomatos$.ti,ab,kf. (5902)

282 (granulomatos$ adj3 polyangiit$).ti,ab,kf. (2590)

283 Osteolysis, Essential/ (832)

284 essential osteolys$.ti,ab,kf. (27)

285 ((gorham$ or gorham-stout$ or vanishing bone or phantom bone) adj (disease$ or syndrome$ or disorder)).ti,ab,kf. (309)

286 ((arc or arthrogryposis renal dysfunction cholestasis) adj (disease$ or syndrome$ or disorder)).ti,ab,kf. (99)

287 Cerebral Hemorrhage/cn [Congenital] (116)

288 Cerebral Hemorrhage, Traumatic/ (342)

289 Cerebral Hemorrhage/ and Birth Injuries/ (207)

290 (cerebral h?emorrhage$ and (birth$ adj3 injur$)).ti,ab,kf. (43)

291 Asphyxia Neonatorum/ (7691)

292 asphyxia neonatorum.ti,ab,kf. (1393)

293 ((perinatal$ or neonatal$ or birth$) adj3 asphyxia$).ti,ab,kf. (5320)

294 Rubella Syndrome, Congenital/ (756)

295 congenital rubella.ti,ab,kf. (1727)

296 exp Cytomegalovirus Infections/cn [Congenital] (1766)

297 (congenital adj (cytomegalovirus$ or cmv)).ti,ab,kf. (1855)

298 Chickenpox/cn [Congenital] (241)

299 exp Herpes Zoster/cn [Congenital] (34)

300 Herpesvirus 3, Human/ and congenital$.ti,ab,kf. (93)

301 ((congenital or fetal or foetal) adj3 (varicella$ or chicken pox$ or VZV)).ti,ab,kf. (268)

302 Toxoplasmosis, Congenital/ (2663)

303 congenital toxoplasmos$.ti,ab,kf. (1902)

304 exp Hypoxia, Brain/ (12809)

305 ((brain$ or cerebral) adj3 hypoxi$).ti,ab,kf. (7415)

306 Renal Insufficiency/cn [Congenital] (15)

307 Acute Kidney Injury/cn [Congenital] (23)

308 Renal Insufficiency, Chronic/cn [Congenital] (11)

309 Kidney Failure, Chronic/cn [Congenital] (41)

310 (congenital$ adj3 (kidney failure$ or renal failure$ or kidney insufficienc$ or renal insufficienc$)).ti,ab,kf. (78)

311 (congenital$ adj3 (kidney disease$ or renal disease$)).ti,ab,kf. (271)

312 Anencephaly/ (3048)

313 (anencephal$ or meroanencephal$ or craniorachischis$).ti,ab,kf. (3088)

314 (aprosencephal$ adj3 open cranium).ti,ab,kf. (0)

315 Encephalocele/ (3657)

316 (encephalocele$ or cranium bifidum).ti,ab,kf. (2489)

317 Dandy-Walker Syndrome/ (1057)

318 dandy-walker$.ti,ab,kf. (1322)

319 Acrocallosal Syndrome/ (84)

320 (acrocallosal or acro-callosal or acrocolossal or acro colossal).ti,ab,kf. (96)

321 Aicardi Syndrome/ (84)

322 (aicardi$ adj (syndrome$ or disease$ or disorder$)).ti,ab,kf. (329)

323 Holoprosencephaly/ (1178)

324 (holoprosencephal$ or arhinencephal$ or holosprosencephal$).ti,ab,kf. (2063)

325 Hydranencephaly/ (453)

326 (hydranencephal$ or hydrancephal$ or hydroanencephal$).ti,ab,kf. (543)

327 exp Lissencephaly/ (507)

328 Microcephaly/ (5268)

329 (lissencephal$ or walker-warburg$ or miller-dieker$ or norman-robert$ or microlissencephal$).ti,ab,kf. (1886)

330 ((fukuyama$ or muscle-eye-brain) adj (syndrome$ or disease$ or disorder$)).ti,ab,kf. (189)

331 "Malformations of Cortical Development"/ (1318)

332 (microgyria$ or microgyrus or micro-gyria$ or micro-gyrus).ti,ab,kf. (172)

333 (pachygyria$ or pachgyria$).ti,ab,kf. (476)

334 agyria$.ti,ab,kf. (200)

335 Septo-Optic Dysplasia/ (200)

336 ((septo-optic or septooptic) adj dysplas$).ti,ab,kf. (465)

337 de morsier$.ti,ab,kf. (83)

338 (schizencephal$ or schizzencephal$).ti,ab,kf. (472)

339 Arnold-Chiari Malformation/ (3446)

340 chiari$ malformation$.ti,ab,kf. (2567)

341 Truncus Arteriosus, Persistent/ (838)

342 (truncus or common arterial trunk$).ti,ab,kf. (2435)

343 "Transposition of Great Vessels"/ (7038)

344 ((transposition$ or dextrotransposition$ or dtransposition$ or levotransposition$ or ltransposition$) adj3 (great arter$ or main arter$ or aorta$ or pulmonary arter$ or great vessel$ or main vessel$)).ti,ab,kf. (6632)

345 (dextro-tga or d-tga or levo-tga or l-tga).ti,ab,kf. (325)

346 (double inlet adj3 ventricle$).ti,ab,kf. (353)

347 DILV.ti,ab,kf. (39)

348 single ventricle$.ti,ab,kf. (3451)

349 Heart Defects, Congenital/ and Atrial Appendage/ (78)

350 (isomerism adj3 atrial appendage$).ti,ab,kf. (33)

351 (aspleni$ or polyspleni$ or poly-spleni$).ti,ab,kf. (2218)

352 "Tetralogy of Fallot"/ (9248)

353 (tetralogy adj3 fallot$).ti,ab,kf. (9860)

354 Eisenmenger Complex/ (1098)

355 (eisenmenger$ or tardive cyanos$ or eisenmeyer$).ti,ab,kf. (1353)

356 (pentalogy adj3 fallot$).ti,ab,kf. (79)

357 Pulmonary Atresia/ (1458)

358 ((pulmonary or bronchopulmonary or lung$) adj3 atresia$).ti,ab,kf. (3375)

359 Tricuspid Atresia/ (576)

360 ((tricuspid or tri) adj3 atresia$).ti,ab,kf. (1476)

361 Ebstein Anomaly/ (1929)

362 (ebstein$ adj (anomal$ or malformation$)).ti,ab,kf. (2092)

363 Hypoplastic Left Heart Syndrome/ (2423)

364 (hypoplastic left heart adj (syndrome$ or disease$ or disorder$)).ti,ab,kf. (2976)

365 ((aortic or aorta$) adj3 atresia$).ti,ab,kf. (615)

366 (mitral adj3 atresia$).ti,ab,kf. (329)

367 ((absence$ or absent$) adj3 (aorta$ or aortic)).ti,ab,kf. (543)

368 (aplas$ adj3 (aorta$ or aortic)).ti,ab,kf. (8)

369 exp Aortic Aneurysm/cn [Congenital] (245)

370 (((aorta$ or aortic) adj3 aneurys$) and congenital$).ti,ab,kf. (805)

371 (hypoplas$ adj3 (aorta$ or aortic)).ti,ab,kf. (987)

372 (convulsion$ adj3 (aorta$ or aortic)).ti,ab,kf. (1)

373 (persistent right adj3 (aorta$ or aortic)).ti,ab,kf. (81)

374 ((anomalous pulmonary venous or anamolous pulmonary venous) adj (connection or drainage or return)).ti,ab,kf. (2876)

375 ((absence$ or absent$) adj3 vena$ cava$).ti,ab,kf. (332)

376 (persistent left adj3 cardinal vein$).ti,ab,kf. (5)

377 Scimitar Syndrome/ (802)

378 ((scimitar$ or pulmonary venolobar) adj (syndrome$ or disease$ or disorder$)).ti,ab,kf. (638)

379 (arteriovenous malformations/ or intracranial arteriovenous malformations/) and bilateral.ti,ab,kf. (396)

380 ((bilateral AV or bilateral arteriovenous or bilateral arterio-venous) adj3 malform$).ti,ab,kf. (13)

381 ((trachea$ or windpipe$ or wind-pipe$) adj3 atresia$).ti,ab,kf. (142)

382 Tracheal Stenosis/ (5720)

383 ((trachea$ or laryngotrachea$ or glottic or subglottic or sub-glottic) adj3 stenosis).ti,ab,kf. (5354)

384 Bronchopulmonary Dysplasia/ (4768)

385 ((lung$ or pulmonary or bronchopulmonary) adj3 (hypoplas$ or dysplas$)).ti,ab,kf. (11385)

386 ((absence$ or absent$) adj3 (esophag$ or oesophag$ or foodpipe or food-pipe$ or gullet$)).ti,ab,kf. (500)

387 Intestinal Atresia/ (2353)

388 (duoden$ adj3 atresia$).ti,ab,kf. (817)

389 ((absence$ or absent$) adj3 (intestin$ or gastrointestin$)).ti,ab,kf. (1045)

390 ((intestin$ or gastrointestin$) adj3 atresia$).ti,ab,kf. (1223)

391 ((intestin$ or gastrointestin$) adj3 stenos$).ti,ab,kf. (739)

392 (cloaca$ adj3 (abnor$ or malform$ or anomal$)).ti,ab,kf. (440)

393 (cloaca$ adj3 exopthalmo$).ti,ab,kf. (0)

394 Biliary Atresia/ (3116)

395 (biliary adj3 atresia$).ti,ab,kf. (4532)

396 (extrahepatic ductopen$ or extra-hepatic ductopen$ or progressive obliterative cholangiopath$).ti,ab,kf. (5)

397 (biliary adj3 hypoplas$).ti,ab,kf. (73)

398 (alagille$ adj3 atresia$).ti,ab,kf. (36)

399 ((absence$ or absent$) adj3 kidney$).ti,ab,kf. (933)

400 (potter$ adj (sequence$ or syndrome$ or disease$ or disorder$)).ti,ab,kf. (343)

401 Oligohydramnios/ (1254)

402 oligohydramn$.ti,ab,kf. (2653)

403 Multicystic Dysplastic Kidney/ (485)

404 ((kidney$ or renal) adj3 dysplas$).ti,ab,kf. (3056)

405 ((meckel$ or meckelgruber$ or gruber$) adj (syndrome$ or disease$ or disorder$)).ti,ab,kf. (484)

406 dysencephalia splanchnocystica$.ti,ab,kf. (7)

407 (pena-shokeir$ or penn-shokeir$).ti,ab,kf. (103)

408 (larsen$ adj (syndrome$ or disease$ or disorder$)).ti,ab,kf. (258)

409 Acrocephalosyndactylia/ (1570)

410 acrocephalosyndactyl$.ti,ab,kf. (304)

411 (pfeiffer$ adj (syndrome$ or disease$ or syndrome$)).ti,ab,kf. (394)

412 Short Rib-Polydactyly Syndrome/ (144)

413 short rib$1.ti,ab,kf. (333)

414 (saldino-noonan$ or majewski$ or verma-naumoff$ or beemer-langer$).ti,ab,kf. (194)

415 (jeune$ adj (syndrome$ or disease$ or disorder$)).ti,ab,kf. (192)

416 asphyxiating thoracic dysplas$.ti,ab,kf. (54)

417 exp Chondrodysplasia Punctata/ (1008)

418 chondrodysplasia punctata$.ti,ab,kf. (684)

419 ((conradi$ or h?nermann$ or happle$) adj3 (syndrome$ or disease$ or disorder$)).ti,ab,kf. (209)

420 Osteogenesis Imperfecta/ (4654)

421 osteogenesis imperfecta.ti,ab,kf. (4971)

422 ((brittle bone or lobstein$) adj (disease$ or disorder$ or syndrome$)).ti,ab,kf. (250)

423 Osteochondrodysplasias/ (5196)

424 (spondyloepimetaphyseal or spondyloepiphyseal or spendylo metaphyseal).ti,ab,kf. (718)

425 Hernia, Umbilical/ (3743)

426 (omphalocele$ or omphalocoele$ or exomphalos).ti,ab,kf. (2469)

427 (hernia$ adj3 umbilic$).ti,ab,kf. (2444)

428 Gastroschisis/ (1289)

429 gastroschis$.ti,ab,kf. (2383)

430 Ichthyosis, Lamellar/ (794)

431 (lamellar$ adj3 ichthyos$).ti,ab,kf. (452)

432 ((harlequin$ or harloquin$) adj3 (ichthyos$ or baby or babies or f?etus$)).ti,ab,kf. (297)

433 (ichthyosis congenita$ or ichthyosis fetalis or keratosis diffusa fetalis).ti,ab,kf. (86)

434 exp Epidermolysis Bullosa/ (5009)

435 epidermolysis bullosa$.ti,ab,kf. (5396)

436 (johanson-blizzard$ or johanna-blizzard$).ti,ab,kf. (97)

437 Xeroderma Pigmentosum/ (3494)

438 xeroderma pigmentosum.ti,ab,kf. (4352)

439 Ectodermal Dysplasia/ (3403)

440 lacrimo-auriculo-dento-digital.ti,ab,kf. (53)

441 ectodermal dysplas$.ti,ab,kf. (2917)

442 ((ladd or eec) adj (syndrome$ or disease$ or disorder$)).ti,ab,kf. (252)

443 Sturge-Weber Syndrome/ (1259)

444 (sturge-weber or encephalotrigeminal angiomatos$).ti,ab,kf. (1574)

445 Pierre Robin Syndrome/ (1382)

446 pierre robin$.ti,ab,kf. (1242)

447 Acrocephalosyndactylia/ (1570)

448 (acrocephalosyndact$ or acrocephalopolysyndact$).ti,ab,kf. (343)

449 ((apert$ or crouzon$ or saethre-chotzen$ or noack$ or carpenter$ or sakati-nyhan-tisdale$ or goodman$) adj (syndrome$ or disorder$ or disease$)).ti,ab,kf. (2040)

450 Fraser Syndrome/ (42)

451 (fraser$ adj (syndrome$ or disease$ or disorder$)).ti,ab,kf. (212)

452 cryptophthalmos.ti,ab,kf. (201)

453 (cyclopia$1 or cyclocephal$ or synophthalmi$).ti,ab,kf. (654)

454 Goldenhar Syndrome/ (808)

455 (goldenhar$ or oculo-auriculo-vertebral).ti,ab,kf. (871)

456 Mobius Syndrome/ (339)

457 ((m?bius$ or moebius$) adj (syndrome$ or disease$ or disorder$)).ti,ab,kf. (577)

458 Orofaciodigital Syndromes/ (528)

459 (orofaciodigital or oro-facial-digital or oral-facial-digital or papillon-league$ or psaume$).ti,ab,kf. (470)

460 (robin$ adj (syndrome$ or disorder$ or disease$)).ti,ab,kf. (761)

461 (freeman-sheldon$ or distal arthrogrypos$ or craniocarpotarsal dysplas$ or craniocarpotarsal dystroph$ or canio-carpo-tarsal or windmill-vane-hand$ or whistling-face).ti,ab,kf. (442)

462 De Lange Syndrome/ (812)

463 ((de lange$ or bushy$) adj (syndrome$ or disorder$ or disease$)).ti,ab,kf. (966)

464 amsterdam dwarfism.ti,ab,kf. (9)

465 (aarskog or faciodigitogenital or facio-digito-genital or facial digital genital or shawl scrotum or faciogenital or facio-genital).ti,ab,kf. (201)

466 Cockayne Syndrome/ (856)

467 (cockayne$ or neill-dingwall$).ti,ab,kf. (1342)

468 (cerebro-oculo-facio-skeletal or cerebro-oculo-facial-skeletal).ti,ab,kf. (62)

469 (dubowitz$ adj (syndrome$ or disease$ or disorder$)).ti,ab,kf. (104)

470 (robinow$ or robinhow$).ti,ab,kf. (214)

471 (f?etal face or f?etal facies or f?etal faces or acral dysostos$ or mesomelic dwarfism or covesdem$).ti,ab,kf. (259)

472 Silver-Russell Syndrome/ (238)

473 (silver-russell$ or russell-silver$).ti,ab,kf. (723)

474 (silver$ adj (syndrome$ or disease$ or disorder$)).ti,ab,kf. (335)

475 ((seckel$ or harper$) adj (syndrome$ or disease$ or disorder$)).ti,ab,kf. (254)

476 (microcephalic primordial dwarfism or bird-headed dwarf$ or virchow-seckel dwarfism).ti,ab,kf. (84)

477 Smith-Lemli-Opitz Syndrome/ (561)

478 (smith-lemli-opitz$ or dehydrocholesterol reductase deficien$).ti,ab,kf. (812)

479 Prader-Willi Syndrome/ (2890)

480 (prader-willi$ or pradar-willi$).ti,ab,kf. (3576)

481 Rubinstein-Taybi Syndrome/ (507)

482 (rubinstein-taybi$ or rubenstein-tabyii$ or broad thumb-hallux).ti,ab,kf. (700)

483 ((rubinstein$ or rubenstein$) adj2 (syndrome$ or disease$ or disorder$)).ti,ab,kf. (696)

484 Nephritis, Hereditary/ (1927)

485 (alport$ adj (syndrome$ or disease$ or disorder$)).ti,ab,kf. (1857)

486 (hereditary nephritis or h?emorrhagic familial nephritis).ti,ab,kf. (323)

487 (hereditary deafness adj3 nephropath$).ti,ab,kf. (0)

488 (h?ematuria adj3 nephropath$ adj3 deafness).ti,ab,kf. (5)

489 Laurence-Moon Syndrome/ (559)

490 laurence-moon$.ti,ab,kf. (479)

491 Bardet-Biedl Syndrome/ (622)

492 (bardet-biedl$ or biedl-bardet$).ti,ab,kf. (1197)

493 Zellweger Syndrome/ (599)

494 zellweger$.ti,ab,kf. (1005)

495 ((cerebrohepatorenal or cerebro-hepato-renal) adj (syndrome$ or disease$ or disorder$)).ti,ab,kf. (156)

496 (edward$ adj (syndrome$ or disease$ or disorder$)).ti,ab,kf. (311)

497 "trisomy 18".ti,ab,kf. (2410)

498 (patau$ adj (syndrome$ or disease$ or disorder$)).ti,ab,kf. (236)

499 ("trisomy 13" or "trisomy D").ti,ab,kf. (1631)

500 "trisomy 22".ti,ab,kf. (294)

501 "trisomy 9".ti,ab,kf. (295)

502 "trisomy 10".ti,ab,kf. (94)

503 duplication syndrome$.ti,ab,kf. (445)

504 (("chromosome 8" or "chr 8") adj5 duplicat$).ti,ab,kf. (45)

505 Chromosome Duplication/ (934)

506 exp X Chromosome/ab [Abnormalities] (1)

507 exp X Chromosome/ and duplicat$.ti,ab,kf. (857)

508 (("chromosome x" or "chr x") and duplicat$).ti,ab,kf. (90)

509 (chromosom$ abnormality adj5 duplicat$).ti,ab,kf. (23)

510 "tetrasomy 5p".ti,ab,kf. (10)

511 (tetrasomy adj3 mosaic$).ti,ab,kf. (153)

512 Chromosomes, Human, Pair 5/ and Mosaicism/ (40)

513 Tetrasomy/ (67)

514 Trisomy/ and (chromosomes, human, pair 9/ or chromosomes, human, pair 10/ or chromosomes, human, pair 13/ or Chromosomes, Human, Pair 18/ or chromosomes, human, pair 22/) (2941)

515 Chromosome Deletion/ and Chromosomes, Human, Pair 4/ (498)

516 (delet$ adj5 short arm adj5 "chrom$ 4").ti,ab,kf. (92)

517 Wolf-Hirschhorn Syndrome/ (158)

518 ((wolf-hirschhorn$ or wolff hirschorn$ or chromosome deletion dillan$ or pitt-rogers-dank$ or pitt$) adj3 (syndrome$ or disease$ or disorder$)).ti,ab,kf. (849)

519 Cri-du-Chat Syndrome/ (661)

520 ((cri du chat$ or crying cat$ or 5p or lejeune$) adj3 (syndrome$ or disease$ or disorder$)).ti,ab,kf. (594)

521 Jacobsen Distal 11q Deletion Syndrome/ (76)

522 ((jacobsen$ or 11q deletion) adj5 (syndrome$ or disease$ or disorder$)).ti,ab,kf. (153)

523 exp Monosomy/ and Chromosomes, Human, Pair 9/ (769)

524 (9p minus or 9p deletion).ti,ab,kf. (117)

525 (alfi$ adj (syndrome$ or disease$ or disorder$)).ti,ab,kf. (3)

526 (degouchy$ or de gouchy$ or degrouchy$ or de grouchy$).ti,ab,kf. (14)

527 distal 18q.ti,ab,kf. (21)

528 Hypoventilation/cn [Congenital] (245)

529 (ondine$ curse or congenital central hypoventilation or primary alveolar hypoventilation).ti,ab,kf. (721)

530 Graft vs Host Disease/ and (Chronic Disease/ or chronic$.ti,ab,kf.) (7540)

531 (((graft vs host or graft versus host) adj (disease$ or syndrome$ or disorder)) and chronic$).ti,ab,kf. (8581)

532 or/1-531 (1069270)

533 exp HIV/ (98876)

534 exp HIV Infections/ (283039)

535 (HIV or human immunodeficiency virus$).ti,ab,kf. (333568)

536 (htlv or human t-lymphotropic virus$ or human t cell lymphotropic virus$).ti,ab,kf. (13991)

537 (acquired immune deficiency syndrome$ or acquired immunodeficiency syndrome$).ti,ab,kf. (25532)

538 (AIDS adj3 (virus$ or infection$)).ti,ab,kf. (8466)

539 (AIDS adj (related or associated)).ti,ab,kf. (9722)

540 exp Neoplasms/ (3348748)

541 (cancer$ or carcin$ or tumor$ or tumour$ or neoplas$ or adenocarcin$ or oncol$ or malignan$).ti,ab,kf. (3470935)

542 Cystic Fibrosis/ (35263)

543 (cystic fibrosis or fibrocystic or fibro-cystic or mucoviscidosis or cf).ti,ab,kf. (70589)

544 Cerebral Palsy/ (20602)

545 (cerebr$ adj3 pals$).ti,ab,kf. (23549)

546 Muscle Spasticity/ (9153)

547 spasticit$.ti,ab,kf. (11614)

548 Quadriplegia/ (8027)

549 (spastic$ and (quadripleg$ or tetrapleg$)).ti,ab,kf. (1276)

550 exp Renal Insufficiency/ (172905)

551 ((kidney$ or renal) adj3 (failure$ or insufficienc$)).ti,ab,kf. (121429)

552 (end stage adj3 (kidney or renal)).ti,ab,kf. (43790)

553 (("stage 5" or "stage V") adj3 (kidney or renal)).ti,ab,kf. (631)

554 (ESRD or ESKD or ESRF or ESKF or CRF or CKF).ti,ab,kf. (33557)

555 or/533-554 (5129239)

556 Health Services/ or Adolescent Health Services/ or Child Care/ or Community Health Services/ or Health Services for Persons with Disabilities/ (67314)

557 Nursing Care/ or Home nursing/ or Nurses, Community Health/ or "Hospice and Palliative Care Nursing"/ or Nursing Services/ or Home Care Services/ or Nursing Service, Hospital/ or House Calls/ (89147)

558 ("hospital at home" or "home adj2 visit* or home care or home-care or domiciliary care or house-call* or house adj2 call$").ti,ab,kw. (434)

559 ((community or district or home) adj nursing).ti,ab. (3691)

560 exp Primary Health Care/ or Family Practice/ (218122)

561 (family practice or GP practice or general practice or primary care).ti,ab. (149820)

562 Community Health Centers/ or Ambulatory Care Facilities/ or Outpatients/ (41619)

563 (health centre$ or health center$ or health clinic$ or child$ clinic$ or outpatient clinic$ or out-patient clinic$).ti,ab,kw. (74826)

564 exp Hospitals/ or Hospices/ or Nursing Homes/ or Intermediate Care Facilities/ or Skilled Nursing Facilities/ or Long-Term Care/ (332203)

565 (hospital$ or hospice$ or nursing home$ or intermediate care or long-term care or long term care).ti,ab,kw. (1345757)

566 (inpatient$ ward$ or in-patient$ ward$ or inpatient facilit$ or in-patient$ facilit$).ti,ab. (2062)

567 Terminal Care/ or Palliative Care/ or Hospice Care/ or Critical Care/ or Intensive Care Units, Pediatric/ (136041)

568 ((critical or "end of life" or EOL or intensive or palliative or terminal or hospice) adj2 care).ti,ab,kw. (203470)

569 EOLC.ti,ab,kw. (159)

570 556 or 557 or 558 or 559 or 560 or 561 or 562 or 563 or 564 or 565 or 566 or 567 or 568 or 569 (2051110)

571 health personnel/ or allied health personnel/ or community health workers/ or home health aides/ or licensed practical nurses/ or nursing assistants/ or physical therapist assistants/ or medical staff/ (66714)

572 nurses/ or nurse practitioners/ or family nurse practitioners/ or pediatric nurse practitioners/ or nurses, pediatric/ or nurses, neonatal/ or nurses, community health/ or nursing staff/ or nursing staff, hospital/ or exp physicians/ (257582)

573 (doctor$ or physician$ or general practitioner$ or medical practitioner$).ti,ab. (540415)

574 (nurse$ or nursing practitioner$ or nursing staff or hospice staff).ti,ab. (281869)

575 571 or 572 or 573 or 574 (942660)

576 qualitative research/ (55827)

577 nursing evaluation research/ or nursing methodology research/ (25538)

578 qualitativ$.ti,ab,kf. (279545)

579 interviews as topic/ (62465)

580 interview$.ti,ab,kf. (359712)

581 focus groups/ (30034)

582 focus group$.ti,ab,kf. (47066)

583 (grounded theor$ or grounded study or grounded studies or grounded research or grounded analys$).ti,ab,kf. (11850)

584 phenomenol$.ti,ab,kf. (26736)

585 (ethnograph$ or ethnonurs$ or ethno-graph$ or ethno-nurs$).ti,ab,kf. (11149)

586 (story or stories or storytelling or narrative$ or narration$).ti,ab,kf. (73732)

587 (open-ended or open question$ or text$).ti,ab,kf. (177729)

588 Narration/ (8389)

589 (discourse$ analys$ or discurs$ analys$).ti,ab,kf. (2012)

590 content$ analys$.ti,ab,kf. (30035)

591 ethnological.ti,ab,kf. (224)

592 purposive sampl$.ti,ab,kf. (7844)

593 (constant comparative or constant comparison$).ti,ab,kf. (4753)

594 theoretical sampl$.ti,ab,kw. (701)

595 (theme$ or thematic$).ti,ab,kf. (114356)

596 (emic or etic or hermeneutic$ or heuristic$ or semiotic$).ti,ab,kf. (17315)

597 data saturat$.ti,ab,kf. (974)

598 participant observ$.ti,ab,kf. (4579)

599 cluster sampl$.ti,ab,kf. (7542)

600 (humanistic$ or existential$ or experiential$ or paradigm$).ti,ab,kf. (156630)

601 (social construct$ or postmodern$ or post-modern$ or poststructural or post-structural$ or feminis$ or constructivis$).ti,ab,kf. (9529)

602 (action research or cooperative inquir$ or co-operative inquir$).ti,ab,kf. (4333)

603 human science.ti,ab,kf. (253)

604 biographical method$.ti,ab,kf. (27)

605 conversation$.ti,ab,kf. (19611)

606 life world.ti,ab,kf. (342)

607 theoretical saturation.ti,ab,kf. (212)

608 group discussion$.ti,ab,kf. (14721)

609 direct observation$.ti,ab,kf. (12261)

610 mixed method$.ti,ab,kf. (22521)

611 (observational method$ or observational approach$).ti,ab,kf. (1019)

612 (life experience$ or personal experience$ or patient$ experience$ or lived experience$ or actual experience$ or real experience$ or "experience of patient$" or "experiences of patient$").ti,ab,kf. (91799)

613 ((personal or patient$) adj account$).ti,ab,kf. (3432)

614 ("account of patient$" or "accounts of patient$").ti,ab,kf. (2289)

615 ((personal or patient$) adj (perception$ or perspective$)).ti,ab,kf. (21429)

616 ("perspective of patient$" or "perspectives of patient$" or "perception of patient$" or "perceptions of patient$").ti,ab,kf. (5825)

617 ((personal or patient$) adj opinion$).ti,ab,kf. (2064)

618 ("opinion of patient$" or "opinions of patient$").ti,ab,kf. (912)

619 (patient$ adj (need or needs or needed or needing or requir$)).ti,ab,kf. (86929)

620 ("need of patient$" or "needs of patient$" or "requirement of patient$" or "requirements of patient$").ti,ab,kf. (9542)

621 ((patient$ or personal) and (experience or experiences or perspective$ or perception$ or opinion$ or account or accounts or need or needs or needed or needing or requir$)).ti. (72123)

622 ((questionnaire$ or survey$) and patient$ and (experience or experiences or perspective$ or perception$ or opinion$ or account or accounts or need or needs or needed or needing or requir$)).ti,ab,kf. (161271)

623 key informant$.ti,ab,kf. (7643)

624 (field study or field studies or field research$ or field work$ or fieldwork$).ti,ab,kf. (23041)

625 (semi-structured or semistructured or unstructured or un-structured or informal or in-depth or indepth).ti,ab,kf. (151346)

626 "face-to-face".ti,ab,kf. (27945)

627 ((guide or structured) adj5 (discussion$ or questionnaire$)).ti,ab,kf. (24661)

628 (heidegger$ or colaizzi$ or speigelberg$ or van manen$ or van kaam$ or merleau ponty$ or husserl$ or giorgi$ or foucault$ or corbin$ or glaser$).ti,ab,kf. (5132)

629 576 or 577 or 578 or 579 or 580 or 581 or 582 or 583 or 584 or 585 or 586 or 587 or 588 or 589 or 590 or 591 or 592 or 593 or 594 or 595 or 596 or 597 or 598 or 599 or 600 or 601 or 602 or 603 or 604 or 605 or 606 or 607 or 608 or 609 or 610 or 611 or 612 or 613 or 614 or 615 or 616 or 617 or 618 or 619 or 620 or 621 or 622 or 623 or 624 or 625 or 626 or 627 or 628 (1513771)

630 Child/ or Adolescent/ (2810157)

631 Parents/ (63859)

632 (child$ or children$ or adolescen$ or parent$ or parents$ or brother$ or sister$ or sibling$ or teenager$ or young person$ or young people).ti,ab,kw. (1901266)

633 630 or 631 or 632 (3679790)

634 ((pediatric or paediatric) adj3 (critical or EOL or EOLC or "end of life" or palliative or terminal)).ti,ab,kw. (3314)

635 ((child or children) adj3 (critical or EOL or EOLC or "end of life" or palliative or terminal)).ti,ab,kw. (2292)

636 ((adolescen$ or teenager$ or young person$ or young people) adj3 (critical or EOL or EOLC or "end of life" or palliative or terminal)).ti,ab,kw. (1079)

637 ((pediatric or paediatric) adj3 hospice).ti,ab,kw. (142)

638 ((child or children) adj3 hospice).ti,ab,kw. (92)

639 ((adolescen$ or teenager$ or young person$ or young people) adj3 hospice).ti,ab,kw. (11)

640 ((pediatric or paediatric) adj3 ("life limiting" or life-limiting or LLC$)).ti,ab,kw. (44)

641 ((pediatric or paediatric) adj3 ("life threatening" or life-threatening)).ti,ab,kw. (161)

642 ((child$ or children$) adj3 (life-limiting or "life llimiting" or LLC$)).ti,ab,kw. (292)

643 ((child$ or children$) adj3 ("life threatening" or life-threatening)).ti,ab,kw. (1070)

644 ((adolescen$ or teenager$ or young person$ or young people) adj3 (life-limiting or "life llimiting" or LLC$)).ti,ab,kw. (74)

645 ((adolescen$ or teenager$ or young person$ or young people) adj3 ("life threatening" or life-threatening)).ti,ab,kw. (53)

646 634 or 635 or 636 or 637 or 638 or 639 or 640 or 641 or 642 or 643 or 644 or 645 (7733)

647 ((532 or 555) and (570 or 575) and 629 and 633) or (646 and 629) (16141)

648 limit 647 to yr="2010 -Current" (9522)

**PsycINFO via OVID**

Search date 11^th^ August 2020.

Database: APA PsycInfo <1806 to August Week 1 2020>

**Search Strategy**

1 exp Creutzfeldt Jakob Syndrome/ (723)

2 (creutzfeldt-jakob$ or jakob-creutzfeldt$ or cjd or spongiform encephalopath$).ti,ab,id. (1194)

3 (subacute sclerosing panencephalit$ or sub-acute sclerosing panencephalit$ or sspe or subacute sclerosing leukoencephalit$ or sub-acute sclerosing leukoencephalit$ or van bogaert$ leukoencephalit$ or measles inclusion body encephalit$ or mibe).ti,ab,id. (126)

4 (beta adj (thalass?emi$ or thalas?emi$)).ti,ab,id. (102)

5 ((thalass?emi$ or thalas?emi$) adj major).ti,ab,id. (53)

6 ((hypoplastic or aplastic) adj an?emi$).ti,ab,id. (47)

7 (medullary adj3 hypoplas$).ti,ab,id. (2)

8 ((severe or chronic$) adj3 neutropeni$).ti,ab,id. (42)

9 (immun$ deficiency adj (syndrome$ or disease$ or disorder$)).ti,ab,id. (3123)

10 (immunodeficiency adj (syndrome$ or disease$ or disorder$)).ti,ab,id. (830)

11 (digeorge$ or di george$ or sedlackova$ or opitz g-bbb or velocardiofacial or velo-cardiofacial or velo-cardio-facial or shprintzen$ or ctaf).ti,ab,id. (389)

12 ((deletion or vcf or pharyngeal pouch or thymic aplasia or anomaly face) adj (syndrome$ or disease$ or disorder$)).ti,ab,id. (720)

13 ((common variable or late onset) adj3 (immunodeficienc$ or immune deficienc$ or immunoglobulin deficienc$ or hypogammaglobulin$)).ti,ab,id. (11)

14 acquired hypogammaglobulin$.ti,ab,id. (0)

15 cryoglobulin?em$.ti,ab,id. (12)

16 ((autoimmune or failure$) adj3 (polyglandular$ or polyendocrin$)).ti,ab,id. (10)

17 (progeria or hutchinson-gilford$).ti,ab,id. (34)

18 Tyrosinemias.ti,ab,id. (0)

19 tyrosin?em$.ti,ab,id. (22)

20 (maple syrup urine or msud).ti,ab,id. (57)

21 branched chain.ti,ab,id. (158)

22 (bckd adj5 (deficienc$ or ketoacid$ or keto-acid$)).ti,ab,id. (3)

23 hyperleucine-isoleucin$.ti,ab,id. (0)

24 (methylmalonic acid?emi$ or methylmalonic aciduri$ or methyl malonic acid?emi$ or methyl malonic aciduri$).ti,ab,id. (48)

25 (propionic acid?em$ or propionic acidur$ or propionyl-CoA carboxylase deficienc$ or ketotic glycin?em$).ti,ab,id. (14)

26 (adrenoleukodystroph$ or x-ald or schilder-addison$ or addison-schilder$ or adrenomyeloneuropath$).ti,ab,id. (211)

27 ((carnitine palmityltransferase or carnitine palmitoyltransferase or carnitine o-palmityltransferase or carnitine o-palmitoyltransferase) adj3 deficienc$).ti,ab,id. (12)

28 (fanconi$ adj (syndrome$ or disease$ or disorder$)).ti,ab,id. (9)

29 (ocular adj3 (renal or kidney)).ti,ab,id. (4)

30 (cystinos$ or cystine storage or cystine diathes$ or cystine disease$).ti,ab,id. (30)

31 ((lowe or lowes or oculocerebrorenal or cerebrooculorenal or cerebro-oculo-renal) adj3 (syndrome$ or disease$ or disorder$)).ti,ab,id. (16)

32 (molybdenum cofactor deficien$ or molybdenum co-factor deficien$).ti,ab,id. (7)

33 ((sulphite$ or sulfite$) adj3 oxidase deficien$).ti,ab,id. (14)

34 (argininosuccinic acidur$ or argininosuccinic acid?emi$).ti,ab,id. (7)

35 (citrullin?emi$ or citrullinuri$).ti,ab,id. (8)

36 (glutaric acid?emi$ or glutaric aciduri$).ti,ab,id. (48)

37 (glycine encephalopath$ or non-ketotic hyperglycin?emi$ or nonketotic hyperglycin?emi$).ti,ab,id. (36)

38 (arginin?emi$ or arginase deficien$ or hyperarginin?emi$).ti,ab,id. (12)

39 (aminoaciduri$ or aminoacid?emi$).ti,ab,id. (16)

40 (glycogen storage adj (disease$ or syndrome$ or disorder$)).ti,ab,id. (71)

41 (pompe$ adj (disease$ or syndrome$ or disorder$)).ti,ab,id. (84)

42 galactos?emi$.ti,ab,id. (45)

43 (pyruvate dehydrogenase adj3 deficien$).ti,ab,id. (22)

44 (oxalosis and (renal or kidney$)).ti,ab,id. (0)

45 gangliosidos$.ti,ab,id. (68)

46 (sandhoff$ adj (disease$ or syndrome$ or disorder$)).ti,ab,id. (28)

47 tay sach$.ti,ab,id. (91)

48 mucolipidos$.ti,ab,id. (20)

49 (canavan$ leucodystroph$ or aspartoacylase deficien$ or aminoacylase 2 deficien$).ti,ab,id. (0)

50 ((canavan$ or canavan-van bogaert-bertrand$) adj (disease$ or syndrome$ or disorder$)).ti,ab,id. (34)

51 (gaucher$ adj (disease$ or syndrome$ or disorder$)).ti,ab,id. (151)

52 (glucocerebrosidase deficien$ or glucosylceramidase deficien$).ti,ab,id. (3)

53 (metachromatic leukodystroph$ or arylsulfatase A deficien$ or metachromic leukodystroph$).ti,ab,id. (121)

54 (niemann-pick$ or sphingomyelinase deficien$).ti,ab,id. (220)

55 sphingolipidos$.ti,ab,id. (14)

56 (fabry$ adj (disease$ or syndrome$ or disorder$)).ti,ab,id. (129)

57 (angiokeratoma corporis diffusum or alpha-galactosidase A deficien$).ti,ab,id. (5)

58 (krabbe$ adj (disease$ or syndrome$ or disorder$)).ti,ab,id. (77)

59 (globoid cell leukodystroph$ or galactosylceramide lipidos$ or galactosylcerebrosidase deficien$ or galactosylceramidase deficien$).ti,ab,id. (28)

60 (farber$ adj (disease$ or syndrome$ or disorder$)).ti,ab,id. (3)

61 (farber$ lipogranulomatos$ or ceramidase deficien$ or fibrocytic dysmucopolysaccharidos$).ti,ab,id. (2)

62 pelizaeus-merzbacher$.ti,ab,id. (84)

63 (sulfatase deficien$ or sulphatase deficien$ or mucosulfatidos$).ti,ab,id. (9)

64 (austin$ adj (disease$ or syndrome$ or disorder$)).ti,ab,id. (0)

65 sulfatidos$.ti,ab,id. (0)

66 sea-blue histiocyt$.ti,ab,id. (4)

67 (batten$ adj (disease$ or syndrome$ or disorder$)).ti,ab,id. (69)

68 (neuronal ceroid lipofuscinos$ or santavuori-haltia$ or jansky-bielschowsky$ or bielschowsky-jansky$).ti,ab,id. (232)

69 (kuf$ adj (disease$ or syndrome$ or disorder$)).ti,ab,id. (21)

70 spielmeyer vogt$.ti,ab,id. (6)

71 ((cerebrotendineous or cerebrotendinous or cerebrotendious or cerebral) adj3 (xanthomatos$ or cholesteros$)).ti,ab,id. (47)

72 bogaert-scherer-epstein$.ti,ab,id. (0)

73 (wolman$ adj (disease$ or syndrome$ or disorder$)).ti,ab,id. (2)

74 lysosomal acid lipase deficien$.ti,ab,id. (1)

75 mucopolysaccharidos$.ti,ab,id. (151)

76 (hurler$ adj2 (syndrome$ or disease$ or disorder$)).ti,ab,id. (32)

77 (hunter$ adj2 (syndrome$ or disease$ or disorder$)).ti,ab,id. (31)

78 (MPS1 or MPS2 or MPS3 or MPS4 or MPS5 or MPS6 or MPS7 or MPS-1 or MPS-2 or MPS-3 or MPS-4 or MPS-5 or MPS-6 or MPS-7 or MPSI or MPSII or MPSIII or MPSIV or MPSV or MPSVI or MPSVII or MPS-I or MPS-II or MPS-III or MPS-IV or MPS-V or MPS-VI or MPS-VII).ti,ab,id. (96)

79 (beta glucuronidase deficien$ or sly syndrome$ or sly disorder$ or sly disease$).ti,ab,id. (4)

80 (maroteaux-lamy$ or marotaeux-lamy$ or polydystrophic dwarfism).ti,ab,id. (6)

81 (morquio$ or moriquio$ or beta galactosidase deficien$).ti,ab,id. (18)

82 (sanfilippo$ or sanfillipo$).ti,ab,id. (57)

83 (mucolipidos$ or pseudo-hurler$ or pseudohurler$).ti,ab,id. (20)

84 ((inclusion-cell or i-cell) adj (disease$ or syndrome$ or disorder$)).ti,ab,id. (1)

85 (fucosidos$ or fucidos$).ti,ab,id. (9)

86 ((cdg or ctg) adj (disease$ or disorder$ or syndrome$)).ti,ab,id. (6)

87 (carbohydrate-deficient glycoprotein adj (disease$ or disorder$ or syndrome$)).ti,ab,id. (2)

88 (congenital disorder$ adj3 glycosylation).ti,ab,id. (34)

89 ((nyhan$ or kelley-seegmiller$) adj (syndrome$ or disorder$ or disease$)).ti,ab,id. (173)

90 juvenile gout.ti,ab,id. (0)

91 menkes$.ti,ab,id. (73)

92 ((copper transport or steely hair or kinky hair) adj (disease$ or syndrome$ or disorder$)).ti,ab,id. (8)

93 (antitrypsin deficien$ or A1AD).ti,ab,id. (49)

94 (AAT deficien$ or alpha-1 protease deficien$).ti,ab,id. (4)

95 bisalbumin?emi$.ti,ab,id. (0)

96 (congenital generali?ed lipodystroph$ or berardinelli$ or bernardnelli$).ti,ab,id. (15)

97 (landau-kleffner$ or infantile acquired aphasia$ or acquired epileptic aphasia$).ti,ab,id. (220)

98 (aphasia$ adj5 convulsive).ti,ab,id. (25)

99 exp Rett Syndrome/ (886)

100 (rett$ adj (syndrome$ or disease$ or disorder$)).ti,ab,id. (1272)

101 cerebroatrophic hyperammon?emi$.ti,ab,id. (0)

102 exp Huntingtons Disease/ (3241)

103 huntington$.ti,ab,id. (4869)

104 (spinocerebellar ataxia$ or ataxia$ telangiectasia$ or louis-bar$ syndrome$ or louis-bar$ disease$ or louis-bar$ disorder$ or machado-joseph$ or joseph$ disease$ or joseph$ disorder$ or joseph$ syndrome$).ti,ab,id. (1302)

105 exp Ataxia/ (3299)

106 ((friedreich$ or friedrich$) adj3 ataxia$).ti,ab,id. (416)

107 spinocerebellar degenerat$.ti,ab,id. (87)

108 (spinal muscular atroph$ or werdnig hoffman$).ti,ab,id. (543)

109 (dubowitz$ or kugelberg-welander$).ti,ab,id. (44)

110 (fazio-londe$ or faziolonde$ or progressive bulbar pals$).ti,ab,id. (13)

111 exp Parkinson's Disease/ (24935)

112 (parkinson$ or hypokinetic rigid syndrome$ or hypokinetic rigid disease$ or hypokinetic rigid disorder$ or paralysis agitan$ or shaking pals$).ti,ab,id. (34310)

113 (pantothenate kinase-associated neurodegenerat$ or PKAN or hallervorden-spatz$).ti,ab,id. (109)

114 ((neurodegeneration adj3 brain iron accumulation) or NBIA$1).ti,ab,id. (138)

115 (olivopontocerebellar atroph$ or OPCA or olivopontocerebellar degenerat$).ti,ab,id. (85)

116 (multiple system atrophy adj5 cerebellar).ti,ab,id. (68)

117 (alper$ adj (disease$ or syndrome$ or disorder$)).ti,ab,id. (18)

118 (progressive sclerosing poliodystroph$ or progressive infantile poliodystroph$).ti,ab,id. (0)

119 (diffuse cerebral sclerosis adj5 schilder$).ti,ab,id. (0)

120 (leigh$ adj (syndrome$ or disease$ or disorder$)).ti,ab,id. (139)

121 (subacute necrotizing encephalomyelopath$ or subacute necrotising encephalomyelopath$ or sub-acute necrotizing encephalomyelopath$ or sub-acute necrotising encephalomyelopath$ or SNEM).ti,ab,id. (5)

122 (aicardi-gouti?res or aicardia-gouti?res).ti,ab,id. (41)

123 (worster-drought$ or congenital suprabulbar pares$).ti,ab,id. (18)

124 exp Multiple Sclerosis/ (12827)

125 (multiple sclerosis or disseminated sclerosis or encephalomyelitis disseminata$).ti,ab,id. (15908)

126 (demyelinating adj (disease$ or syndrome$ or disorder$)).ti,ab,id. (1385)

127 exp Epilepsy/ (27763)

128 myoclonic epileps$.ti,ab,id. (625)

129 ((lafora$ or merrf$ or unverricht-lundborg$ or janz$) adj (disease$ or syndrome$ or disorder$)).ti,ab,id. (112)

130 lennox-gastaut$.ti,ab,id. (309)

131 (lennox$ adj (syndrome$ or disease$ or disorder$)).ti,ab,id. (14)

132 exp Spasms/ (1071)

133 (west$ adj (syndrome$ or disease$ or disorder$)).ti,ab,id. (279)

134 (epilepsia partialis continua or kojevnikov$ or epilepsia partialis continuoa or kozhevnikof$).ti,ab,id. (87)

135 exp Charcot-Marie-Tooth Disease/ (351)

136 (charcot-marie-tooth$ or peroneal muscular atroph$).ti,ab,id. (607)

137 (progressive neuropathic muscular atroph$ or hereditary peroneal nerve dysfunction$ or peroneal neuropath$).ti,ab,id. (11)

138 (hereditary sensory adj3 motor neuropath$).ti,ab,id. (7)

139 (hereditary motor adj3 sensory neuropath$).ti,ab,id. (86)

140 (infantile refsum or infantile phytanic acid storage).ti,ab,id. (4)

141 exp Myasthenia/ (156)

142 congenital myasth?eni$.ti,ab,id. (100)

143 exp Muscular Dystrophy/ (1382)

144 (duchenne muscular dystroph$ or dmd).ti,ab,id. (724)

145 (limb-girdle or erb$ muscular dystroph$).ti,ab,id. (227)

146 (sarcoglycanopath$ or sarcoglycaopath$).ti,ab,id. (15)

147 (osteochondrodysplas$ or schwartz-jampel or chondrodystrophi$ myotoni$ or myotoni$ chondrodystrophi$).ti,ab,id. (4)

148 exp Myotonia/ (284)

149 (congenita$ myotoni$ or myotoni$ congenita$).ti,ab,id. (47)

150 (thomsen$ adj (disease$ or disorder$ or syndrome$)).ti,ab,id. (8)

151 ((recessive adj3 myotoni$) or becker$ myotoni$).ti,ab,id. (11)

152 (isaac$ adj (syndrome$ or disease$ or disorder$)).ti,ab,id. (22)

153 neuromyotoni$.ti,ab,id. (89)

154 (paramyotoni$ congenita$ or congenita$ paramyotoni$).ti,ab,id. (24)

155 (eulenburg$ adj (disease$ or syndrome$ or disorder$)).ti,ab,id. (0)

156 (myotoni$ adj (disease$ or disorder$ or syndrome$)).ti,ab,id. (22)

157 pseudomyotoni$.ti,ab,id. (7)

158 exp Myopathy/ (929)

159 (congenital adj3 myopath$).ti,ab,id. (129)

160 myopathycongenital.ti,ab,id. (0)

161 ((nemaline or rod) adj3 myopath$).ti,ab,id. (61)

162 ((central core or mini-core or minicore or multicore or multi-core) adj (disease$ or disorder$ or syndrome$ or myopath$)).ti,ab,id. (16)

163 fiber type disproportion.ti,ab,id. (13)

164 fibre type disproportion.ti,ab,id. (5)

165 (congenital$ adj5 muscular dystroph$).ti,ab,id. (178)

166 ((centronuclear or myotubular) adj myopath$).ti,ab,id. (42)

167 (mitochondrial myopath$ or mitochondrial encephalomyopath$ or chronic progressive external ophthalmopleg$).ti,ab,id. (278)

168 ((melas or kearns-sayre$) adj (syndrome$ or disease$ or disorder$)).ti,ab,id. (64)

169 exp Quadriplegia/ (204)

170 (spastic quadriplegi$ or spastic tetraplegi$).ti,ab,id. (153)

171 (reye$ adj (syndrome$ or disease$ or disorder$)).ti,ab,id. (38)

172 multiple pterygium.ti,ab,id. (0)

173 Hypertension/ and primary$.ti,ab,id. (685)

174 ((primary pulmonary or precapillary pulmonary or idiopathic pulmonary) adj (hypertension or ht or arterial hypertension)).ti,ab,id. (6)

175 ((primary bronchopulmonary or precapillary bronchopulmonary or idiopathic bronchopulmonary) adj (hypertension or ht or arterial hypertension)).ti,ab,id. (0)

176 ((primary lung or precapillary lung or idiopathic lung) adj (hypertension or ht or arterial hypertension)).ti,ab,id. (0)

177 ipah.ti,ab,id. (3)

178 ((congestive or dilated) adj cardiomyopath$).ti,ab,id. (117)

179 (hypertrophic adj cardiomyopath$).ti,ab,id. (83)

180 (congenital adj3 cardiomyopath$).ti,ab,id. (3)

181 (restrictive cardiomyopath$ or obliterative cardiomyopath$ or constrictive cardiomyopath$).ti,ab,id. (6)

182 (pulmonary fibros$ or lung fibros$ or bronchopulmonary fibros$ or fibrosing alveolit$ or interstitial pneumonit$).ti,ab,id. (82)

183 (respiratory adj (failure$ or insufficienc$)).ti,ab,id. (775)

184 ((cystic lung or cystic pulmonary or cystic bronchopulmonary) adj (disease$ or disorder or syndrome$)).ti,ab,id. (1)

185 (bronchogenic cyst$ or bronchopulmonary foregut malformation$).ti,ab,id. (2)

186 cystic adenomatoid malformation$.ti,ab,id. (2)

187 lobar emphysem$.ti,ab,id. (1)

188 (pulmonary sequestration$ or bronchopulmonary sequestration$ or lung sequestration$ or extralobar sequestration$ or extra-lobar sequestration$ or intralobar sequestration$ or intra-lobar sequestration$).ti,ab,id. (0)

189 pulmolithias$.ti,ab,id. (0)

190 ((liver$1 or hepatic) adj3 fail$).ti,ab,id. (413)

191 exp "Cirrhosis (Liver)"/ (401)

192 (cirrhosis adj3 liver$1).ti,ab,id. (559)

193 ((veno-occlusive or venous occlusive) adj (disease$ or syndrome$ or disorder$)).ti,ab,id. (9)

194 (swachman-diamond or shwachman-bodian or schwachmann-diamond or shwachmann-bodian).ti,ab,id. (1)

195 wegener$ granulomatos$.ti,ab,id. (25)

196 (granulomatos$ adj3 polyangiit$).ti,ab,id. (9)

197 essential osteolys$.ti,ab,id. (0)

198 ((gorham$ or gorham-stout$ or vanishing bone or phantom bone) adj (disease$ or syndrome$ or disorder)).ti,ab,id. (2)

199 ((arc or arthrogryposis renal dysfunction cholestasis) adj (disease$ or syndrome$ or disorder)).ti,ab,id. (0)

200 exp Cerebral Hemorrhage/ (1971)

201 (cerebral h?emorrhage$ and (birth$ adj3 injur$)).ti,ab,id. (2)

202 asphyxia neonatorum.ti,ab,id. (10)

203 ((perinatal$ or neonatal$ or birth$) adj3 asphyxia$).ti,ab,id. (387)

204 congenital rubella.ti,ab,id. (55)

205 (congenital adj (cytomegalovirus$ or cmv)).ti,ab,id. (68)

206 ((congenital or fetal or foetal) adj3 (varicella$ or chicken pox$ or VZV)).ti,ab,id. (1)

207 congenital toxoplasmos$.ti,ab,id. (8)

208 ((brain$ or cerebral) adj3 hypoxi$).ti,ab,id. (999)

209 (congenital$ adj3 (kidney failure$ or renal failure$ or kidney insufficienc$ or renal insufficienc$)).ti,ab,id. (1)

210 (congenital$ adj3 (kidney disease$ or renal disease$)).ti,ab,id. (2)

211 exp Anencephaly/ (25)

212 (anencephal$ or meroanencephal$ or craniorachischis$).ti,ab,id. (104)

213 (aprosencephal$ adj3 open cranium).ti,ab,id. (0)

214 (encephalocele$ or cranium bifidum).ti,ab,id. (53)

215 dandy-walker$.ti,ab,id. (73)

216 (acrocallosal or acro-callosal or acrocolossal or acro colossal).ti,ab,id. (3)

217 (aicardi$ adj (syndrome$ or disease$ or disorder$)).ti,ab,id. (28)

218 (holoprosencephal$ or arhinencephal$ or holosprosencephal$).ti,ab,id. (54)

219 (hydranencephal$ or hydrancephal$ or hydroanencephal$).ti,ab,id. (32)

220 exp Microcephaly/ (255)

221 (lissencephal$ or walker-warburg$ or miller-dieker$ or norman-robert$ or microlissencephal$).ti,ab,id. (260)

222 ((fukuyama$ or muscle-eye-brain) adj (syndrome$ or disease$ or disorder$)).ti,ab,id. (21)

223 (microgyria$ or microgyrus or micro-gyria$ or micro-gyrus).ti,ab,id. (56)

224 (pachygyria$ or pachgyria$).ti,ab,id. (53)

225 agyria$.ti,ab,id. (18)

226 ((septo-optic or septooptic) adj dysplas$).ti,ab,id. (35)

227 de morsier$.ti,ab,id. (10)

228 (schizencephal$ or schizzencephal$).ti,ab,id. (74)

229 chiari$ malformation$.ti,ab,id. (122)

230 (truncus or common arterial trunk$).ti,ab,id. (26)

231 ((transposition$ or dextrotransposition$ or dtransposition$ or levotransposition$ or ltransposition$) adj3 (great arter$ or main arter$ or aorta$ or pulmonary arter$ or great vessel$ or main vessel$)).ti,ab,id. (41)

232 (dextro-tga or d-tga or levo-tga or l-tga).ti,ab,id. (9)

233 (double inlet adj3 ventricle$).ti,ab,id. (0)

234 DILV.ti,ab,id. (1)

235 single ventricle$.ti,ab,id. (27)

236 (isomerism adj3 atrial appendage$).ti,ab,id. (0)

237 (aspleni$ or polyspleni$ or poly-spleni$).ti,ab,id. (7)

238 (tetralogy adj3 fallot$).ti,ab,id. (45)

239 (eisenmenger$ or tardive cyanos$ or eisenmeyer$).ti,ab,id. (4)

240 (pentalogy adj3 fallot$).ti,ab,id. (0)

241 ((pulmonary or bronchopulmonary or lung$) adj3 atresia$).ti,ab,id. (6)

242 ((tricuspid or tri) adj3 atresia$).ti,ab,id. (6)

243 (ebstein$ adj (anomal$ or malformation$)).ti,ab,id. (9)

244 (hypoplastic left heart adj (syndrome$ or disease$ or disorder$)).ti,ab,id. (30)

245 ((aortic or aorta$) adj3 atresia$).ti,ab,id. (2)

246 (mitral adj3 atresia$).ti,ab,id. (0)

247 ((absence$ or absent$) adj3 (aorta$ or aortic)).ti,ab,id. (1)

248 (aplas$ adj3 (aorta$ or aortic)).ti,ab,id. (0)

249 exp Aorta/ and exp Aneurysms/ (25)

250 (((aorta$ or aortic) adj3 aneurys$) and congenital$).ti,ab,id. (4)

251 (hypoplas$ adj3 (aorta$ or aortic)).ti,ab,id. (0)

252 (convulsion$ adj3 (aorta$ or aortic)).ti,ab,id. (0)

253 (persistent right adj3 (aorta$ or aortic)).ti,ab,id. (0)

254 ((anomalous pulmonary venous or anamolous pulmonary venous) adj (connection or drainage or return)).ti,ab,id. (1)

255 ((absence$ or absent$) adj3 vena$ cava$).ti,ab,id. (0)

256 (persistent left adj3 cardinal vein$).ti,ab,id. (0)

257 ((scimitar$ or pulmonary venolobar) adj (syndrome$ or disease$ or disorder$)).ti,ab,id. (0)

258 (arteriovenous malformations/ or intracranial arteriovenous malformations/) and bilateral.ti,ab,id. (0)

259 ((bilateral AV or bilateral arteriovenous or bilateral arterio-venous) adj3 malform$).ti,ab,id. (0)

260 ((trachea$ or windpipe$ or wind-pipe$) adj3 atresia$).ti,ab,id. (1)

261 ((trachea$ or laryngotrachea$ or glottic or subglottic or sub-glottic) adj3 stenosis).ti,ab,id. (10)

262 ((lung$ or pulmonary or bronchopulmonary) adj3 (hypoplas$ or dysplas$)).ti,ab,id. (191)

263 ((absence$ or absent$) adj3 (esophag$ or oesophag$ or foodpipe or food-pipe$ or gullet$)).ti,ab,id. (2)

264 (duoden$ adj3 atresia$).ti,ab,id. (4)

265 ((absence$ or absent$) adj3 (intestin$ or gastrointestin$)).ti,ab,id. (21)

266 ((intestin$ or gastrointestin$) adj3 atresia$).ti,ab,id. (12)

267 ((intestin$ or gastrointestin$) adj3 stenos$).ti,ab,id. (1)

268 (cloaca$ adj3 (abnor$ or malform$ or anomal$)).ti,ab,id. (1)

269 (cloaca$ adj3 exopthalmo$).ti,ab,id. (0)

270 (biliary adj3 atresia$).ti,ab,id. (19)

271 (extrahepatic ductopen$ or extra-hepatic ductopen$ or progressive obliterative cholangiopath$).ti,ab,id. (0)

272 (biliary adj3 hypoplas$).ti,ab,id. (0)

273 (alagille$ adj3 atresia$).ti,ab,id. (0)

274 ((absence$ or absent$) adj3 kidney$).ti,ab,id. (4)

275 (potter$ adj (sequence$ or syndrome$ or disease$ or disorder$)).ti,ab,id. (2)

276 oligohydramn$.ti,ab,id. (23)

277 ((kidney$ or renal) adj3 dysplas$).ti,ab,id. (8)

278 ((meckel$ or meckelgruber$ or gruber$) adj (syndrome$ or disease$ or disorder$)).ti,ab,id. (4)

279 dysencephalia splanchnocystica$.ti,ab,id. (0)

280 (pena-shokeir$ or penn-shokeir$).ti,ab,id. (1)

281 (larsen$ adj (syndrome$ or disease$ or disorder$)).ti,ab,id. (1)

282 acrocephalosyndactyl$.ti,ab,id. (9)

283 (pfeiffer$ adj (syndrome$ or disease$ or syndrome$)).ti,ab,id. (2)

284 short rib$1.ti,ab,id. (0)

285 (saldino-noonan$ or majewski$ or verma-naumoff$ or beemer-langer$).ti,ab,id. (2)

286 (jeune$ adj (syndrome$ or disease$ or disorder$)).ti,ab,id. (1)

287 asphyxiating thoracic dysplas$.ti,ab,id. (0)

288 chondrodysplasia punctata$.ti,ab,id. (6)

289 ((conradi$ or h?nermann$ or happle$) adj3 (syndrome$ or disease$ or disorder$)).ti,ab,id. (2)

290 osteogenesis imperfecta.ti,ab,id. (51)

291 ((brittle bone or lobstein$) adj (disease$ or disorder$ or syndrome$)).ti,ab,id. (9)

292 (spondyloepimetaphyseal or spondyloepiphyseal or spendylo metaphyseal).ti,ab,id. (6)

293 (omphalocele$ or omphalocoele$ or exomphalos).ti,ab,id. (18)

294 (hernia$ adj3 umbilic$).ti,ab,id. (14)

295 gastroschis$.ti,ab,id. (23)

296 (lamellar$ adj3 ichthyos$).ti,ab,id. (1)

297 ((harlequin$ or harloquin$) adj3 (ichthyos$ or baby or babies or f?etus$)).ti,ab,id. (1)

298 (ichthyosis congenita$ or ichthyosis fetalis or keratosis diffusa fetalis).ti,ab,id. (1)

299 epidermolysis bullosa$.ti,ab,id. (19)

300 (johanson-blizzard$ or johanna-blizzard$).ti,ab,id. (3)

301 xeroderma pigmentosum.ti,ab,id. (40)

302 lacrimo-auriculo-dento-digital.ti,ab,id. (0)

303 ectodermal dysplas$.ti,ab,id. (21)

304 ((ladd or eec) adj (syndrome$ or disease$ or disorder$)).ti,ab,id. (3)

305 (sturge-weber or encephalotrigeminal angiomatos$).ti,ab,id. (126)

306 pierre robin$.ti,ab,id. (16)

307 (acrocephalosyndact$ or acrocephalopolysyndact$).ti,ab,id. (10)

308 ((apert$ or crouzon$ or saethre-chotzen$ or noack$ or carpenter$ or sakati-nyhan-tisdale$ or goodman$) adj (syndrome$ or disorder$ or disease$)).ti,ab,id. (35)

309 (fraser$ adj (syndrome$ or disease$ or disorder$)).ti,ab,id. (1)

310 cryptophthalmos.ti,ab,id. (0)

311 (cyclopia$1 or cyclocephal$ or synophthalmi$).ti,ab,id. (12)

312 (goldenhar$ or oculo-auriculo-vertebral).ti,ab,id. (21)

313 ((m?bius$ or moebius$) adj (syndrome$ or disease$ or disorder$)).ti,ab,id. (60)

314 (orofaciodigital or oro-facial-digital or oral-facial-digital or papillon-league$ or psaume$).ti,ab,id. (3)

315 (robin$ adj (syndrome$ or disorder$ or disease$)).ti,ab,id. (11)

316 (freeman-sheldon$ or distal arthrogrypos$ or craniocarpotarsal dysplas$ or craniocarpotarsal dystroph$ or canio-carpo-tarsal or windmill-vane-hand$ or whistling-face).ti,ab,id. (9)

317 exp Cornelia De Lange Syndrome/ (44)

318 ((de lange$ or bushy$) adj (syndrome$ or disorder$ or disease$)).ti,ab,id. (89)

319 amsterdam dwarfism.ti,ab,id. (1)

320 (aarskog or faciodigitogenital or facio-digito-genital or facial digital genital or shawl scrotum or faciogenital or facio-genital).ti,ab,id. (3)

321 (cockayne$ or neill-dingwall$).ti,ab,id. (32)

322 (cerebro-oculo-facio-skeletal or cerebro-oculo-facial-skeletal).ti,ab,id. (0)

323 (dubowitz$ adj (syndrome$ or disease$ or disorder$)).ti,ab,id. (2)

324 (robinow$ or robinhow$).ti,ab,id. (12)

325 (f?etal face or f?etal facies or f?etal faces or acral dysostos$ or mesomelic dwarfism or covesdem$).ti,ab,id. (4)

326 (silver-russell$ or russell-silver$).ti,ab,id. (17)

327 (silver$ adj (syndrome$ or disease$ or disorder$)).ti,ab,id. (14)

328 ((seckel$ or harper$) adj (syndrome$ or disease$ or disorder$)).ti,ab,id. (9)

329 (microcephalic primordial dwarfism or bird-headed dwarf$ or virchow-seckel dwarfism).ti,ab,id. (3)

330 (smith-lemli-opitz$ or dehydrocholesterol reductase deficien$).ti,ab,id. (41)

331 exp Prader Willi Syndrome/ (545)

332 (prader-willi$ or pradar-willi$).ti,ab,id. (800)

333 (rubinstein-taybi$ or rubenstein-tabyii$ or broad thumb-hallux).ti,ab,id. (58)

334 ((rubinstein$ or rubenstein$) adj2 (syndrome$ or disease$ or disorder$)).ti,ab,id. (59)

335 (alport$ adj (syndrome$ or disease$ or disorder$)).ti,ab,id. (7)

336 (hereditary nephritis or h?emorrhagic familial nephritis).ti,ab,id. (2)

337 (hereditary deafness adj3 nephropath$).ti,ab,id. (0)

338 (h?ematuria adj3 nephropath$ adj3 deafness).ti,ab,id. (0)

339 laurence-moon$.ti,ab,id. (21)

340 (bardet-biedl$ or biedl-bardet$).ti,ab,id. (35)

341 zellweger$.ti,ab,id. (29)

342 ((cerebrohepatorenal or cerebro-hepato-renal) adj (syndrome$ or disease$ or disorder$)).ti,ab,id. (2)

343 (edward$ adj (syndrome$ or disease$ or disorder$)).ti,ab,id. (6)

344 "trisomy 18".ti,ab,id. (42)

345 (patau$ adj (syndrome$ or disease$ or disorder$)).ti,ab,id. (7)

346 ("trisomy 13" or "trisomy D").ti,ab,id. (33)

347 "trisomy 22".ti,ab,id. (9)

348 "trisomy 9".ti,ab,id. (2)

349 "trisomy 10".ti,ab,id. (0)

350 duplication syndrome$.ti,ab,id. (52)

351 (("chromosome 8" or "chr 8") adj5 duplicat$).ti,ab,id. (0)

352 (("chromosome x" or "chr x") and duplicat$).ti,ab,id. (6)

353 (chromosom$ abnormality adj5 duplicat$).ti,ab,id. (2)

354 "tetrasomy 5p".ti,ab,id. (0)

355 (tetrasomy adj3 mosaic$).ti,ab,id. (3)

356 exp Chromosome Disorders/ (10109)

357 (delet$ adj5 short arm adj5 "chrom$ 4").ti,ab,id. (2)

358 ((wolf-hirschhorn$ or wolff hirschorn$ or chromosome deletion dillan$ or pitt-rogers-dank$ or pitt$) adj3 (syndrome$ or disease$ or disorder$)).ti,ab,id. (91)

359 exp Crying Cat Syndrome/ (72)

360 ((cri du chat$ or crying cat$ or 5p or lejeune$) adj3 (syndrome$ or disease$ or disorder$)).ti,ab,id. (102)

361 ((jacobsen$ or 11q deletion) adj5 (syndrome$ or disease$ or disorder$)).ti,ab,id. (7)

362 exp "Translocation (Chromosome)"/ or exp Trisomy/ or exp "Deletion (Chromosome)"/ (1244)

363 (9p minus or 9p deletion).ti,ab,id. (3)

364 (alfi$ adj (syndrome$ or disease$ or disorder$)).ti,ab,id. (0)

365 (degouchy$ or de gouchy$ or degrouchy$ or de grouchy$).ti,ab,id. (3)

366 distal 18q.ti,ab,id. (1)

367 (ondine$ curse or congenital central hypoventilation or primary alveolar hypoventilation).ti,ab,id. (35)

368 (((graft vs host or graft versus host) adj (disease$ or syndrome$ or disorder)) and chronic$).ti,ab,id. (36)

369 exp Congenital Disorders/ and (Aneurysms/ or Birth Injuries/ or Birth Trauma/ or Kidney Diseases/ or Rubella/) (39)

370 or/1-369 (117237)

371 exp HIV/ (43013)

372 (HIV or human immunodeficiency virus$).ti,ab,id. (52871)

373 (htlv or human t-lymphotropic virus$ or human t cell lymphotropic virus$).ti,ab,id. (187)

374 exp AIDS/ (15424)

375 (acquired immune deficiency syndrome$ or acquired immunodeficiency syndrome$).ti,ab,id. (3813)

376 (AIDS adj3 (virus$ or infection$)).ti,ab,id. (1208)

377 (AIDS adj (related or associated)).ti,ab,id. (1903)

378 exp Neoplasms/ (52062)

379 (cancer$ or carcin$ or tumor$ or tumour$ or neoplas$ or adenocarcin$ or oncol$ or malignan$).ti,ab,id. (82853)

380 exp Cystic Fibrosis/ (900)

381 (cystic fibrosis or fibrocystic or fibro-cystic or mucoviscidosis or cf).ti,ab,id. (4146)

382 exp Cerebral Palsy/ (5497)

383 (cerebr$ adj3 pals$).ti,ab,id. (7803)

384 exp Kidney Diseases/ (2187)

385 ((kidney$ or renal) adj3 (failure$ or insufficienc$)).ti,ab,id. (1542)

386 (end stage adj3 (kidney or renal)).ti,ab,id. (1156)

387 (("stage 5" or "stage V") adj3 (kidney or renal)).ti,ab,id. (10)

388 (ESRD or ESKD or ESRF or ESKF or CRF or CKF).ti,ab,id. (3453)

389 or/371-388 (158661)

390 exp Health Care Services/ (209101)

391 long term care/ or case management/ or home care/ or nursing homes/ (22064)

392 community services/ or community welfare services/ or home care/ or home visiting programs/ (26106)

393 hospitals/ or residential care institutions/ or clinics/ or hospital environment/ or intensive care/ (35991)

394 ("hospital at home" or "home adj2 visit* or home care or home-care or domiciliary care or house-call* or house adj2 call$").ti,ab,id. (41)

395 ((community or district or home) adj nursing).ti,ab,id. (605)

396 exp Primary Health Care/ (18318)

397 (family practice or GP practice or general practice or primary care).ti,ab,id. (35682)

398 exp Outpatient Treatment/ (6857)

399 (health centre$ or health center$ or health clinic$ or child$ clinic$ or outpatient clinic$ or out-patient clinic$).ti,ab,id. (23839)

400 exp Hospice/ (3252)

401 (hospital$ or hospice$ or nursing home$ or intermediate care or long-term care or long term care).ti,ab,id. (176643)

402 (inpatient$ ward$ or in-patient$ ward$ or inpatient facilit$ or in-patient$ facilit$).ti,ab,id. (1131)

403 exp Terminally Ill Patients/ (4722)

404 exp Palliative Care/ (14065)

405 exp Intensive Care/ (5729)

406 ((critical or "end of life" or EOL or intensive or palliative or terminal or hospice) adj2 care).ti,ab,id. (23825)

407 EOLC.ti,ab,id. (51)

408 or/390-407 (412492)

409 exp Health Personnel/ (162163)

410 exp medical personnel/ or exp physicians/ (81935)

411 exp nurses/ (31474)

412 ((clinical or health or hospice$ or medical or nursing) adj2 (personnel or practitioner$ or staff)).ti,ab,id. (24829)

413 (clinician$ or doctor$ or general practitioner$ or GP$ or nurse$ or physician$).ti,ab,id. (260227)

414 or/409-413 (363387)

415 exp Qualitative Methods/ (15500)

416 qualitativ$.ti,ab,id. (179056)

417 exp Interviews/ (14639)

418 interview$.ti,ab,id. (328640)

419 exp Focus Group/ (883)

420 focus group$.ti,ab,id. (35724)

421 exp Grounded Theory/ (3739)

422 (grounded theor$ or grounded study or grounded studies or grounded research or grounded analys$).ti,ab,id. (16617)

423 exp Phenomenology/ (14150)

424 phenomenol$.ti,ab,id. (43456)

425 exp Ethnography/ (8758)

426 (ethnograph$ or ethnonurs$ or ethno-graph$ or ethno-nurs$).ti,ab,id. (28258)

427 (story or stories or storytelling or narrative$ or narration$).ti,ab,id. (117004)

428 (open-ended or open question$ or text$).ti,ab,id. (131271)

429 exp Narratives/ (19980)

430 (discourse$ analys$ or discurs$ analys$).ti,ab,id. (7691)

431 content$ analys$.ti,ab,id. (26528)

432 ethnological.ti,ab,id. (359)

433 purposive sampl$.ti,ab,id. (5097)

434 (constant comparative or constant comparison$).ti,ab,id. (4849)

435 theoretical sampl$.ti,ab,id. (570)

436 (theme$ or thematic$).ti,ab,id. (135881)

437 (emic or etic or hermeneutic$ or heuristic$ or semiotic$).ti,ab,id. (27057)

438 data saturat$.ti,ab,id. (318)

439 participant observ$.ti,ab,id. (8876)

440 cluster sampl$.ti,ab,id. (1613)

441 (humanistic$ or existential$ or experiential$ or paradigm$).ti,ab,id. (135392)

442 (social construct$ or postmodern$ or post-modern$ or poststructural or post-structural$ or feminis$ or constructivis$).ti,ab,id. (46002)

443 (action research or cooperative inquir$ or co-operative inquir$).ti,ab,id. (8425)

444 human science.ti,ab,id. (608)

445 biographical method$.ti,ab,id. (102)

446 conversation$.ti,ab,id. (37193)

447 life world.ti,ab,id. (619)

448 theoretical saturation.ti,ab,id. (117)

449 group discussion$.ti,ab,id. (10024)

450 direct observation$.ti,ab,id. (4556)

451 mixed method$.ti,ab,id. (24689)

452 (observational method$ or observational approach$).ti,ab,id. (1200)

453 (life experience$ or personal experience$ or patient$ experience$ or lived experience$ or actual experience$ or real experience$ or "experience of patient$" or "experiences of patient$").ti,ab,id. (50631)

454 ((personal or patient$) adj account$).ti,ab,id. (2412)

455 ("account of patient$" or "accounts of patient$").ti,ab,id. (443)

456 ((personal or patient$) adj (perception$ or perspective$)).ti,ab,id. (7745)

457 ("perspective of patient$" or "perspectives of patient$" or "perception of patient$" or "perceptions of patient$").ti,ab,id. (2105)

458 ((personal or patient$) adj opinion$).ti,ab,id. (745)

459 ("opinion of patient$" or "opinions of patient$").ti,ab,id. (139)

460 (patient$ adj (need or needs or needed or needing or requir$)).ti,ab,id. (6157)

461 ("need of patient$" or "needs of patient$" or "requirement of patient$" or "requirements of patient$").ti,ab,id. (2279)

462 ((patient$ or personal) and (experience or experiences or perspective$ or perception$ or opinion$ or account or accounts or need or needs or needed or needing or requir$)).ti. (14654)

463 ((questionnaire$ or survey$) and patient$ and (experience or experiences or perspective$ or perception$ or opinion$ or account or accounts or need or needs or needed or needing or requir$)).ti,ab,id. (38509)

464 key informant$.ti,ab,id. (3418)

465 (field study or field studies or field research$ or field work$ or fieldwork$).ti,ab,id. (17941)

466 (semi-structured or semistructured or unstructured or un-structured or informal or in-depth or indepth).ti,ab,id. (125249)

467 "face-to-face".ti,ab,id. (22027)

468 ((guide or structured) adj5 (discussion$ or questionnaire$)).ti,ab,id. (8255)

469 (heidegger$ or colaizzi$ or speigelberg$ or van manen$ or van kaam$ or merleau ponty$ or husserl$ or giorgi$ or foucault$ or corbin$ or glaser$).ti,ab,id. (10358)

470 or/415-469 (1029465)

471 (370 or 389) and (408 or 414) and 470 (20383)

472 limit 471 to (100 childhood <birth to age 12 yrs> or 120 neonatal <birth to age 1 mo> or 140 infancy <2 to 23 mo> or 160 preschool age <age 2 to 5 yrs> or 180 school age <age 6 to 12 yrs> or 200 adolescence <age 13 to 17 yrs>) (2304)

473 ((pediatric or paediatric) adj3 (critical or EOL or EOLC or "end of life" or palliative or terminal)).ti,ab,id. (594)

474 ((child or children) adj3 (critical or EOL or EOLC or "end of life" or palliative or terminal)).ti,ab,id. (1333)

475 ((adolescen$ or teenager$ or young person$ or young people) adj3 (critical or EOL or EOLC or "end of life" or palliative or terminal)).ti,ab,id. (1016)

476 ((pediatric or paediatric) adj3 hospice).ti,ab,id. (65)

477 ((child or children) adj3 hospice).ti,ab,id. (44)

478 ((adolescen$ or teenager$ or young person$ or young people) adj3 hospice).ti,ab,id. (3)

479 ((pediatric or paediatric) adj3 ("life limiting" or life-limiting or LLC$)).ti,ab,id. (5)

480 ((pediatric or paediatric) adj3 ("life threatening" or life-threatening)).ti,ab,id. (19)

481 ((child$ or children$) adj3 (life-limiting or "life llimiting" or LLC$)).ti,ab,id. (122)

482 ((child$ or children$) adj3 ("life threatening" or life-threatening)).ti,ab,id. (268)

483 ((adolescen$ or teenager$ or young person$ or young people) adj3 (life-limiting or "life llimiting" or LLC$)).ti,ab,id. (29)

484 ((adolescen$ or teenager$ or young person$ or young people) adj3 ("life threatening" or life-threatening)).ti,ab,id. (38)

485 or/473-484 (3202)

486 485 and 470 (1046)

487 limit 486 to (100 childhood <birth to age 12 yrs> or 120 neonatal <birth to age 1 mo> or 140 infancy <2 to 23 mo> or 160 preschool age <age 2 to 5 yrs> or 180 school age <age 6 to 12 yrs> or 200 adolescence <age 13 to 17 yrs>) (586)

488 472 or 487 (2827)

489 limit 488 to yr="2010 -Current" (1731)

**CINAHL Complete via EBSCO**

Search date 11^th^ July 2020. Two searches were carried out: search one identified 5596 records and search two identified 7037 records

EBSCO’s limit of 8000 words in search strategies required the search to be split into two separate searches. Search one was: [specific conditions] AND [settings OR healthcare staff] AND [qualitative research] AND [children/parents] and search two was: [generic terms for life-limiting conditions] AND [qualitative research]. In search one the number of search terms used to describe specific conditions was reduced as compared to the search strategies used for the MEDLINE and PsycINFO databases.

**CINAHL Complete Search Strategy One**

S1 ( (MH "Human Immunodeficiency Virus+") OR (MH "HIV Infections+") OR (MM "Acquired Immunodeficiency Syndrome") ) OR TI ( HIV or "human immunodeficiency virus" ) OR AB ( HIV or "human immunodeficiency virus" ) OR TI ( htlv or "human t-lymphotropic virus*" or "human t cell lymphotropic virus*" ) OR AB ( htlv or "human t-lymphotropic virus*" or "human t cell lymphotropic virus*" ) OR TI ( "acquired immune deficiency syndrome*" or "acquired immunodeficiency syndrome*" ) OR AB ( "acquired immune deficiency syndrome*" or "acquired immunodeficiency syndrome*" ) OR TI ( AIDS N3 (virus* or infection*) ) OR AB ( AIDS N3 (virus* or infection*) ) OR TI ( AIDS N1 (related or associated) ) OR AB ( AIDS N1 (related or associated) ) Show Less

S2 (MH "Neoplasms+") OR TI ( (cancer* or carcin* or tumor* or tumour* or neoplas* or adenocarcin* or oncol* or malignan*) ) OR AB ( (cancer* or carcin* or tumor* or tumour* or neoplas* or adenocarcin* or oncol* or malignan*) )

S3 (MH "Cystic Fibrosis") OR TI ( "cystic fibrosis" or fibrocystic or fibro-cystic or mucoviscidosis or cf ) OR AB ( "cystic fibrosis" or fibrocystic or fibro-cystic or mucoviscidosis or cf )

S4 (MH "Cerebral Palsy") OR TI cerebr* N3 pals* OR TI cerebr* N3 pals*

S5 ( (MH "Muscle Spasticity") OR (MH "Quadriplegia") ) OR TI spasticit* OR AB spasticit* OR TI ( spastic* and (quadripleg* or tetrapleg*) ) OR AB ( spastic* and (quadripleg* or tetrapleg*) )

S6 (MH "Renal Insufficiency") OR TI ( (kidney* or renal) N3 (failure* or insufficienc*) ) OR AB ( (kidney* or renal) N3 (failure* or insufficienc*) ) OR TI ( "end stage" N3 (kidney or renal) ) OR AB ( "end stage" N3 (kidney or renal) ) OR TI ( ("stage 5" or "stage V") N3 (kidney or renal) ) OR AB ( ("stage 5" or "stage V") N3 (kidney or renal) ) OR TI ( ESRD or ESKD or ESRF or ESKF or CRF or CKF ) OR AB ( ESRD or ESKD or ESRF or ESKF or CRF or CKF )

S7 S1 OR S2 OR S3 OR S4 OR S5 OR S6

S8 ( (MH "Health Services+") OR (MH "Child Health Services+") OR (MH "Community Health Services+") OR (MH "Emergency Medical Services+") ) OR ( (MH "Community Health Nursing") OR (MH "Hospice and Palliative Nursing") OR (MH "Emergency Nursing") OR (MH "Psychiatric Nursing") OR (MH "Pediatric Oncology Nursing") OR (MH "Pediatric Critical Care Nursing") OR (MH "Perinatal Nursing") OR (MH "Oncologic Nursing") OR (MH "Occupational Health Nursing") OR (MH "Neuroscience Nursing") OR (MH "Holistic Nursing") OR (MH "Critical Care Nursing") OR (MH "Rural Health Nursing") OR (MH "Respiratory Nursing") OR (MH "Anesthesia Nursing") OR (MH "Primary Nursing") OR (MH "Pediatric Nursing") OR (MH "Orthopedic Nursing") OR (MH "Neonatal Nursing") OR (MH "Home Nursing, Professional") OR (MH "Gastroenterology Nursing") OR (MH "Ambulatory Care Nursing") OR (MH "Team Nursing") OR (MH "School Health Nursing") OR (MH "Rehabilitation Nursing") OR (MH "Ophthalmic Nursing") OR (MH "Nursing Care") OR (MH "Home Nursing") OR (MH "Genetics Nursing") OR (MH "Cardiovascular Nursing") ) OR ( TI ( "hospital at home" or "home N2 visit* or "home care" or home-care or "domiciliary care" or house-call* or "house N2 call*" ) OR AB ( "hospital at home" or "home N2 visit* or "home care" or home-care or "domiciliary care" or house-call* or "house N2 call*" ) OR TI ( (community or district or home) N1 nursing ) OR AB ( (community or district or home) N1 nursing ) ) OR ( (MH "Primary Health Care") OR (MH "Physicians, Family") ) OR ( TI ( "family practice" or "GP practice" or "general practice" or "primary care" ) OR AB ( "family practice" or "GP practice" or "general practice" or "primary care" ) ) OR ( (MH "Community Health Centers") OR (MH "Rural Health Centers") OR (MH "Hospitals, Community") OR (MH "Ambulatory Care Facilities") OR (MH "Outpatients") OR (MH "Outpatient Service") ) Show Less

S9 ( (MH "Community Health Centers") OR (MH "Rural Health Centers") OR (MH "Hospitals, Community") OR (MH "Ambulatory Care Facilities") OR (MH "Outpatients") OR (MH "Outpatient Service") ) OR ( TI ( "health centre*" or "health center*" or "health clinic*" or "child* clinic*" or "outpatient clinic*" or "out-patient clinic*" ) OR AB ( "health centre*" or "health center*" or "health clinic*" or "child* clinic*" or "outpatient clinic*" or "out-patient clinic*" ) OR TI ( hospital* or hospice* or "nursing home*" or "intermediate care" or "long-term care" or "long term care" ) OR AB ( hospital* or hospice* or "nursing home*" or "intermediate care" or "long-term care" or "long term care" ) OR TI ( "inpatient* ward*" or "in-patient* ward*" or "inpatient facilit*" or "in-patient* facilit*" ) OR ( "inpatient* ward*" or "in-patient* ward*" or "inpatient facilit*" or "in-patient* facilit*" ) ) OR ( (MH "Terminal Care") OR (MH "Palliative Care") OR (MH "Hospice Care") OR (MH "Critical Care Nursing") OR (MH "Critical Care") OR (MM "Intensive Care Units+") OR (MH "Intensive Care Units, Pediatric+") ) OR ( TI ( (critical or "end of life" or EOL or intensive or palliative or terminal or hospice) N2 care ) OR AB ( (critical or "end of life" or EOL or intensive or palliative or terminal or hospice) N2 care ) OR TX EOLC ) Show Less

S10 S8 OR S9

S11 ( (MH "Medical Staff") OR (MH "Physical Therapist Assistants") OR (MH "Nursing Assistants") OR (MH "Home Health Aides") OR (MH "Community Health Workers") OR (MH "Allied Health Personnel+") OR (MH "Health Personnel+") ) OR ( (MH "Physicians+") OR ( (MH "Nurses by Educational Level+") OR (MH "Nurses by Specialty+") OR (MH "Nurses, Other+") OR (MH "Nurses+") ) ) OR TI ( doctor* or physician* or "general practitioner*" or "medical practitioner*" ) OR TI ( nurse* or "nursing practitioner*" or "nursing staff" or "hospice staff" ) OR AB ( doctor* or physician* or "general practitioner*" or "medical practitioner*" ) OR AB ( nurse* or "nursing practitioner*" or "nursing staff" or "hospice staff" )( (MH "Medical Staff") OR (MH "Physical Therapist Assistants") OR (MH "Nursing Assistants") OR (MH "Home Health Aides") OR (MH "Community Health Workers") OR (MH "Allied Health Personnel+") OR (MH "Health Personnel+") ) OR ( (MH "Physicians+") OR ( (MH "Nurses by Educational Level+") OR (MH "Nurses by Specialty+") OR (MH "Nurses, Other+") OR (MH "Nurses+") ) ) OR TI ( doctor* or physician* or "general practitioner*" or "medical practitioner*" ) OR TI ( nurse* or "nursing practitioner*" or "nursing staff" or "hospice staff" ) OR AB ( doctor* or physician* or "general practitioner*" or "medical practitioner*" ) OR AB ( nurse* or "nursing practitioner*" or "nursing staff" or "hospice staff" ) Show Less

S12 (MH "Qualitative Studies") OR (MH "Interviews+") OR (MH "Focus Groups") OR (MH "Narratives")

S13 TI ( qualitativ* or interview* or "focus group*" ) OR AB ( qualitativ* or interview* or "focus group*" ) OR TI ( "grounded theor*" or "grounded stud*" or "grounded research" or "grounded analys*" ) OR AB ( "grounded theor*" or "grounded stud*" or "grounded research" or "grounded analys*" ) OR TI ( phenomenol* or ethnograph* or ethnonurs* or ethno-graph* or ethno-nurs* ) OR AB ( phenomenol* or ethnograph* or ethnonurs* or ethno-graph* or ethno-nurs* ) OR ( story or stories or storytelling or narrative* or narration* or "open-ended" or "open question*" or text ) OR ( story or stories or storytelling or narrative* or narration* or "open-ended" or "open question*" or text ) OR ( "discourse* analys*" or "discurs* analys*" or "content analys*" or ethnological ) OR ( "discourse* analys*" or "discurs* analys*" or "content analys*" or ethnological ) Show Less

S14 TI ( Patient* N1 (need or needs or needed or needing or requir*) ) OR AB ( Patient* N1 (need or needs or needed or needing or requir*) ) OR TI ( "need of patient*" or "needs of patient*" or "requirement of patient*" or "requirements of patient*" ) OR AB ( "need of patient*" or "needs of patient*" or "requirement of patient*" or "requirements of patient*" ) OR TI ( ((patient* or personal) and (experience or experiences or perspective* or perception* or opinion* or account or accounts or need or needs or needed or needing or requir*)) ) OR TI ( (survey*) and patient* and (experience or experiences or perspective* or perception* or opinion* or account or accounts or need or needs or needed or needing or requir*) ) OR AB ( (survey*) and patient* and (experience or experiences or perspective* or perception* or opinion* or account or accounts or need or needs or needed or needing or requir*) ) OR TI "key informant*" OR AB "key informant*" OR TI ( "field study" or "field studies" or "field research*" or "field work*" or fieldwork* ) OR AB ( "field study" or "field studies" or "field research*" or "field work*" or fieldwork* ) Show Less

S15 TI ( "purposive sampl*" or "constant comparative" or "constant comparison*" ) OR AB ( "purposive sampl*" or "constant comparative" or "constant comparison*" ) OR TI ( "theoretical sampl*" or theme* or thematic* ) OR AB ( "theoretical sampl*" or theme* or thematic* ) OR TI ( emic or etic or hermeneutic* or heuristic* or semiotic* ) OR AB ( emic or etic or hermeneutic* or heuristic* or semiotic* ) OR TI ( "data saturat*" or "particpant observ*" or "cluster sampl*" ) OR AB ( "data saturat*" or "particpant observ*" or "cluster sampl*" ) OR TI ( humanistic* or existential* or experiential* or paradigm* ) OR AB ( humanistic* or existential* or experiential* or paradigm* ) Show Less

S16 TI ( "social construct*" or postmodern* or post-modern* or poststructural or post-structural* or feminis* or constructivis* ) OR AB ( "social construct*" or postmodern* or post-modern* or poststructural or post-structural* or feminis* or constructivis* ) OR TI ( "action research" or "cooperative inquir*" or "co-operative inquir*" or "human science" ) OR AB ( "action research" or "cooperative inquir*" or "co-operative inquir*" or "human science" ) OR TI ( "biographical method*" or conversation* or "life world" or "theoretical saturation" ) OR AB ( "biographical method*" or conversation* or "life world" or "theoretical saturation" ) OR TI ( "group discussion*" or "mixed method*") OR AB ( "group discussion*" or "mixed method*") OR TI ( "life experience*" or "personal experience*" or "patient* experience*" or "lived experience*" or "actual experience*" or "real experience*" or "experience of patient*" or "experiences of patient*" ) OR AB ( "life experience*" or "personal experience*" or "patient* experience*" or "lived experience*" or "actual experience*" or "real experience*" or "experience of patient*" or TI ( (personal or patient*) N1 account* ) OR AB ( (personal or patient*) N1 account* ) OR TI ( "account of patient*" or "accounts of patient*" ) OR AB ( "account of patient*" or "accounts of patient*" ) OR TI ( (personal or patient*) N1 (perception* or perspective*) ) OR AB ( (personal or patient*) N1 (perception* or perspective*) ) OR TI ( "perspective of patient*" or "perspectives of patient*" or "perception of patient*" or "perceptions of patient*" ) OR AB ( "perspective of patient*" or "perspectives of patient*" or "perception of patient*" or "perceptions of patient*" ) OR TI ( (personal or patient*) N1 opinion* ) OR AB ( (personal or patient*) N1 opinion* ) OR TI ( "opinion of patient*" or "opinions of patient*" ) OR AB ( "opinion of patient*" or "opinions of patient*" ) Show Less

S17 TI ( semi-structured or semistructured or unstructured or un-structured or informal or in-depth or indepth or "face-to-face" ) OR AB ( semi-structured or semistructured or unstructured or un-structured or informal or in-depth or indepth or "face-to-face" ) OR TI ( (guide or structured) N5 (discussion*) ) OR AB ( (guide or structured) N5 (discussion*) ) OR TI ( heidegger* or colaizzi* or speigelberg* or "van manen*" or "van kaam*" or "merleau ponty*" or husserl* or giorgi* or foucault* or corbin* or glaser* ) OR AB ( heidegger* or colaizzi* or speigelberg* or "van manen*" or "van kaam*" or "merleau ponty*" or husserl* or giorgi* or foucault* or corbin* or glaser* ) Show Less

S18 S12 OR S13 OR S14 OR S15 OR S16 OR S17

S19 (MH "Child+") OR (MH "Adolescence+")

S20 (MH "Parents+")

S21 TI ( child* or children* or adolescen* or parent* or parents* or brother* or sister* or sibling* or teenager* or "young person*" or "young people" ) OR AB ( child* or children* or adolescen* or parent* or parents* or brother* or sister* or sibling* or teenager* or "young person*" or "young people" )

S22 S19 OR S20 OR S21

S23 S7 AND (S10 OR S11) AND S18 AND S22

Limiters - Published Date: 20100101-20201231

**CINAHL Search Strategy Two**

S1 ( (pediatric or paediatric) N2 (critical or EOL or EOLC or "end of life" or palliative or terminal) ) OR ( (child or children) N2 (critical or EOL or EOLC or "end of life" or palliative or terminal) ) OR ( (adolescen* or teenager* or young person* or young people) N2 (critical or EOL or EOLC or "end of life" or palliative or terminal) ) OR ( (pediatric or paediatric) N2 hospice ) OR ( child or children) N2 hospice ) OR ( (adolescen* or teenager* or young person* or young people) N2 hospice ) OR ( (pediatric or paediatric) N2 ("life limiting" or life-limiting or LLC*) ) OR ( (pediatric or paediatric) N2 ("life threatening" or life-threatening) ) Show Less

S2 ( (child* or children*) N2 (life-limiting or "life limiting" or LLC*) ) OR ( (child* or children*) N2 ("life threatening" or life-threatening)) ) OR ( (adolescen* or teenager* or young person* or young people) N2 (life-limiting or "life llimiting" or LLC*) ) OR ( (adolescen* or teenager* or young person* or young people) N2 ("life threatening" or life-threatening) )

S3 S1 OR S2

S4 (MH "Qualitative Studies")

S5 TI ( (personal or patient*) N1 account* ) OR AB ( (personal or patient*) N1 account* ) OR TI ( "account of patient*" or "accounts of patient*" ) OR AB ( "account of patient*" or "accounts of patient*" ) OR TI ( (personal or patient*) N1 (perception* or perspective*) ) OR AB ( (personal or patient*) N1 (perception* or perspective*) ) OR TI ( "perspective of patient*" or "perspectives of patient*" or "perception of patient*" or "perceptions of patient*" ) OR AB ( "perspective of patient*" or "perspectives of patient*" or "perception of patient*" or "perceptions of patient*" ) OR TI ( (personal or patient*) N1 opinion* ) OR AB ( (personal or patient*) N1 opinion* ) OR TI ( "opinion of patient*" or "opinions of patient*" ) OR AB ( "opinion of patient*" or "opinions of patient*" ) Show Less

S6 TI ( semi-structured or semistructured or unstructured or un-structured or informal or in-depth or indepth or "face-to-face" ) OR AB ( semi-structured or semistructured or unstructured or un-structured or informal or in-depth or indepth or "face-to-face" ) OR TI ( (guide or structured) N5 (discussion* or questionnaire*) ) OR AB ( (guide or structured) N5 (discussion* or questionnaire*) ) OR TI ( heidegger* or colaizzi* or speigelberg* or "van manen*" or "van kaam*" or "merleau ponty*" or husserl* or giorgi* or foucault* or corbin* or glaser* ) OR AB ( heidegger* or colaizzi* or speigelberg* or "van manen*" or "van kaam*" or "merleau ponty*" or husserl* or giorgi* or foucault* or corbin* or glaser* ) Show Less

S7 (MH "Interviews+")

S8 (MH "Focus Groups")

9 (MH "Narratives")

S10 TI ( Patient* N1 (need or needs or needed or needing or requir*) ) OR AB ( Patient* N1 (need or needs or needed or needing or requir*) ) OR TI ( "need of patient*" or "needs of patient*" or "requirement of patient*" or "requirements of patient*" ) OR AB ( "need of patient*" or "needs of patient*" or "requirement of patient*" or "requirements of patient*" ) OR TI ( ((patient* or personal) and (experience or experiences or perspective* or perception* or opinion* or account or accounts or need or needs or needed or needing or requir*)) ) OR TI ( (survey*) and patient* and (experience or experiences or perspective* or perception* or opinion* or account or accounts or need or needs or needed or needing or requir*) ) OR AB ( (survey*) and patient* and (experience or experiences or perspective* or perception* or opinion* or account or accounts or need or needs or needed or needing or requir*) ) OR TI "key informant*" OR AB "key informant*" OR TI ( "field study" or "field studies" or "field research*" or "field work*" or fieldwork* ) OR AB ( "field study" or "field studies" or "field research*" or "field work*" or fieldwork* ) Show Less

S11 TI ( qualitativ* or interview* or "focus group*" ) OR AB ( qualitativ* or interview* or "focus group*" ) OR TI ( "grounded theor*" or "grounded stud*" or "grounded research" or "grounded analys*" ) OR AB ( "grounded theor*" or "grounded stud*" or "grounded research" or "grounded analys*" ) OR TI ( phenomenol* or ethnograph* or ethnonurs* or ethno-graph* or ethno-nurs* ) OR AB ( phenomenol* or ethnograph* or ethnonurs* or ethno-graph* or ethno-nurs* ) OR ( story or stories or storytelling or narrative* or narration* or "open-ended" or "open question*" or text ) OR ( story or stories or storytelling or narrative* or narration* or "open-ended" or "open question*" or text ) OR ( "discourse* analys*" or "discurs* analys*" or "content analys*" or ethnological ) OR ( "discourse* analys*" or "discurs* analys*" or "content analys*" or ethnological ) Show Less

S12 TI ( "purposive sampl*" or "constant comparative" or "constant comparison*" ) OR AB ( "purposive sampl*" or "constant comparative" or "constant comparison*" ) OR TI ( "theoretical sampl*" or theme* or thematic* ) OR AB ( "theoretical sampl*" or theme* or thematic* ) OR TI ( emic or etic or hermeneutic* or heuristic* or semiotic* ) OR AB ( emic or etic or hermeneutic* or heuristic* or semiotic* ) OR TI ( "data saturat*" or "particpant observ*" or "cluster sampl*" ) OR AB ( "data saturat*" or "particpant observ*" or "cluster sampl*" ) OR TI ( humanistic* or existential* or experiential* or paradigm* ) OR AB ( humanistic* or existential* or experiential* or paradigm* ) Show Less

S13 TI ( "social construct*" or postmodern* or post-modern* or poststructural or post-structural* or feminis* or constructivis* ) OR AB ( "social construct*" or postmodern* or post-modern* or poststructural or post-structural* or feminis* or constructivis* ) OR TI ( "action research" or "cooperative inquir*" or "co-operative inquir*" or "human science" ) OR AB ( "action research" or "cooperative inquir*" or "co-operative inquir*" or "human science" ) OR TI ( "biographical method*" or conversation* or "life world" or "theoretical saturation" ) OR AB ( "biographical method*" or conversation* or "life world" or "theoretical saturation" ) OR TI ( "group discussion*" or "mixed method*") OR AB ( "group discussion*" or "mixed method*") OR TI ( "life experience*" or "personal experience*" or "patient* experience*" or "lived experience*" or "actual experience*" or "real experience*" or "experience of patient*" or "experiences of patient*" ) OR AB ( "life experience*" or "personal experience*" or "patient* experience*" or "lived experience*" or "actual experience*" or "real experience*" or "experience of patient*" or "experiences of patient*" ) Show Less

S14 S4 OR S5 OR S6 OR S7 OR S8 OR S9 OR S10 OR S11 OR S12 OR S13

S15 S3 AND S14

Limiters - Published Date: 20100101-20201231
