# Supplementary material for: Hospital healthcare experiences of children and young people with life-threatening or life-shortening conditions, and their parents: scoping reviews and resultant conceptual frameworks
Source: BMC Pediatr. 2023 Jul 17;23:366. doi: 10.1186/s12887-023-04151-6 (PMC10351142; doi:10.1186/s12887-023-04151-6)
Supplement: Supplementary file 2 — Additional file 2: SupplementaryFile 2. Final versions of data extraction frameworks. [file 12887_2023_4151_MOESM2_ESM.docx]

**Supplementary File 2:**

**Final versions of data extraction frameworks**

**Table 1: Thematic framework for Review 1 - Aspects of health service delivery and care experienced that matter to children & young people**

| **Themes:**   1. Continuity in health care staff 2. Healthcare staff respond to requests for help 3. Treatments and medications are provided on time 4. Staff being knowledgeable about the CYPs condition and its management 5. Being skilled in performing medical procedures 6. Being calm when providing clinical or personal 7. Understanding the CYP’s non-verbal communication 8. Care is thorough and careful 9. Minimising physical suffering 10. Explaining and preparing CYP for treatment and procedures 11. Offering CYP choice and control over what they are told about their health 12. Empathetic delivery of medical information 13. Clear and understandable medical information 14. Encouraging CYP to be hopeful during active treatment 15. Offering choice and control over involvement in decisions that impact on health outcomes) 16. Being consulted about how clinical procedures are undertaken and care is provided 17. Kind behaviour 18. Treating the CYP as an individual 19. Taking CYP’s wider life into account when organising medical care and treatment 20. Respectful personal care 21. Time and effort invested in caring for the CYP 22. Responding to the CYP’s emotional needs 23. Responding to CYP’s play and/or social needs 24. Facilities to enable the CYP to be physically close to parent 25. Responding to the CYPs need for privacy 26. A comfortable ward environment 27. An accessible ward environment 28. Food that is pleasant |
| --- |

**Table 2: Thematic framework for Review 2 – Aspects of health service delivery and care experienced that matter to parents**

| **Themes:**   1. Continuity in health care staff 2. Coordination of care 3. Staff take information and advice from other specialisms into account 4. Communication between staff on the ward/unit about the CYP’s treatment/care 5. Agreement between staff about child’s treatment and care plan 6. Parent’s access to health care staff 7. Responding to the parent’s health/medical information needs 8. Preparing parents for the changes they will see in their child 9. Being willing to answer questions 10. Giving parents information on how the unit/ward works 11. Health/medical information paced according to parent’s needs 12. Keeping parents updated on changes in their child’s condition, treatment & care 13. Clear and understandable health/medical information 14. Empathetic communication of health/medical information 15. Delivering bad news in private 16. Open and honest communication of health/medical information 17. Staff who are positive 18. Being able to question/challenge professional opinion 19. Being given a choice over involvement in making significant decisions 20. Respecting the parent’s knowledge and expertise on their child and their health 21. Acknowledging the impact of the situation on family 22. Being kind 23. Comforting parents 24. Talking to parents about life outside the hospital 25. Taking an interest in and get to know the family 26. Taking family life into account when organising medical care and treatment 27. Supporting involvement in personal care of the CYP (i.e. washing, feeding, toileting) 28. Supporting involvement in medical/health care of the child 29. Parents having time with their child 30. Parent being physically close to the CYP 31. Advice on welfare benefits 32. Recognising and responding to parent’s physical health needs 33. Checking how parent is feeling 34. Access to psychological support 35. Recognising and responding to parents need for privacy with CYP 36. Spiritual, religious and cultural needs 37. Providing opportunities to meet other parents 38. Consulting the parent before talking to the child about their health 39. Supporting parents with talking to their child about their health 40. Supporting parents was talking to siblings about the child’s health 41. Toilets for parents on wards 42. Rooms for parents who need a break 43. Access to interpreters 44. Staff were calm 45. Staff were polite 46. Staff are committed to caring for the child 47. Treating the CYP like they matter as much as any other CYP 48. Staff going above and beyond 49. Staff who are emotional about the situation the child and family are experiencing |
| --- |
