# Supplementary material for: Hospital healthcare experiences of children and young people with life-threatening or life-shortening conditions, and their parents: scoping reviews and resultant conceptual frameworks
Source: BMC Pediatr. 2023 Jul 17;23:366. doi: 10.1186/s12887-023-04151-6 (PMC10351142; doi:10.1186/s12887-023-04151-6)
Supplement: Supplementary file 3 — Additional file 3: Supplementary File 3. Extract from a thematic table used for main analysis. [file 12887_2023_4151_MOESM3_ESM.docx]

**Supplementary File 3:**

**Extract from a thematic table used for main analysis**

| **Review 1: Theme 11 – Staff explain what is going to happen to the CYP** | | | | |
| --- | --- | --- | --- | --- |
| **Total number of studies: 9** | | | | |
| **Paper ID:** 23, 62, (65*,66), 108, 123, 6, 16, 24, 54 | | | | |
| **Data source (Parent, CYP or both):** both | | | | |
| **Staff characteristics/actions or organisational features/facilities?:** Staff action | | | | |
| **Definition:** Staff offer to speak directly to CYP about any medical treatments or procedures they are due to undergo (including any sensations they might experience) and (where appropriate) the self-management of medication/treatment. Information about medical treatments and procedures is offered in advance of them being undertaken so that CYP have time to prepare for the experience. | | | | |
| **Consequences for the CYP/ own view**   - CYP who had treatments and procedures explained to them perceived staff as being ‘kind’ and said it prevented shocks and surprises. - When CYP did *not* get information about treatments and procedures which they then had to undergo they reported feeling: confused, frightening, shocked and surprised, traumatised, overlooked, excluded and betrayed. - When given information at the last minute, they did not have time to mentally prepare for what was going to happen next | | | | |
| **Consequences for the CYP/parents’ views**  When information was given parents felt it signalled respect for the child and that staff took them seriously as people. It also prevented information being concealed from the CYP. | | | | |
| **Consequences for the parent:**  When staff provided CYP with information, parents trusted them.  When information was withheld from the CYP, some parents were uncomfortable feeling it was futile as the CYP knew what was going on anyway | | | | |
| **Paper ID** | **Extracts** | **Staff characteristics or staff actions/behaviour directed at patient/family** | **Organisational features/facilities** | **Consequences** |
| 23 | (During diagnosis and induction therapy) children and parents described their need for knowledge about procedures, to be able to understand their new situation, and its effect on everyday life. Parent quote: “There is a lot of information shortage . . . I think information is very, very important for both children and parents for their well-being” | Staff give information on any procedures to be undertaken |  | **For CYP - parent’s view:**  It’s good for well-being  **For parents:**  It’s good for well-being |
| 62 | CYP quote: “You don’t know why you’re there. You just kind of are in pain. People are just prodding you. You have no idea why. So I think that in itself is quite confusing in a traumatising sort of way... I was very very fearful... I developed a very very severe fear to injections... I think it’s got a lot of subconscious memories and some that have been quite severely suppressed” (female Neuroblastoma survivor, aged 1 at diagnosis). | Staff carry out procedures and provide treatment without explaining why |  | **For CYP - own views:**  When you don’t get information about what is going on it’s confusing, frightening and traumatising. |
