# Supplementary material for: Hospital healthcare experiences of children and young people with life-threatening or life-shortening conditions, and their parents: scoping reviews and resultant conceptual frameworks
Source: BMC Pediatr. 2023 Jul 17;23:366. doi: 10.1186/s12887-023-04151-6 (PMC10351142; doi:10.1186/s12887-023-04151-6)
Supplement: Supplementary file 4 — Additional file 4: Supplementary File 4. The impacts of health service delivery and care on children and young people - List of codes and their definitions. [file 12887_2023_4151_MOESM4_ESM.docx]

**Supplementary File 4:**

**The impacts of health service delivery and care on children and young people - List of codes and their definition**

| **Impact code** | **Definition** |
| --- | --- |
| Emotional wellbeing | Any statements about the impact of health service delivery and care on children’s emotional wellbeing, either positively (e.g. relaxed; happier; comforted etc.) or negatively (e.g. bored; bothered; distressed; panicked; frustrated; frightened; lonely; sad; shocked; traumatised etc.), or on ability to cope with the situation being faced. |
| Physical wellbeing | Any statements about the impact of health service delivery and care on children’s physical wellbeing, either positively or negatively (e.g. experienced pain; was unable to sleep etc.) |
| Trust in staff | Any statement about the impact that health service delivery and care on children’s trust or distrust in staff (e.g. felt safe;, like staff were making the correct decisions versus like staff might make a mistake; felt betrayed by staff etc.) |
| Feeling (un) comfortable with staff | Any statements about the impact of health service delivery and care on the extent to which children felt comfortable or uncomfortable with staff (e.g. felt comfortable with staff; intimidated by staff etc.) |
| Understanding of the situation being faced | Any statements about the impact of health service delivery and care on children’s understanding of and preparedness for the situation they were facing (e.g. feeling confused; unprepared etc.) |
| Empowerment & control | Any statements about the impact of health service delivery and care on children’s sense of power and control over the situation they were facing (e.g. feeling powerless; overlooked; not in control; ignored; that no one cared what they thought etc.) |
| At ease with (versus regretting) treatment decisions | Any statements about the impact of health service delivery and care on children’s satisfaction or dissatisfaction with treatment decisions, including feelings of regret (e.g. children had treatment they did not want and later regretted etc.) |
