# Supplementary material for: Hospital healthcare experiences of children and young people with life-threatening or life-shortening conditions, and their parents: scoping reviews and resultant conceptual frameworks
Source: BMC Pediatr. 2023 Jul 17;23:366. doi: 10.1186/s12887-023-04151-6 (PMC10351142; doi:10.1186/s12887-023-04151-6)
Supplement: Supplementary file 5 — Additional file 5: Supplementary File 5. The impacts of health service delivery and care on parents- List of codes and their definitions. [file 12887_2023_4151_MOESM5_ESM.docx]

**Supplementary File 5:**

**The impacts of health service delivery and care on parents- List of codes and their definition**

| **Impact code** | **Definition** |
| --- | --- |
| Emotional wellbeing | Any statements about the impact of health service delivery and care on emotional wellbeing, either positively (e.g. they were calm; relaxed; had a sense of peace etc.) or negatively (e.g. feeling alarmed; angry; anxious; depressed; distressed; frightened; frustrated; overwhelmed; scared; shocked etc.), or comments on ability to cope emotionally with the situation being faced (e.g. it helped parents to deal with the trauma they were experiencing; they were better able to cope with the stressors being experienced etc.) |
| Able to parent in the way they want | Any statements about the impact of health service delivery and care on the extent to which parents were able to parent, care for, or spend time with their child in the way they wanted (e.g. it maximized the amount of time parents could spend caring for their child, it allowed parents to get more involved in the care of their child; it inhibited parents ability to protect their child; it meant parents had to spend time away from their child; they felt their child was not their own etc.) |
| Trust in staff | Any statements about the impact of health service delivery and care on parents’ trust (or distrust) of staff, or the extent to which they and/or their child were safe under their care (e.g. parents had confidence in staff; felt their child was safe; felt their child was receiving care from the right team etc.) |
| Care burden | Any statements about the impact of health service delivery and care on the extent to which parents were burdened (or not) by providing medical care/treatments to r, or organizing the medical care/treatments for, their child (e.g. it made life easier as parents did not have to deal with so many people; they also had the burden of coordinating communicating; parents were expected to take on medical care, rather than being giving the option, and some felt the responsibility was too much etc.) |
| Empowerment & control | Any statements about the impact of health service and care had on parents’ sense of empowerment and control over, or confidence in dealing with, the situation they were facing (e.g. it helped parents feel confident; they opted to handover decision making to staff; they did not know what to do; they spoke of feeling helpless, as if they had no control of over the situation etc.) |
| Understanding of situation being faced | Any statements about the impact of health service delivery and care on parents’ understanding of the child’s health, treatment or care (e.g. parents experienced confusion regarding the care plan; they were unable to process the information given; they did not understand the child’s disease, care or complications etc.) |
| Feeling in partnership with staff (versus excluded byi or in conflict with) | Any statements about health service delivery and care impacted on how parents described their relationship with staff (e.g. they felt like part of the team; they felt welcomed, respected, valued; ‘they felt they were not alone, but instead were respected, care for and supported; they inspired a sense of collaboration, conflicts arose between parents and staff; they experienced feelings of abandonment; they felt they were an inconvenience to the team and rejected by them etc.). |
| Maintenance of usual family routine | Any statements about the impact of health service delivery and care on parents’ ability to maintain usual family routines (e.g. it allowed for stability in family life; it helped them maintain some aspects of normal life etc.) |
| At ease with (versus regretting) treatment and care decisions | Any statements about the impact of health service delivery and care on the extent to which parents were at ease with, versus uncomfortable with or regretful of the decisions made about the child’s treatment and care (e.g. parents had a negative appraisal of the final decision and some spoke of how their child received care that the parent later regretted; parents expressed regret as to how they had spent their time in the lead up to the child’s death [due to treatment decisions] etc.) |
| Satisfaction with child’s treatment & care | Any statements about the impact of health service delivery and care on parents’ satisfaction with treatment and care (e.g. it reduced their general satisfaction with the care provided; they felt staff had done all they could to help etc.) |
| Sense of hope | Any statements about the impact of health service delivery and care on parents’ sense of hope (e.g. it allowed parents to remain hopeful etc.) |
| Physical wellbeing | Any statements about the impact of health service delivery and care on parents’ physical wellbeing (e.g. ‘”it was exhausting”’ etc.) |
