# Supplementary material for: Hospital healthcare experiences of children and young people with life-threatening or life-shortening conditions, and their parents: scoping reviews and resultant conceptual frameworks
Source: BMC Pediatr. 2023 Jul 17;23:366. doi: 10.1186/s12887-023-04151-6 (PMC10351142; doi:10.1186/s12887-023-04151-6)
Supplement: Supplementary file 6 — Additional file 6: Supplementary File 6. Additional study characteristics. [file 12887_2023_4151_MOESM6_ESM.docx]

**Supplementary File 6:**

**Additional study characteristics**

| **Author**  **(Year) (Reference)** | **Review** | **All reported details of condition/diagnosis** | **Child sample:**  **Ethnicity reported** | **Parent sample:**  **Ethnicity reported** | **Review 2: No. children/young people represented (no. deceased)** |
| --- | --- | --- | --- | --- | --- |
| Anderson et al (2018)(27) | 1,2 | Neutropenia: confirmed or suspected | n/a | Not reported | n=9 |
| Angstrom -Brannstrom et al (2014)(28) | 1 | Cancer: solid tumours and leukaemia | Not reported | n/a | n/a |
| Baenzinger et al (2020)(29) | 1,2 | Cancer: solid tumours and leukaemia | n/a | Not reported | n=51 |
| Bergviken & Nilsson (2019)(30) | 2 | Cancer: not reported | n/a | Not reported | n=15 |
| Bailey-Pearce et al (2018)(31) | 2 | Not reported | n/a | Not reported | n=18 |
| Baird et al (2015, 2016)(32, 33) | 1,2 | Mixed: gastrointestinal, genetic disorders, seizure disorder, heart disease | n/a | 71. 4% Hispanic  28.6% non-Hispanic | n=7 |
| Ballantyne et al (2019)(34) | 2 | Cerebral palsy | n/a | Not reported | n=16 |
| Baugham et al (2017)(35) | 2 | Mixed: respiratory and congenital conditions | n/a | n=36 Caucasian | n=31  (all deceased) |
| Brooten et al (2013)(36) | 1,2 | Mixed: congenital abnormalities, prematurity head trauma, chromosomal abnormalities | n/a | 3% Black  27% White  40% Hispanic | n=47 |
| Brouwer et al (2020)(37) | 1,2 | Mixed: cancer, neurological/metabolic, cardiovascular, central nervous system and undiagnosed | n/a | n=36 “Dutch couples”  n= 6 mixed | n=44 |
| Butler et al (2018a, 2018b, 2018c, 2019)(38-41) | 1,2 | Mixed: CHD, SIDS, neurological injury, acute liver failure, acute septic shock, accident, metabolic condition, anaphylaxis, cardiac arrest | n/a | “Majority of parents Caucasian” | n=18  (all deceased) |
| Callans et al (2016)(42) | 1,2 | Not reported | n/a | Not reported | n=22 |
| Carnevale (2013)(43) | 1 | Mixed: Fontan procedure (cardiac), Cardiac shunt change, sepsis, renal transplant, trauma, maxillo-facial surgery, pleural effusion, spinal fusion, septic shock, upper airway obstruction, heart failure | Not reported | n/a | n/a |
| Carnevale et al (2011)(44) | 1,2 | Mixed: cardiomyopathy, nemaline, septic shock, asphyxia, Meningococcemia; Chromosomal encephalopathy. | n/a | Not reported | n=10 |
| Cicero-Cinecto et al (2017)(45) | 1,2 | Cancer: haematological neoplasm, extracranial solid tumour, tumour of the CNS | Not reported | Not reported | n=13  (n=7 deceased) |
| Coats et al (2016)(46) | 2 | Not reported | n/a | n=7 White  n=1 Hispanic | Not reported |
| Conway et al (2017)(47) | 1,2 | Cancer: leukaemia, solid tumour, central nervous system tumour, lymphoma | n/a | n=33 Caucasian  n=8 African American  n=4 Hispanic  n=3 Bi-racia  n=1 Asian  n=1 Indian | n=50 |
| Coyne et al (2014)(48) | 1,2 | Cancer: leukaemia, cancer of central nervous system, renal, sarcomas, lymphomas | 70% Irish  3% North Ireland  5% English  3% Trinidad and Tobago  3% South Africa  8% India  5% Philippines  3% Pakistan  3% Nigeria | 70% Irish  3% North Ireland  5% English  3% Trinidad and Tobago 3% South Africa  8% India  5% Philippines  3% Pakistan  3% Nigeria | n=20 |
| Dahav et al (2018)(49) | 1,2 | Mixed: Majority cardiac congenital disease, diaphragmatic hernia | n/a | Not reported | n=12 |
| Darbyshire et al (2015)(50) | 2 | Mixed: cancers, metabolic and genetic disorders | n/a | Not reported | n=9  (n=5 deceased) |
| Davies et al (2017)()51 | 1,2 | Cancer: lymphoblastic leukaemia | n/a | Not reported | n=35 |
| Engler et al (2020)(52) | 1,2 | Not reported | n/a | Not reported | n=9  (n=4 deceased) |
| Engvall et al (2016)(53) | 1 | Cancer: acute lymphatic leukaemia; brain tumour, sarcoma, neuroblastoma | Not reported | n/a | n/a |
| Enskar et al (2020)(54) | 1,2 | Cancer: leukaemia, brain & solid  Tumours | Not reported | Not reported | n=25  (n=1 deceased) |
| Falck et al (2016)(55) | 1,2 | Mixed: respiratory failure, renal failure, prematurity | n/a | n=3 Caucasian  n=2 African American  n=1 African | n=6 |
| Falkenburg et al  (2016, 2018)(56,57) | 1,2 | Mixed: cardiac anomaly, respiratory insufficiency, hernia, trauma, neurological disease, metabolic disorder, arteriovenous malformation and oncological | n/a | Not reported | n=20  (all deceased) |
| Fixter et al (2017)(58) | 2 | Cystic Fibrosis | n/a | n=11 White British  n=1 Asian British | n=12 |
| Gabriel et al (2019)(59) | 1,2 | Cancer: lymphoma, brain and other | n=11 Australian/New Zealand,  n=4 European,  n=1 Asian | n=9 Australian/New Zealand  n= 8 European | n=17 |
| Gilmer et al (2013)(60) | 2 | Mixed: Cardiac, congenital defects, neonatal-specific diagnoses, infectious diseases, and cancers | n/a | n=6 Caucasian  n=4 African American  n=5 unknown | Not reported |
| Greenway et al (2019)(61) | 1,2 | Not reported | n/a | n=20 Caucasian  n=6 African American  n=9 Latino  n=6 Asian/Pacific Islander  n=1 Middle Eastern | n=42 |
| Guttman et al (2020)(62) | 2 | Cerebral palsy | n/a | n=249 non-Hispanic  n=16 Hispanic | n=463 |
| Hemsley et al (2013)(63) | 1,2 | Cerebral palsy | Not reported | Not reported | n=7 |
| Hooghe et al (2018)(64) | 2 | Cancer: brain tumours, leukaemia, bone tumours, and Langerhans cell histiocytosis | n/a | Not reported | n=9 |
| Inglin et al (2011)(65) | 1,2 | Mixed: cancers, neurological disorders, and otherLT/LSC’s | n/a | Not reported | n=15  (n=6 deceased) |
| Iversen et al (2013)(66) | 1,2 | Cerebral palsy | n/a | Not reported | n=9 |
| Kelly et al (2017)(67) | 1 | Cancer: leukaemia and lymphoma, CNS tumour, solid tumour | n=13 Caucasian,  n=11 African American,  n=3 Hispanic,  n=2 Other | n/a | n/a |
| Kilicarslan-Toruner and Akgun- Citak (2013)(68) | 2 | Cancer: acute lymphoblastic leukaemia, neuroblastoma, Wilm’s tumour, non- hodgkin lymphoma, chronic myelogenous leukaemia, rhabdomyosarcoma | n/q | Not reported | 15 |
| Lamiani et al (2013)(69) | 2 | Mixed: cardiac, pneumonia, acute respiratory failure, intraventricular haemorrhage, pulmonary hypertension, sepsis meningitis, chronic renal failure, sepsis, abdominal hematoma, endocarditis and myocarditis, cerebrohepatorenal syndrome, pontocerebellar hypoplasia, kyphoscoliosis | n/a | Not reported | n=8  (all deceased) |
| Linder et al (2017)(70) | 1 | Cancer: Acute lymphoblastic leukaemia, sarcoma, Hodgkin lymphoma, acute myelogenous leukaemia, non-Hodgkin lymphoma, other solid tumour, brain tumour | n=44 White/non-Hispanic,  n=3 Hispanic,  n=1 Asian/Pacific Islander,  n=1 Native American,  n=1 other. | n/a | n/a |
| Livesley and Long (2013)(71) | 1 | Mixed: life-long illnesses and physical disabilities | Not reported | n/a | n/a |
| Mack et al (2017)(72) | 1,2 | Cancer: not reported | n/a | n=2 Hispanic  n=25, non-Hispanic  n=2 unknown | Not reported |
| Markwalter et al (2019)(73) | 2 | Not reported | n/a | n=1 Black/African American  n=19 white/Caucasian | n=20 |
| McNamara et al (2020)(74) | 2 | Not reported | n/a | n=16 white  n=4 Black | n=20 |
| Mitchell et al (2019)(75) | 1,2 | Not reported | n/a | Not reported | n=11  (all deceased) |
| Murrell et al (2018)(76) | 1,2 | SMA: Type 1 | n/a | n= 17 White/ non-Hispanicn=10 Hispanic  n=1 African American  n=1 mixed race/ethnicity | n=22  (n=12 deceased) |
| Nicholas et al (2016)(77) | 2 | Mixed: Cancer, cystic fibrosis, cerebral palsy, enzyme deficiency, and congenital heart defects | n/a | n=9 Canadian  n=9 Other | n=18  (n=6 deceased) |
| Nyborn et al (2016)(78) | 1,2 | Cancer: solid tumour, brain tumour, and hematologic malignancy | n/a | n=24 white/ non-Hispanic | Not reported |
| Obas et al (2016)(79) | 1,2 | Cardiac surgery patients: not specified | n/a | Not reported | n=9 |
| October et al (2014)(80) | 2 | Mixed: hematologic/oncologic, respiratory, neurologic, trauma, gastrointestinal, metabolic/genetic, sepsis/shock | n/a | n=28 African American  n=11 non-Hispanic  n=2 Asian  n=2 Other | n=34 |
| Orioles et al (2013)(81) | 1,2 | Mixed: cardiopulmonary, renal, neurological and cancer | n/a | n=10 Caucasian  n=1 African American  n=1 Asian  n=1 mixed race | Not reported |
| Oxley (2015)(82) | 2 | Not reported | n/a | Not reported | n=5 |
| Pinto-Taylor et al (2020)(83) | 1,2 | Mixed: Complex congenital heart disease with genetic abnormalities, Trisomy 18, multiple chromosomal abnormalities, Niemann-Pick disease and Ohtahara syndrome | n/a | Not reported | n=10  (n=8 deceased) |
| Robertson et al (2019)(84) | 1,2 | Cancer: solid, blood and brain | Not reported | Not reported | n=23 |
| Roscigno et al (2016)(85) | 1,2 | Traumatic brain injury | n/a | “All Caucasian” | n=25 |
| Ruhe et al (2016)(86) | 1 | Cancer: leukaemia, lymphoma, CNS, bone tumours, sarcoma, melanoma | Not reported | n/a | n/a |
| Saetrang et al (2019)(87) | 1,2 | Duchenne muscular dystrophy | n/a | Not reported | n=12 |
| Salmon et al (2012)(88) | 1,2 | Cancer: Acute lymphoblastic leukaemia | n/a | n=34 White British  n=1 unknown | n=35 |
| Skirko et al (2020)(89) | 2 | Pierre Robin Sequence | n/a | Not reported | n=13 |
| Smith et al (2015)(90) | 1,2 | Hydrocephalus | n/a | Not reported | n=15 |
| Smith et al (2018)(91) | 2 | Renal disease: failure | n/a | Not reported | n=14 |
| Snaman et al (2016)(92) | 1,2 | Cancer: not reported | n/a | n=10 Caucasian,  n=2 African American | Not reported  (all deceased) |
| Spalding et al (2016)(93) | 1,2 | Not reported | Not reported | Not reported | Not reported |
| Spratling et al (2012)(94) | 1 | Not reported | n=2 African American,  n=7 Caucasian,  n=1 Hispanic,  n=1 African American/Caucasian. | n/a | n/a |
| Steele et al (2013)(95) | 1,2 | Cancer: not reported | n/a | n=48 White | n=40  (all deceased) |
| Sullivan et al (2013)(96) | 2 | Mixed: Neurological disorder, neuromuscular disorder, cardiac abnormality, metabolic disease, chromosomal abnormality, muscle disorder cancer (neuroblastoma, neurofibrosarcoma), spina bifida, brain damage, cerebral palsy | n/a | Not reported | n=21 |
| Tenniglo et al (2017)(97) | 2 | Cancer: leukaemia, bone tumour, germ cell tumour, soft tissue tumour and brain tumour | Not reported | Not reported | n=11 |
| Thienprayoon et al (2016)(98) | 1,2 | Cancer: not reported | n/a | n=1 African American  n=18 Hispanic/Latino  n=15 non-Hispanic Caucasian | n=20  (all deceased) |
| Tong et al (2010)(99) | 1,2 | Renal disease: chronic | n/a | n=14 Caucasian  n=6 non-Caucasian/mixed ethnicity | n=20  (all deceased) |
| Wangmo et al (2016)(100) | 1,2 | Cancers: leukaemia, lymphomas, bone tumours, sarcomas | Not reported | Not reported | n=17 |
| Watt et al (2011)(101) | 1,2 | Cancer: leukaemia, lymphoma, sarcoma, neuroblastoma, brain tumour, Wilm’s tumour, liver cancer | n/a | “All Chinese and South Asian” | n=50 |
| Weidner et al (2011)(102) | 1,2 | Mixed: malignancy; pre-mature birth; and cardiac, neurologic, and gastrointestinal illnesses | n/a | Not reported | n=20  (all deceased) |
| Young et al (2011, 2013)(103,104) | 1,2 | Cancer: Acute lymphoblastic leukaemia (ALL) | n/a | (2011):  n=51 White British,  n=2 “ethnic minority”  (2013): Not reported | Not reported |
| Yuen et al (2012)(105) | 1,2 | Lethal epidermolysis bullosa | n/a | Not reported | n=16  (all deceased) |
| Zitzelsberger et al (2014)(106) | 1 | Renal disease: end stage | Not reported | n/a | n/a |
